# Identifying potential prescribing safety indicators related to mental health disorders and medications: A systematic review

**DOI:** 10.1371/journal.pone.0217406

**Published:** 2019-05-24

**Authors:** Wael Y. Khawagi, Douglas T. Steinke, Joanne Nguyen, Richard N. Keers

**Affiliations:** 1 Division of Pharmacy and Optometry, School of Health Sciences, Faculty of Biology, Medicine and Health, University of Manchester, Manchester, United Kingdom; 2 Clinical Pharmacy Department, College of Pharmacy, Taif University, Taif, Kingdom of Saudi Arabia; 3 Pharmacy Department, Greater Manchester Mental Health NHS Foundation Trust, Manchester, United Kingdom; Nord University, NORWAY

## Abstract

**Background:**

Prescribing errors and medication related harm may be common in patients with mental illness. However, there has been limited research focusing on the development and application of prescribing safety indicators (PSIs) for this population.

**Objective:**

Identify potential PSIs related to mental health (MH) medications and conditions.

**Methods:**

Seven electronic databases were searched (from 1990 to February 2019), including the bibliographies of included studies and of relevant review articles. Studies that developed, validated or updated a set of explicit medication-specific indicators or criteria that measured prescribing safety or quality were included, irrespective of whether they contained MH indicators or not. Studies were screened to extract all MH related indicators before two MH clinical pharmacists screened them to select potential PSIs based on established criteria. All indicators were categorised into prescribing problems and medication categories.

**Results:**

79 unique studies were included, 70 of which contained at least one MH related indicator. No studies were identified that focused on development of PSIs for patients with mental illness. A total of 1386 MH indicators were identified (average 20 (SD = 25.1) per study); 245 of these were considered potential PSIs. Among PSIs the most common prescribing problem was ‘Potentially inappropriate prescribing considering diagnoses or conditions’ (n = 91, 37.1%) and the lowest was ‘omission’ (n = 5, 2.0%). ‘Antidepressant’ was the most common PSI medication category (n = 85, 34.7%).

**Conclusion:**

This is the first systematic review to identify a comprehensive list of MH related potential PSIs. This list should undergo further validation and could be used as a foundation for the development of new suites of PSIs applicable to patients with mental illness.

## Introduction

Mental disorders are one of the largest contributors toward the global burden of disease, being responsible for 21.2% of years lived with disability (YLDs) [1] and affecting approximately 1 in 5 adults within a given 12 month period and about 1 in 3 at some point in their lives. [2] However, the quality of care provided to patients with mental illness compared to those with physical health illnesses has been found to be inferior, and their care needs may often remain unmet [3], including the management of comorbid physical conditions [4].

Medications are the most frequently used type of treatment for mental disorders [5], yet there are unique challenges when prescribing for this population. These include the enduring problem of high dose and combination antipsychotic prescribing, use of a number of high risk drugs (e.g. lithium, clozapine), the requirements of mental health law, co-existing substance misuse which may cause interactions with prescribed therapy and a high prevalence of poor lifestyle, multiple comorbidities and polypharmacy which can cause drug–disease and drug-drug interactions [6]. Taking all these factors into account, it may be difficult to achieve balanced prescribing for patients with mental illness [7].

Against this background of underlying complexity there is evidence that prescribing errors and substandard prescribing might be common in this patient group. In 2016, a Danish study found that 59% of patients admitted to a psychiatric hospital had at least one potentially inappropriate prescription (PIP), with 45% of PIPs being potentially serious or fatal [7]. In addition, a systematic review of medication errors in mental health hospitals published in 2017 reported that between 52.2–82.1% of patients may be affected by prescribing errors [8].

In order to improve the quality and safety of healthcare services provided to those with mental disorders it is important to be able to measure them. Indicators have been used widely to assess the quality of healthcare services, including prescribing. However, many prescribing indicators focus on the effectiveness of prescribing and not safety, which is important to address given the known risks prescribing can pose to patient safety [9]. Indicators that measure unsafe prescribing are known as Prescribing safety indicators (PSIs); these are statements describing potentially hazardous prescribing and drug monitoring that may put the patient at increased risk of harm. [10] Even though these prescribing patterns are not considered good practice and should generally be avoided, not all of them may necessarily be errors, and they may require judgement from the patient and clinical team. [11] The purpose of these types of indicators may therefore act as a prompt for clinical review to determine whether changes are required.

PSIs have been used to estimate the level of variation in prescribing safety between practices [12], to observe change after interventions [13], and to develop clinical decision support (CDS) alerts in computerized provider order entry (CPOE) [14, 15]. Awareness of the potential value of PSIs has grown, with recent deployment in England of a national medication safety dashboard to monitor a limited set of PSIs to inform safer prescribing [16]. Elsewhere, PSIs have driven the development of the successful pharmacist-led information technology intervention for medication errors (PINCER) approach [17] which now features in UK National Institute for Health and Care Excellence (NICE) guidance for medicines optimisation [18]. However, whilst numerous sets of prescribing quality and safety indicators and inappropriate prescribing criteria have been developed for different populations and settings [19, 20], mental health illnesses and the medications used to treat them have not received as much attention in this regard.

Whilst there are a number of informative academic papers describing the development of broad suites of PSIs across primary [10, 21] and secondary care [15] that include some mental health related indicators, these were not developed to be used specifically for populations with mental illness. In addition, existing systematic reviews of broader categories of prescribing indicators [19, 20] have only identified one existing mental health specific set of prescribing quality indicators [22]. However, this set may not reflect current practice since it was published 14 years ago, and does not address many known areas of potentially hazardous prescribing in those with mental illness such as medication monitoring issues and omissions [22]. Previous systematic reviews were also affected by limitations, such as not including all known types of prescribing assessment tools [19, 20]. It is therefore of importance that existing prescribing indicators and suites of all kinds that are relevant to those with mental illness are identified and those considered to be potential PSIs subsequently extracted, as without a suitable tool in place efforts to improve the safety of health care may be limited in this population.

The aim of this systematic review was therefore to identify comprehensively from the existing literature published prescribing indicators and suites of all kinds from across all settings, and to extract from these any individual potential prescribing safety indicators or whole tools that are related to mental health disorders and medications.

## Methods

**Fig 1:**
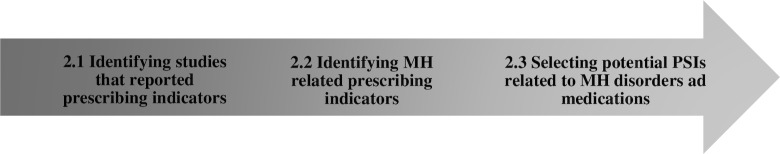
Systematic review stages. MH = Mental health. PSI = Prescribing Safety Indicators.

In order to achieve the aim of this systematic review, we followed three stages (Fig 1); (1) identifying studies that reported prescribing indicators of any kind; (2) identifying and extracting mental health (MH) related prescribing indicators; and (3) selecting potential PSIs related to MH disorders and medications.

### Stage 1: Identifying studies that reported prescribing indicators of any kind

#### Database search strategy

A systematic search was conducted using the following electronic databases: Embase, MEDLINE, PsycINFO, Web of Science, Health Management Information Consortium (HMIC), International Pharmaceutical Abstracts (IPA) and Cumulative Index to Nursing and Allied Health Literature (CINAHL). The search strategy was designed using Medical Subject Headings (MeSH) and free text words tailored to each database (S1 File). Three sets of search terms were combined; medication safety terms, quality measure terms and indicators development/validation terms. The search timeframe was limited from January 1990 to February 2019, since one of the earliest examples of inappropriate prescribing explicit criteria was published in 1991 by Beers [23, 24]. The bibliographies of included studies and of relevant review articles were reviewed manually to identify additional citations.

The search results were assessed for eligibility by screening the title and abstract by one reviewer (WK). Afterwards, the full-texts of potentially relevant articles were each reviewed for inclusion by WK. Any uncertainty regarding the eligibility of an article was discussed by the research team until consensus was reached.

#### Definitions

The term ‘indicator’ was used to describe all the different types of prescribing indicator/criteria. Explicit indicators were included in the study and can be described as drug- or disease-oriented indicators that can be applied as firm standards (e.g. prescribing Benzodiazepines for ≥ 4 weeks for elderly patients [25]). Implicit indicators are person-specific, and their use requires professional skills (e.g. is there an indication for the drug? [26]) and were not included in this review.

#### Inclusion criteria

Articles were eligible for inclusion if they developed, validated or updated a set of explicit indicators or criteria that measured prescribing in terms of safety or quality, including inappropriate prescribing, prescribing errors, hazardous prescribing, prescribing faults, monitoring errors or any other term that might be used to describe prescribing safety or quality. As the initial aim was to capture all relevant materials so that mental health indicators could be identified, there were no restrictions on the type of study design, targeted setting, the age group the indicators were intended for use in, publication language and intended country for deployment. All relevant articles were included whether they featured any mental health related indicators or not.

#### Exclusion criteria

We excluded articles that developed implicit indicators only (e.g. is there an indication for the drug? [26]), because they were not drug- or disease-oriented. We also excluded articles that developed indicators based on aggregate data and did not have any relation to patient level data (e.g. Ratio of co-trimoxazole items to trimethoprim items [27]). Studies that developed indicators non-specific to a medication or therapeutic class were also excluded (e.g. If the duration of a drug is outside the range stated in the British National Formulary (BNF) [28]), as were conference abstracts unless we were able to obtain the full indicator list. Studies that measured the prevalence of prescribing quality or safety, using a previously published prescribing indicator suite/tool without further development were considered duplicates and were not included, as were those involving adaptation/translation of single published prescribing indicator suite/tool to be used in another country without further development. Studies describing sets of indicators exclusively limited to a specific disease or specific therapeutic drug class that were not related to mental health medications and/or illnesses were also excluded (e.g. prescribing quality indicators for patients with type 2 diabetes [29]), as were those studies whose main focus was not prescribing (e.g. assessing care of vulnerable elders (ACOVE) quality indicators [30]).

#### Data extraction

The data extraction process for each study was conducted independently by two authors into a standardised and piloted electronic data extraction sheet. Discrepancies were discussed by the research team until agreement was reached. The following data were extracted from each included study where presented: **Study information:** Study title, main author, country, aim of the study. **Study design:** Setting, targeted population, indicators sources, validation methods. **Results**: Total number and type of indicators.

#### Quality assessment

Due to the heterogeneity of the included studies objectives and methods, we did not formally assess the methodological quality of the included studies. In addition, even though most studies used a consensus approach to develop their indicators, to our knowledge, there are no formal tools to assess the quality of consensus-based studies. However, certain aspects of the quality of the included studies are discussed later in this paper, such as the methods used to select indicators and the process to validate the indicators.

### Stage 2: Identifying and extracting MH related prescribing indicators

All included studies from the first stage were screened to identify and extract all mental health related indicators based on the definition in Box 1.

**Box 1. mental health related indicators definition**

Indicators were defined as mental health related if they included:

1. A medication that can be used to treat or prevent any mental health condition (e.g. prescribing atypical antipsychotic for elderly **[31, 32]**), unless the indicator was specific for a non-mental health indication (e.g. clonidine for the treatment of arterial hypertension in the elderly **[33]**),
2. A medication that can be used to treat or prevent side effects of any of the medications that can be used to treat or prevent any mental health condition (e.g. Trihexyphenidyl for treatment of extrapyramidal symptoms caused by antipsychotics for elderly **[34]**), unless the indicator were specific for a non-mental related health indication, or
3. A drug-disease interaction of any medication with any mental health condition (e.g. H2 receptors antagonist **[34]** or antimuscarinic drugs **[25]** with dementia, or chronic cognitive impairment in elderly).

The following information sources were used to determine the uses of each medication when screening for mental health related indicators: British National Formulary, Martindale, AHFS Drug Information (all accessed via Medicines complete[35]). In addition, International Classification of Diseases, 10th revision (ICD-10) Chapter 5: Mental and behavioural disorders [36] and Diagnostic and Statistical Manual of Mental Disorders, 5th Edition (DSM-5) [37] were used to determine mental health conditions.

Some indicators were considered mental health related because they included medication within a wider therapeutic class that could be used to treat mental health conditions, such as first-generation antihistamines. It was not always clear whether all medication within certain classes may be used to treat mental health disorders, however the class was included due to variation between clinical practice in different countries but only if more than one medication within that class was identified as being used in the treatment of mental illness. Conversely, some other classes were not included entirely as mental health related, because only one of the medications within that class could be used in the treatment of mental illness (e.g. clonidine).

After identifying all mental health related indicators, duplicates were removed, and if an indicator included more than one medication, class or condition it was split into more than one. For example, *“Benzodiazepine or benzodiazepine-like drug prescribed to a patient with chronic obstructive pulmonary disease [15]”*, was split into two indicators, one for benzodiazepine and another for benzodiazepine-like drug. In addition, in regards to the identified outcome indicators, these included an adverse outcome that was caused by a pattern of care (for example: Outcome: Fall and/or hip fracture and/or other bone fracture and/or bone break, Process of care: Use of a long-half-life hypnotic-anxiolytic [38]). For such indicators, we only extracted the process of care that leads to the outcome in our list of potential indicators.

The identified mental health related indicators were categorised according to the type of prescribing problem (potentially inappropriate medication (PIM): independent of diagnoses or conditions, PIM: considering diagnoses or conditions, drug-drug interaction (DDI), inappropriate dosing, inappropriate duration, inadequate monitoring and omission) (Table 1), these categories were adapted from previous studies. [39–41]. Identified indicators were also categorised to their therapeutic class (Antipsychotics, Antidepressants, Sedatives, hypnotics and anxiolytics, attention deficit hyperactivity disorder (ADHD) medications, Anti-dementia, Mood stabilisers, Non-specific anticholinergics and Non-specific psychotropics). The numbers and percentages of the indicators in each category were calculated.

**Table 1:** Descriptions and examples of the types of prescribing problems.

Type of prescribing problem	Description	Example
**PIM: independent of diagnoses or conditions**	Medication/class that is potentially prescribed inappropriately to a specific population	Prescribing antipsychotics to patients aged ≥65 [25, 34, 38, 42, 43]
**PIM: considering diagnoses or conditions**	Medication/classes that is potentially prescribed inappropriately with a specific diagnose or condition.	Prescribing antipsychotics for patients with dementia and aged ≥65 [34]
**DDI**	Medication/classes that is potentially interacts with another medication/class	Prescribing antipsychotics with antiparkinsonian for patients aged ≥65 [44]
**Inappropriate dosing**	Medication that was prescribed in inappropriate dose	Prescribing Haloperidol at a dose >2 mg for patients aged ≥65 [45–47]
**Inappropriate duration**	Medication/class that was prescribed in inappropriate duration	Prescribing antipsychotics for >1 month to patients aged ≥65 [48]
**Inadequate monitoring**	Medications/class that was not monitored adequately	Prescribing lithium without monitoring lithium level every 6 months [10, 49, 50]
**Omission**	Medication/class that should be prescribed with a specific diagnose or condition.	Patients diagnosed with mild-moderate Alzheimer’s dementia and aged ≥65 and were not prescribed acetylcholinesterase inhibitor [25]

DDI = drug-drug interaction. PIM = Potentially inappropriate medication.

### Stage 3: Selecting potential PSIs related to MH disorders and medications

Following the identification and extraction of all mental health related indicators as described in the second stage, two experienced mental health pharmacists (RK and JN) together reviewed the identified list and used respected recourses, such as NICE guidelines [51], the Maudsley Prescribing Guidelines in Psychiatry [52], Psychotropic Drug Directory [53], Stockley’s Drug Interactions [35] and the resources described in stage two along with their clinical knowledge to select potential PSIs that met our adapted [10] definition: statements that described a pattern of potentially hazardous prescribing or drug monitoring that could cause significant risk of harm. Our definition differed to the original in that we did not focus on prescribing specific to the UK and we did not consider data extraction feasibility due to the likelihood of different health care record/prescribing systems being used across the globe.

When selecting PSIs, if more than one indicator shared similar characteristics, the broader indicator was selected. For example, if an indicator was found for a class of medication but other indicators for specific medications existed within that class, only the former was selected as PSI. Another example, an indicator for elderly versus an indicator for all ages. If the risk of harm was relevant for all populations, then the latter was selected. This step was performed to reduce the large number of identified PSIs by removing similar indicators with slight variations. PSIs were also categorised according to the type of prescribing problem and to their therapeutic class as described for general MH related indicators in stage two.

### Data analysis

A descriptive analysis of the findings was presented. The extracted information was presented in tabular form. Numbers and percentages were calculated when appropriate. In addition, the average number of reported indicators and standard deviation were provided.

## Results

### Stage 1: Identifying studies that reported prescribing indicators of any kind

**Fig 2:**
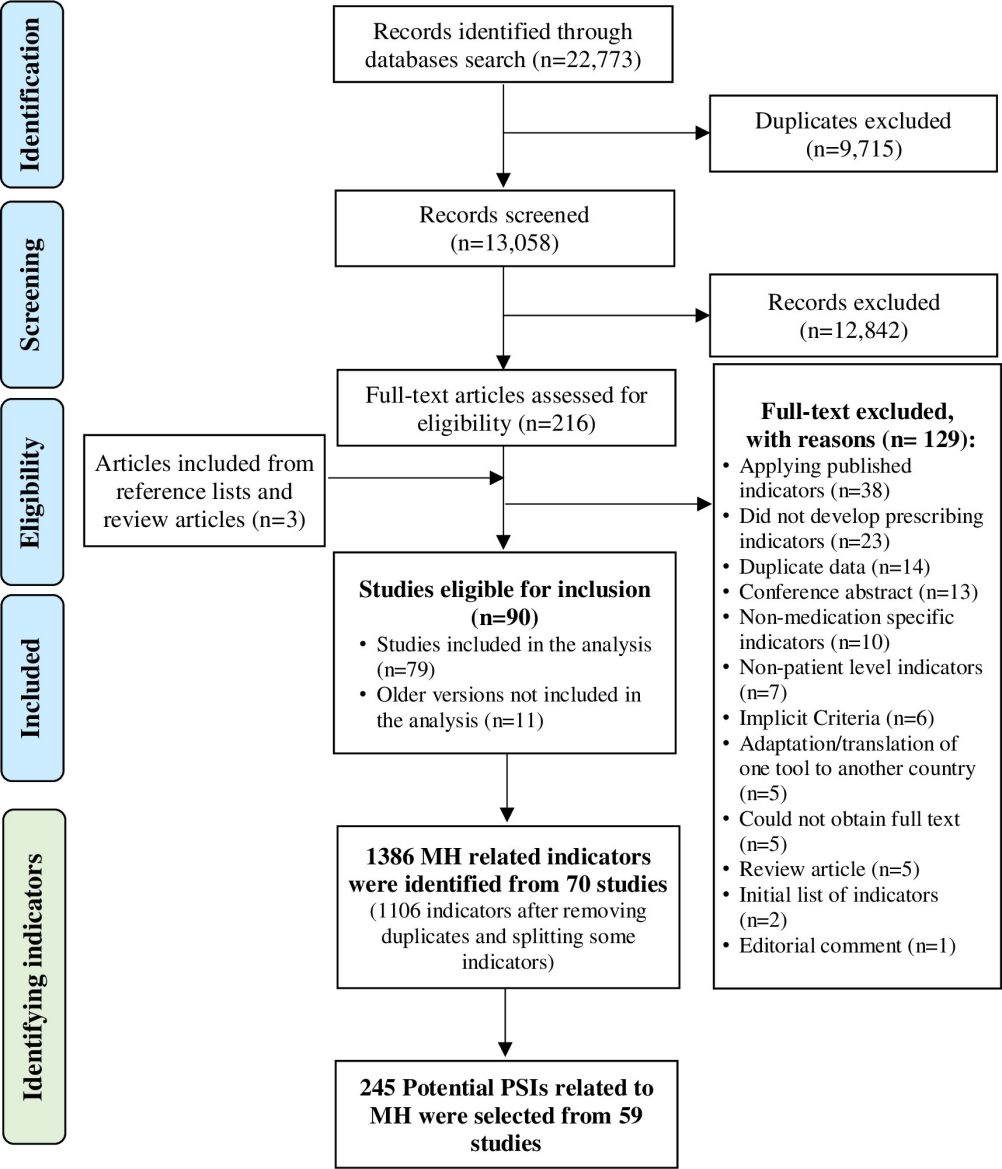
Flow diagram of the review process. MH = Mental health. PSI = Prescribing Safety Indicators.

The database search process identified 22,773 citations. Of these, 9,715 studies were removed because of duplication. The remaining 13,058 citations were screened for eligibility, where 12,842 were subsequently excluded. Hence, 216 full texts were retrieved for in-depth review. Of these, 129 were excluded leaving 87 studies for inclusion. After reviewing the reference lists of included studies and relevant reviews a further 3 studies were included, bringing the final number of the eligible studies to 90. However, 11 studies [21, 23, 33, 40, 54–60] were older versions of new articles, and only their most recent versions were included. Therefore, 79 unique studies were included in the analysis. A summary of the review process is shown in Fig 2. Table 2 summarises the information extracted from each included study. Table 3 summarises the characteristics of the 79 unique studies.

**Table 2:** Summary of each included study.

Author Year	Targeted Country(s)	Targeted Setting	Targeted Population	Indicators Source	Validation Method	Type of Criteria/Indicators		No. of indicators	No. of MH indicators
						P/O	The used term		
**AGS** 2015 [34]	USA	MS	Elderly	Literature review + older version [56]	Delphi^M^	P	PIM, DDI, DSI	231	125
**Older versions Beers** 1991 [54] **Beers** 1997 [55] **Fick** 2003 [40] **AGS** 2012 [56]									
**Al-Taweel** 2017 [49]	International	MS	Adults with Bipolar disorder	Guidelines	NS consensus	P	Adherence to management guidelines	26	26
**Alldred** 2008 [61]	UK	LTC	Elderly	Guidelines + experience	NS consensus	P	Medication monitoring errors	25	3
**Avery** 2009 [17]	UK	Community	NS	NR	NR	P	Hazardous prescribing and inadequate monitoring	10	1
**Barnett** 2014 [62]	UK	Community	NS	Selected previously published studies	NS consensus	P	High risk prescribing	6	1
**Barry** 2016 [63]	UK and Ireland	Community	Paediatric	Literature review	Delphi^M^	P	PIP	12	0
**Basger** 2012 [64]	Australia	MS	Elderly	Older version [23]	RAM	P	DRPs (Prescribing appropriateness)	41	6
**Older version Basger** 2008 [23]									
**Castillo-Páramo** 2013 [43]	Spain	Community	Elderly	STOPP / START 2008 [59]	RAM	P	PIM, PPO	86	21
**Caughey** 2014 [65]	Australia	Hospitals	NS	Literature review	RAM^M^		Preventable medication-related hospitalisations	29	1
**Chang** 2012 [66]	Taiwan	MS	Elderly	Selected previously published studies	Delphi^M^	P	PIM, DSI	182	68
**Chen** 2005 [67]	UK	Community	NS	Textbooks	NR	P	DDI, DSI	213	NR
**Clyne** 2013 [68]	Ireland	Community	Elderly	Selected previously published studies	NS consensus	P	PIP	39	14
**Constantine** 2013 [69]	USA	NS	All ages	Guidelines	Expert Panel	P	Unusual prescribing	12	10
**Cooper** 2014 [70]	UK and Ireland	NS	Middle aged	Selected previously published studies + Experience	Delphi	P	PIP	22	7
**Desnoyer** 2017 [71]	International	Hospitals	Adults	Literature review + Experience	Delphi	P	PIM	160	22
**Desrochers** 2011 [72]	Canada	Pharmacies	CKD patients	Literature review + Experience	RAM	P	DRPs	50	2
**Dreischulte** 2012 [73]	UK	Community	NS	Literature review	RAM^M^	P	High risk and suboptimal prescribing and monitoring	176	16
**Elliott** 2001 [74]	Australia	Hospitals	Elderly	Selected Previously published studies + Experience	Expert panel	P	PQ (Prescribing appropriateness)	19	3
**Fernández Urrusuno** 2013 [75]	Spain	Community	NS	Guidelines	NGT	P	PQ	14	1
**Fialová** 2013 [47]	Czech	NS	Elderly	Literature review	Delphi^M^	P	PIM, DSI	121	48
**Fox** 2016 [76]	UK	Hospitals	Paediatric	Thomas study [15] + Literature review + Local and national incidents + NPSA alerts	Delphi	P	PE (high risk prescribing)	41	0
**Galán Retamal** 2014 [77]	Spain	Hospitals	Elderly	Selected previously published studies	Delphi	P	PIM	50	15
**Guerreiro** 2007 [50]	Portugal	Community	NS	Selected previously published studies	Delphi	P	PDRM	35	4
**Guthrie** 2011 [11]	UK	Community	NS	Literature review	RAM^M^	P	High risk (Hazardous) prescribing	9	2
**Hanora Lavan** 2017 [78]	Ireland	MS	Elderly with Limited life expectancy	Literature review + Experience	Delphi	P	PIP or PIM	27	2
**Harper** 2014 [79]	USA	Hospitals	Paediatric	NR	NS consensus	P	DDI	19	7
**Holmes** 2008 [80]	USA	LTC	Palliative with advanced dementia	Textbooks	Delphi^M^	P	Medication appropriateness categories	54	54
**Holt** 2010 [45]	Germany	NS	Elderly	Literature review + selected previously published studies	Delphi^M^	P	PIM	83	51
**Hurley** 2005 [81]	USA	Community	Adults	Textbooks + FDA black box warnings + Guidelines	NR	P	Medication monitoring	24	11
**Khodyakov** 2017 [32]	USA	LTC	Elderly	STOPP/START 2015 [25]	Delphi^M^	P	PIM, PPO	24	9
**Kim** 2015 [82]	Korea	Community	NS	WHO-ATC classification + the Korean National Health Insurance criteria for pharmacy benefits + guidelines	Delphi	P	Duplication	33	0
**Kim** 2015 [83]	Korea	NS	Elderly	Selected previously published studies	Delphi	P	PIM (DSI)	26	18
**Kim** 2018 [84]	Korea	MS	Elderly	Selected previously published studies + Older version	Delphi^M^	P	PIM	110	54
**Older version** **Kim** 2010 [60]									
**Kojima** 2016 [85]	Japan	NS	Elderly	Literature review	NS consensus	P	PIM, PPO	37	9
**Kroger** 2015 [86]	Canada	LTC	Patients with severe dementia	Literature Review	RAM^M^	P	Medication appropriateness categories	49	49
**Laroche** 2007 [87]	France	NS	Elderly	Literature review	Delphi	P	PIM	34	19
**Lindblad** 2006 [88]	USA	Community	Elderly	Literature Review	Delphi	P	DSI	28	19
**Mackinnon** 2002 [38]	USA and Canada	NS	Elderly	Literature Review	Delphi	O	PDRM	52	17
**Maio** 2010 [31]	Italy	Community	Elderly	Beers 2003 [40]	NGT	P	PIP	23	5
**Malone** 2004 [89]	USA	Pharmacies	NS	Literature Review + DDI resources	Delphi^M^	P	DDI	25	11
**Mann** 2012 [90]	Austria	MS	Elderly	PRISCUS preliminary list	Delphi^M^	P	PIM	73	37
**Marzi** 2018 [91]	Argentina	NS	Elderly	Literature review + selected previously published studies	Delphi	P	PIM	128	63
**Mast** 2015 [92]	Netherlands	Community	Elderly	Literature review + guidelines + experience	Delphi	P	DRPs	124	16
**McLeod** 1997 [93]	Canada	NS	Elderly	Textbooks + Beers 1991 [54]	Delphi^M^	P	PIP	38	14
**Morris** 2003 [94]	UK	Community	NS	Older version + Selected previously published studies	Delphi	O	PDRM	24	0
**Older version Morris** 2002 [57]									
**Nyborg** 2015 [95]	Norway	LTC	Elderly	NORGEP criteria [96] + Literature review + Experience.	Delphi	P	PIM	34	17
**O'Mahony** 2015 [25]	Europe	MS	Elderly	Older version [59] + Literature review + Experience.	Delphi	P	PIM, PPO	114	25
**Older version Gallagher** 2008 [59]									
**Oborne** 1997 [97]	UK	Hospitals	Elderly	Literature Review	Expert panel	P	Harmful and appropriate Prescribing	14	0
**Oborne** 2003 [98]	UK	LTC	Elderly	Selected previously published studies	NR	P	Harmful and Appropriate Prescribing	13	0
**Okechukwu** 2006 [99]	Ireland	Community	NS	Literature Review	NS consensus	P	PQ	11	1
**Onder** 2014 [44]	Italy	NS	Elderly	Literature Review	Delphi^M^	P	Poor Prescribing Quality	13	1
**Onder** 2014 [100]	International	MS	Complex Elderly	Literature review + Guidelines	NS consensus	P	Recommendations to Prescribe	19	0
**Paton** 2004 [22]	UK	Hospitals	Psychiatric patients	NR	NR	P	PQ	7	5
**Pazan** 2018 [101]	Europe	NS	Elderly	Older version [58]	Delphi	P	Medication appropriateness categories	264	63
**Older version** **Kuhn-Thiel** 2014 [58] Pazan 2016 [33]									
**Phansalkar** 2011 [102]	USA	Pharmacies	NS	Selected previously published studies + Medications databases	NS consensus	P	DDI	15	7
**Prot-labarthe** 2014 [103]	France	NS	Paediatric	Literature Review	Delphi	P	PIM, PPO	102	9
**Quintense** 2019 [104]	Belgium	Hospitals	NS	Literature review + Guidelines	Expert panel	P	Clinical rules	78	8
**Rancourt** 2004 [41]	Canada	LTC	Elderly	Literature Review	Delphi^M^	P	PIP	111	53
**Raebel** 2006 [105]	USA	Community	NS	FDA black-box warnings + Guidelines + Experience	NR	P	Medication monitoring	12	2
**Reabel** 2007 [106]	USA	Community	Elderly	Selected previously published studies	Expert panel	P	PIM	11	5
**Renom-Guiteras** 2015 [46]	Europe	NS	Elderly	Selected previously published studies	Delphi	P	PIM	282	127
**Robertson** 2002 [107]	Canada	NS	Elderly	Mackinnon study [38] + Experience	Delphi and NGT	O	PDRM	52	15
**Rognstad** 2009 [96]	Norway	Community	Elderly	Literature Review + Experience	Delphi^M^	P	PIP (PIM, DDI)	36	22
**Ruths** 2003 [108]	Norway	LTC	Elderly	Literature Review + Guidelines + Experience	Expert panel	P	DRPs	17	7
**Saverno** 2011 [109]	USA	Pharmacies	NS	Literature Review + DDI references	Consensus among the researchers	P	DDI	13	1
**Smits** 2016 [110]	Netherlands	MS	CKD patients	Guidelines + Literature review	RAM	P	Optimal and unsafe prescribing	16	0
**Solberg** 2004 [111]	USA	Community	Adults	3 key DDI references	Expert panel	P	DDI	44	17
**Spencer** 2014 [10]	UK	Community	NS	Literature review + older version [21] + Textbooks	RAM	P	Hazardous prescribing and inadequate monitoring.	56	7
**Older version Avery** 2011 [21]									
**Tamblyn** 1994 [112]	Canada	MS	Elderly	Literature Review + Experience + Textbooks	Expert panel	P	High risk prescribing and DDI	32	17
**Thomas** 2013 [15]	UK	Hospitals	NS	literature review + Experience	Delphi	P	PE (high risk prescribing)	80	18
**Tjia** 2010 [113]	USA	Community	Adults	Literature Review + FDA black-box warnings + Guidelines	Delphi^M^	P	Medication monitoring	61	13
**Tommelein** 2015 [48]	Belgium	Pharmacies	Elderly	Literature Review	RAM	P	PIP	83	18
**Van der Linden** 2014 [42]	Belgium	NS	Elderly	STOPP 2008 [59]	NS consensus	P	PIP	76	11
**Van Dijk** 2003 [114]	Netherlands	LTC	Elderly	NR	NR	P	Suboptimal prescribing	17	1
**Wessell** 2010 [39]	USA	Community	Adults	Literature Review	NS consensus	P	Prescribing and Monitoring errors	30	8
**Williams** 2005 [115]	Ireland	Community	NS	Literature Review	NS consensus	P	Harmful and Appropriate Prescribing	16	1
**Winit Watjana** 2008 [116]	Thailand	NS	Elderly	Literature Review + Textbooks	Delphi	P	High-risk medications, DDI and DSI	77	28
**Yu** 2011 [117]	USA	Hospitals	NS	Literature Review + Experience	Delphi^M^	P	Medication monitoring	24	1
**Zhan** 2001 [118]	USA	Community	Elderly	Beers 1997 [55]	Delphi^M^	P	PIM	33	17

ATC: The Anatomical, Therapeutic and Chemical. CKD: Chronic kidney disease. DDI: drug-drug interaction. DRPs: Drug related problems. DSI: drug-disease interaction. FDA: Food and Drug Administration. LTC: Long-term care. ^M^: Modified. MH: Mental Health. NGT: Nominal group technique. NORGEP: The Norwegian General Practice. NPSA: National Patient Safety Agency. NR = not reported. NS = not specified. O = Outcome (outcome indicator is the consequences of provided healthcare). P = Process (process indicators comprises the care provided to the patients). P/O = Process/Outcome. PDRM: preventable drug related morbidity. PE: prescribing errors. PIM: potentially inappropriate medication. PIP: potentially inappropriate prescribing. PPO: potentially prescribing omission. PQ: prescribing quality. RAM: RAND/UCLA Appropriateness Method. STOPP/START: Screening tool of older people's prescriptions and screening tool to alert to right treatment. UK: United Kingdom. USA: United States of America. WHO: World Health Organization.

**Table 3:** Summary of included study characteristics.

Characteristics	All unique studies	Studies included MH-related indicators	Studies MH-related potential PSIs were selected from
	(79 studies)	(70 studies)	(59studies)
	N (%)	N (%)	N (%)
***Continent***			
Europe	42 (53.2%)	35 (50.0%)	27 (47.5%)
North America	24 (30.4%)	24 (34.3%)	22 (37.3%)
Asia	6 (67.7%)	5 (7.1%)	5 (8.5%)
International	3 (3.8%)	2 (2.9%)	2 (3.4%)
Australia	3 (3.8%)	3 (4.3%)	1 (1.7%)
South America	1 (1.3%)	1 (1.4%)	1 (1.7%)
***Publication Year***			
1990–1999	3 (3.8%)	2 (2.9%)	2 (3.4%)
2000–2009	26 (32.9%)	23 (32.9%)	18 (30.5%)
2010–2019	47 (63.3%)	45 (64.3%)	39 (66.1%)
***Targeted population***			
Elderly	40 (50.6%)	38 (54.3%)	31 (52.5%)
Not specified	20 (25.3%)	17 (24.3%)	15 (25.4%)
Adults	5 (6.3%)	5 (7.1%)	5 (8.5%)
Paediatric	4 (5.1%)	2 (2.9%)	2 (3.4%)
CKD	2 (2.5%)	1 (1.4%)	1 (1.7%)
All ages	1 (1.3%)	1 (1.4%)	1 (1.7%)
Middle aged	1 (1.3%)	1 (1.4%)	1 (1.7%)
Psychiatric	1 (1.3%)	1 (1.4%)	1 (1.7%)
Adults with bipolar disorder	1 (1.3%)	1 (1.4%)	1 (1.7%)
Severe dementia	1 (1.3%)	1 (1.4%)	1 (1.7%)
Elderly with Limited life expectancy	1 (1.3%)	1 (1.4%)	-
Palliative with advanced dementia	1 (1.3%)	1 (1.4%)	-
Complex elderly	1 (1.3%)	-	-
***Targeted setting***			
Community	26 (32.9%)	22 (31.4%)	19 (32.2%)
Not specified	17 (21.5%)	17 (24.3%)	16 (27.1%)
Hospitals	11 (13.9%)	9 (12.9%)	8 (13.6%)
Multiple settings	11 (13.9%)	9 (12.9%)	6 (10.2%)
Long-term care	9 (11.4%)	8 (11.4%)	5 (8.5%)
Pharmacies	5 (6.3%)	5 (7.1%)	5 (8.5%)
***Methods to identify indicators*^a^**	**Reported 75 (94.9%)**	**Reported 66 (94.3%)**	**Reported 56 (94.9%)**
Literature review	41 (51.9%)	36 (51.4%)	33 (55.9%)
Experience	16 (20.3%)	16 (22.9%)	13 (22.0%)
Multiple selected tools ^b^	16 (20.3%)	14 (20.0%)	11 (18.6%)
Guidelines	12 (15.2%)	9 (12.9%)	8 (13.6%)
Single selected tool ^c^	9 (11.4%)	7 (10.0%)	6 (10.2%)
Textbooks ^d^	7 (8.9%)	6 (8.6%)	5 (8.5%)
Older versions	7 (8.9%)	6 (8.6%)	5 (8.5%)
FDA black box warnings	3 (3.8%)	3 (4.3%)	3 (5.1%)
DDI references	3 (3.8%)	3 (4.3%)	3 (5.1%)
medication databases	1 (1.3%)	1 (1.4%)	1 (1.7%)
preliminary list	1 (1.3%)	1 (1.4%)	-
Safety incidents	1 (1.3%)	-	-
***Validation method***	**Reported 72 (91.1%)**	**Reported 65 (92.9%)**	**Reported 55 (93.2%)**
Delphi	38 (48.1%)	34 (48.6%)	29 (49.2%)
NS consensus	12 (15.2%)	11 (15.7%)	10 (16.9%)
RAM	10 (12.7%)	9 (12.9%)	8 (13.6%)
Expert panel	8 (10.1%)	7 (10.0%)	5 (8.5%)
NGT	2 (2.6%)	2 (2.9%)	1 (1.7%)
Consensus among research group	1 (1.3%)	1 (1.4%)	1 (1.7%)
Delphi and NGT	1 (1.3%)	1 (1.4%)	1 (1.7%)
***Type of prescribing indicators***			
Process	75 (94.9%)	67 (95.7%)	56 (94.9%)
Outcome	4 (5.1%)	3 (4.3%)	3 (5.1%)
***Number of indicators***	**4507 reported indicators**	**1386 MH related indicators** **(1106 after removing duplicates and splitting indicators)**	**245 MH related PSIs** ^e^
Average (SD)	57 (SD = 59.8)	20 (SD = 25.1)	-
Range	6–282	1–127	-

CKD: Chronic kidney disease. DDI: Drug-drug interactions. FDA: Food and Drug Administration. MH: Mental health. NGT: nominal group technique. NS: not specified. PSIs: Prescribing safety indicators. RAM: RAND/UCLA Appropriateness Method. SD: Standard deviation

^**a**^. The total percentage exceed 100% because most studies used more than one method.

^**b**^. These studies selected multiple previously published tools.

^**c**^. These studies selected one specific tool

^**d**^. These studies used selected textbooks.

^**e**^. The average, SD and range were not calculated for the potential PSIs because they were selected after removing duplicates and splitting indicators.

### Stage 2: Identifying and extracting MH related prescribing indicators

From the 79 included unique studies, a total of 4507 individual prescribing indicators were reported containing an average of 57 (SD = 59.8) indicators per study, ranging from 6 [62] to 282 [46] indicators.

Seventy studies (88.6% of unique studies) contained at least one mental health related indicator. Following data extraction and review, a total of 1386 (30.8% of total) indicators were deemed to be mental health related based on our operational definition (Box 1). There was an average of 20 (SD = 25.1) mental health related indicators per study, and ranging from 1 [17, 44, 62, 65, 75, 99, 109, 114, 115, 117] to 127 [46] indicators. Five studies were concerned exclusively with prescribing indicators in the mental health population/setting [22, 49, 69, 80, 86]. Nine studies did not report any mental health prescribing indicators [63, 67, 76, 82, 94, 97, 98, 100, 110]. Table 3 summarises the characteristics of the studies that included mental health related prescribing indicators (n = 70).

#### Countries

Most studies developed prescribing indicator tools to be used in the United States of America (USA) [32, 34, 39, 69, 79–81, 88, 89, 102, 105, 106, 109, 111, 113, 117, 118] (n = 17/70, 24.3%), followed by the United Kingdom (UK) [10, 11, 15, 17, 22, 61, 62, 73] (n = 8, 11.4%) and Canada [41, 72, 86, 93, 107, 112] (n = 6, 8.6%). The remaining studies described tools developed for Ireland [68, 78, 99, 115] (n = 4, 5.7%), Spain [43, 75, 77] (n = 3, 4.3%), Australia [64, 65, 74] (n = 3, 4.3%), Norway [95, 96, 108] (n = 3, 4.3%), Belgium [42, 48, 104] (n = 3, 4.3%), The Netherlands [92, 114] (n = 2, 2.9%), Italy [31, 44] (n = 2, 2.9%), France [87, 103] (n = 2, 2.9%), Korea [83, 84] (n = 2, 2.9%), Germany [45] (n = 1, 1.4%), Taiwan [66] (n = 1, 1.4%), Austria [90] (n = 1, 1.4%), the Czech Republic [47] (n = 1, 1.4%), Portugal [50] (n = 1, 1.4%), Japan [85] (n = 1, 1.4%), Argentina [91] (n = 1, 1.4%) and Thailand [116] (n = 1, 1.5%). Another 7 studies developed tools to be used in more than one country; 3 (4.3%) [25, 46, 101] were for European countries, 2 (2.9%) [49, 71] were for international use, 1 (1.4%) [70] were for the UK and Ireland, and 1 (1.4%) [38] was for Canada and the USA.

#### Publication year

Only 2 studies (2.9%) [93, 112] were published prior to the year 2000. A total of 23 (32.9%) studies were published between 2000–2009, and 45 (64.3%) from 2010 onwards.

#### Targeted population

The elderly population was the most common patient group specifically targeted by the indicator tools (n = 38/70, 54.3%). Of these, 26/38 (68.4%) [25, 31, 32, 34, 41, 43–47, 61, 64, 66, 74, 77, 83, 84, 88, 90, 92, 101, 106–108, 112, 118] studies defined their elderly population as ≥65 years old, 3 (7.9%) [68, 95, 96] as ≥70 years old, 2 (5.3%) [85, 87] as ≥75 years old, and the remaining 7 (18.4%) [38, 42, 48, 91, 93, 114, 116] tools did not define a specific age. Of the remaining studies, 5/70 (7.1%) [39, 71, 81, 111, 113] described tools specifically for adults, 2 (2.9%) [79, 103] for paediatric patients, 4 (5.7%) for psychiatric patients (including bipolar disorder (n = 1),[49] general psychiatric patients (n = 1)[22] and severe/advanced dementia (n = 2)[80, 86]), and 1 (1.4%) [72] for patients with chronic kidney disease. Another 3 indicator tools specifically targeted either middle age (45–46 years old) patients [70], patients of all ages [69] and patients with limited life expectancy [78]. A total of 17 (24.3%) [10, 15, 17, 50, 62, 65, 73, 75, 89, 99, 102, 104, 105, 109, 115, 117, 119] of the 70 studies did not identify a population that their indicators were meant to be applied to.

#### Setting

A total of 22 (31.4%) studies developed tools that were specific to patients in the community, including primary care (n = 14, 20.0%)[10, 11, 17, 39, 43, 50, 62, 68, 73, 75, 92, 96, 99, 115], ambulatory care (n = 5, 7.1%) [81, 105, 106, 111, 113] and 3 studies (4.2%) [31, 88, 118] targeted any patients in the community.

Seventeen (24.3%) studies did not specify a setting for their developed tools. The remaining tools targeted hospitals (n = 9/70, 12.9%) [15, 22, 65, 71, 74, 77, 79, 104, 117], multiple settings (n = 9, 12.9%) [25, 34, 49, 64, 66, 78, 84, 90, 112], long-term care settings (n = 8, 11.8%) [32, 41, 61, 80, 86, 95, 108, 114] and pharmacies (n = 5, 7.1%) [48, 72, 89, 102, 109].

#### Method to identify prescribing indicators

Methods used to identify indicators were reported in 66 (94.3%) of the studies. A total of 38 (54.3%) studies used one method to identify their prescribing indicators, with 28 (40.0%) using more than one method. Another 4 (5.7%) [17, 22, 79, 114] studies did not report a source of their indicators. Literature review was the most commonly method used, being used in 36 (51.4%) studies. Authors who provided additional detail described literature review processes as including searching for indicators from previously published tools and/or searching to identify new indicators from randomised controlled trials and observational studies.

Other reported sources of prescribing indicators included clinical experience (n = 16, 22.9%), selecting multiple previously published tools (n = 14, 20.0%) or a single tool (n = 7, 10.0%) (without mentioning literature review), guidelines (n = 9, 12.9%), textbooks (n = 6, 8.6%), older versions to be updated (n = 6, 8.6%), FDA black box warnings (n = 3, 4.3%), DDI references (n = 3, 4.3%), preliminary list of previous tool (n = 1, 1.4%) and medication databases (n = 1, 1.4%).

#### Validation method

The most commonly used method for validation of prescribing indicators was the Delphi method, [120] which was used during development of 34 (48.6%) tools (of these, 16/34 (47.1%) used a modified Delphi). The RAND/UCLA appropriateness method (RAM) [121] was used in development of 9 tools (12.9%) [10, 11, 43, 48, 64, 65, 72, 73, 86] (of these, 4/9 (44.4%) [11, 65, 73, 86] used a modified RAM). Of the remaining studies, 7 (10.0%) [69, 74, 104, 106, 108, 111, 112] used an expert panel, 2 (2.9%) [31, 75] used the Nominal Group Technique (NGT), 1 (1.4%) [109] used consensus among the research group without further description and 1 (1.4%) [107] used both Delphi and NGT. A total of 11 (15.7%) [39, 42, 49, 61, 62, 68, 79, 85, 99, 102, 115] studies used a non-specific consensus building approach, and 5 (7.1%) [17, 22, 81, 105, 114] did not report any validation of their prescribing indicators.

#### Type of prescribing indicators

A total of 67 (95.7%) studies developed prescribing process indicators. Numerous terms describing the prescribing processes of interest were used in the included studies. These included: hazardous, suboptimal, optimal, inappropriate, unsafe, high risk, omitted and unusual prescribing, prescribing appropriateness, drug-related problems (DRPs), adherence to management guidelines, PIM, high risk medication, DDI, drug disease interaction, inadequate monitoring and monitoring errors. The remaining 3 (4.3%) [38, 50, 107] studies developed prescribing outcome indicators to identify preventable drug related morbidity (PDRM) and preventable medication-related hospitalisations.

#### Categorising MH related prescribing indicators

From the 1386 extracted mental health related indicators, duplicates were removed and some indicators were split and re-categorised by the research team, which reduced the final number of the included indicators to 1106. These indicators were categorised into eight types of prescribing problems and into nine medication categories. The full list of mental health related indicators can be found in S2 File.

For prescribing problems, the highest number of indicators were categorised under ‘PIM: Considering Diagnoses or Conditions’ which contained 447 (40.4%) indicators. This was followed by ‘PIM: Independent of Diagnoses or Conditions’ (n = 269, 24.3%), ‘DDI’ (n = 153, 13.8%), ‘inappropriate duration’ and ‘inappropriate dose’ (n = 74 each, 6.7%). The categories containing the fewest number of indicators were ‘omission’ with only 8 (0.7%) indicators, along with ‘others’ (n = 28, 2.5%) and ‘monitoring’ indicators (n = 53, 4.8%).

Medications classed under the sedative, hypnotic and anxiolytics group were the most commonly reported in the developed tools with 317 indicators (28.7%). This was followed by antidepressants (n = 241, 21.8%), antipsychotics (n = 191, 17.3%) and mood stabilisers (n = 88, 8.0%). The remaining categories were anticholinergics (n = 56, 5.1%), anti-dementia (n = 49, 4.4%) and ADHD medications (n = 24, 2.2%). Fifteen indicators (1.4%) included psychotropics without specifying a class. Furthermore, 125 (11.3%) indicators included non-mental health medications with mental health conditions. These conditions included delirium, insomnia, depression, dementia, advanced dementia, palliative advanced dementia and non-palliative dementia. Table 4 summarises the number of prescribing indicators in each category.

**Table 4:** Numbers of prescribing indicators related to mental health in each prescribing problem and medication category.

Prescribing Problem	PIM Independent of Diagnoses or Conditions	PIM Considering Diagnoses or Conditions	DDI	Inappropriate Duration	Inappropriate Dose	Monitoring	Omission	Others	Total: n (%)
**Medication Category**									
Antipsychotics	45	85	13	19	18	7	0	4	**191 (17.3%)**
Antidepressants	42	102	67	9	9	0	4	8	**241 (21.8%)**
Sedative, hypnotics and anxiolytics	119	75	36	40	44	3	0	0	**317 (28.7%)**
Mood stabilisers	2	10	22	0	2	42	2	8	**88 (8.0%)**
Anti-dementia	27	13	7	0	0	0	2	0	**49 (4.4%)**
ADHD medications	8	13	1	0	1	1	0	0	**24 (2.2%)**
Anticholinergics	26	24	2	4	0	0	0	0	**56 (5.1%)**
Non-Specific Psychotropics	0	1	5	1	0	0	0	8	**15 (1.4%)**
Non-MH medication with MH condition	0	124	0	1	0	0	0	0	**125 (11.3%)**
**Total: n (%)**	**269 (24.3%)**	**447 (40.4%)**	**153 (13.8%)**	**74 (6.7%)**	**74 (6.7%)**	**53 (4.8%)**	**8 (0.7%)**	**28 (2.5%)**	**1106 (100%)**

ADHD: Attention deficit hyperactivity disorder. DDI: drug-drug interaction. MH: Mental Health. PIM: potentially inappropriate medication

### Stage 3: Selecting potential PSIs related to MH disorders and medications

From the 1106 identified MH related indicators, 245 were considered to meet our PSI definition following review as they described prescribing or drug monitoring practices that could be hazardous and may put patients at significant risk of harm. These potential PSIs were selected from 59 studies out of the 70 that included MH related indicators. Table 3 summarises the characteristics of the studies that potential PSIs related to MH were selected from (n = 59).

#### Categorising potential PSIs related to MH disorders and medications

Potential PSIs were categorised into eight types of prescribing problems. The highest number of indicators were categorised under ‘PIM: Considering Diagnoses or Conditions’ which contained 91 (37.1%) indicators. This was followed by ‘DDI’ (n = 66, 26.9%), ‘inappropriate dose’ (n = 24, 9.8%), ‘PIM: Independent of Diagnoses or Conditions’ (n = 20, 8.2%), ‘monitoring’ (n = 17, 6.9%), ‘inappropriate duration’ (n = 12, 4.9%), ‘Other’ (n = 10, 4.1%) and ‘Omission’ with only 5 (2.0%) indicators.

Potential PSI were also categorised into nine medication categories. Antidepressants were the most commonly selected with 85 (34.7%) potential PSIs. This was followed by sedative, hypnotic and anxiolytics (n = 50, 20.4%), antipsychotics (n = 38, 15.5%) and mood stabilisers (n = 33, 13.5%). The remaining were ADHD medications (n = 12, 4.9%), non-mental health medications with mental health conditions (n = 11, 4.5%), anticholinergics and anti-dementia (n = 7 each, 2.9%), and 2 indicators (0.8%) included psychotropics in general.

Table 5 summarises the number of potential PSIs in each category. Table 6 provides some examples of the selected potential PSIs. The full list can be found in S3 File.

**Table 5:** Numbers of potential prescribing safety indicators related to mental health in each prescribing problem and medication category.

Prescribing Problem	PIM Independent of Diagnoses or Conditions	PIM Considering Diagnoses or Conditions	DDI	Inappropriate Duration	Inappropriate Dose	Monitoring	Omission	Others	Total: n (%)
**Medication Category**									
Antipsychotics	2	19	4	3	3	6	0	1	**38 (15.5%)**
Antidepressants	7	37	31	3	3	0	2	2	**85 (34.7%)**
Sedative, hypnotics and anxiolytics	6	9	14	4	17	0	0	0	**50 (20.4%)**
Mood stabilisers	0	3	13	0	0	10	1	6	**33 (13.5%)**
Anti-dementia	0	3	2	0	0	0	2	0	**7 (2.9%)**
ADHD medications	4	5	1	0	1	1	0	0	**12 (4.9%)**
Anticholinergics	1	5	1	0	0	0	0	0	**7 (2.9%)**
Non-Specific Psychotropics	0	0	0	1	0	0	0	1	**2 (0.8%)**
Non-MH medication with MH condition	0	10	0	1	0	0	0	0	**11 (4.5%)**
**Total: n (%)**	**20 (8.2%)**	**91 (37.1%)**	**66 (26.9%)**	**12 (4.9%)**	**24 (9.8%)**	**17 (6.9%)**	**5 (2.0%)**	**10 (4.1%)**	**245 (100%)**

ADHD: Attention deficit hyperactivity disorder. DDI: drug-drug interaction. MH: Mental Health. PIM: potentially inappropriate medication

**Table 6:** Examples of the selected potential prescribing safety indicators.

Prescribing problem	Medication category	Example	Sources
**PIM: Independent of Diagnoses or Conditions**	***Antidepressants***	Prescribing tricyclic antidepressant to a patient aged ≥ 65 years	[32, 34, 38, 65]
**PIM: Considering diagnoses or conditions**	***Antipsychotics***	Prescribing antipsychotics other than quetiapine or clozapine to a patient aged ≥ 65 years with Parkinson’s disease	[25, 32, 48, 73, 92]
**DDI**	***Anticholinergics***	Prescribing two anticholinergics to a patient aged ≥ 65 years	[25, 32, 34, 48]
**Inappropriate Duration**	***Sedative*, *hypnotics and anxiolytics***	Prescribing Benzodiazepine for more than 1 month	[15, 99]
**Inappropriate dose**	***Antipsychotics***	Prescribing high dose antipsychotics (*total daily dose is above the maximum recommended by the British National Formulary)*	[22]
**Monitoring**	***Mood stabilisers***	Prescribing lithium without monitoring lithium plasma level every 3 months	[17, 61]
**Omission**	***Antidepressants***	Patients diagnosed with moderate/severe depressive symptoms lasting at least three months without prescribing antidepressant	[25]

DDI: drug-drug interaction. MH: Mental Health. PIM: potentially inappropriate medication.

## Discussion

To our knowledge, this is the first systemic review conducted to identify and screen all known published prescribing indicators and inappropriate prescribing tools in order to extract potential prescribing safety indicators (PSIs) related to populations with mental illness, and indeed any broader type of mental health related prescribing quality indicators. An earlier systematic review [20] published in 2014 was limited to inappropriate prescribing assessment tools, and another review by Song et al. [19] published in 2017 was limited to quality indicators and did not include most of the inappropriate prescribing tools which means that these reviews missed many studies which we found to contain potential PSIs and broader mental health related indicators, such as Gurerriro et al. in 2007 [50], Dreischulte et al. in 2012 [73] and Wessell et al. in 2010 [39].

We found 5 [22, 49, 69, 80, 86] studies specifically focused on developing/reporting prescribing indicators for populations with mental illness. However, two of these studies [80, 86] were exclusively for patients with dementia and one was for patients suffering with bipolar disorder [49]. Although 2 studies were found that involved development of prescribing indicators for a range of mental disorders and which contained some PSIs [22, 69], their main focus was not on safety and therefore they did not capture many hazardous prescribing issues, such as medication monitoring and omissions [22, 69].

It is clear from the findings that there has been an increase in the incidence of new explicit, patient-level data based suites of prescribing indicators being published for use across various patient populations over time. This might be a result of increased implementation of electronic health records worldwide [122] and the great improvements in the quality of these records which made operating electronic searches using prescribing indicators possible [123]. It also indicates an increasing emphasis on the quality and safety of healthcare, as noted in the wider literature [124]. A contributory factor to this rise might also be because indicators are used for audit and feedback purposes, which may be one of the more effective strategies to improve prescribing quality and quality of healthcare [125]. However, suites of prescribing indicators relevant to those with mental health illness have remained uncommon, and a specific suite of PSIs tailored to mental health illness and medications remains absent.

The methods used to identify indicators were reported in 94.3% of the studies reporting mental health related indicators, which is consistent with another systematic review that examined the development of general health care quality indicators using the Delphi method [126]. However, these methods varied significantly between the included studies, with some not reporting any sources for their indicators [17, 79, 80, 114], or using a single previously published study. In contrast, others conducted comprehensive systematic reviews of the relevant literature to identify previously published indicators or new potential indicators. Even though there is no agreed optimum method to identify/develop potential indicators reported in the literature, literature review was found to be the most commonly used method in this review and in a previous publication [126]. In addition, this method was also used by the Agency for Healthcare Research and Quality (AHRQ) to identify potential indicators [127]. Future research efforts should work towards building a consensus on the appropriate types and number of sources for the development of prescribing indicators, to guide researchers when developing new indicators.

Most studies reported a validation process with differences in approach and the depth of detail provided. The majority of studies used a consensus approach to validate their indicators. Each consensus method has its own advantages and disadvantages. However, there is a lack of standardisation in defining, using and reporting of consensus methods [128]. For example, some studies used modified Delphi and other used the RAM. However, the RAM can also be known as modified Delphi [121]. Therefore, it is important that studies report how the original method has been modified. Moreover, some studies did not specify which consensus method they used. In future it would be worthwhile to develop a method to assess the quality of implementation and reporting of consensus-based studies, and to develop a way to determine which method(s) might be most appropriate to apply for different prescribing indicators-based research projects based on their respective aims. A small number of studies did not report any process of validation for their indicators [17, 22, 67, 81, 99, 105, 108, 111, 112, 114, 115]. However, some of these studies did not aim to report the development of indicators such as the PINCER trial [17] which instead aimed to compare the effectiveness of an intervention and prescribing indicators were used as the outcome measure. Therefore, potential indicators retrieved from these studies require further validation.

This review has presented the number of all potential mental health related PSIs and broader mental health related prescribing indicators in each prescribing problem and in each therapeutic category. An expansive lists of different mental health related indicators has been identified. However, it is evident from the findings that some types of hazardous prescribing and therapeutic classes were under-represented in the published prescribing indicators and consequently in the selected potential PSIs. For instance, only 8 (0.7%) MH indicators and 5 (2.0%) potential PSIs for the category ‘omission’ were identified. Yet, it has been reported that omission is a predominant type of prescribing error in mental health hospitals [129, 130].

Likewise, monitoring indicators reported in the literature were mostly limited to mood stabilisers with few indicators for monitoring of antipsychotics, and no indicators for monitoring of antidepressants. As an example of potential PSIs that might have been missed include monitoring of liver function tests with agomelatine [131]. Additionally, more than two thirds of MH indicators and potential PSIs focused on antipsychotics, antidepressants and sedative-hypnotics. Conversely, other categories such as mood stabilisers, anti-dementia and ADHD medications, represented only 14.6% of the total number of indicators reported and 21.3% of the potential PSIs. This could suggest that these categories were marginalised in the literature and potential PSIs might have been missed.

Furthermore, the majority of the identified MH related indicators were developed for application to elderly populations, with a limited number of indicators designed for other populations such as younger people. Despite that evidence has shown that half of mental disorders start in childhood, and 75% by adolescence [132]. It is also found that in the UK about 1 in 10 children have a clinically diagnosable mental health problem [133]. Yet, 70% of those did not receive appropriate care [134]. In addition, no indicators have been reported for pregnant or breastfeeding women, despite the risk of some psychotropics in this group such as prescribing valproate in women of child bearing potential [135]. Consequently, it is important that future work takes the into consideration the unique characteristics of populations with mental illness, the different therapeutic classes of psychotropics and different prescribing problems when developing new suites of PSIs.

Based on our findings, none of the recently published sets of prescribing indicators were developed to be used specifically for mental health disorders and medications, and the PSIs group as a whole did not cover all known types of prescribing problems. The lists of potential PSIs and broader mental health related indicators identified in this review (S2 and S3 Files) have been identified from different types of studies with different purposes, settings and populations. In addition, the majority of these studies did not focus on patients with mental illness or clinical practice within specialist MH settings. Therefore, these indicators may not reflect all potential PSIs in metal health context. Hence, we have labelled these indicators as ‘potential’ and further development and validation may be recommended before they are applied into clinical practice locally. There is therefore a need to develop a new set of prescribing safety indicators specifically for application to patients with mental illness that addresses broad areas of potentially hazardous prescribing and drug monitoring in this population, and to undergo consensus-based refinement and validation with experts in mental health and medication management. As the identified indicator lists contain medications licensed in different countries across the globe, these might therefore be used as a foundation for other international research/clinical groups to achieve this goal by selecting relevant indicators for validation and feasibility processes for their specific countries and health settings, whether in specialist mental health hospitals/institutions or in primary care settings. In addition, because the database search strategy did not include any mental health terms, the list of included studies (Table 2) can be used as a source to identify indicators across a broad range of clinical conditions for populations across primary and secondary care.

In the UK, work is already underway across primary and secondary care to integrate PSIs into everyday clinical practice to identify patients at risk of harm, such as PNCER tool [136], investigating medication prescribing accuracy for critical error types (iMPACT) tool [137] and Salford medication safety (SMASH) dashboard [138, 139]. This has benefits for patient safety and this study is an important step towards achieving a similar aim for those with mental illness.

### Strength and limitations

Important strengths of this review include using seven databases for a comprehensive literature search, no limitation on languages to avoid language bias, no restriction on health settings or age group to capture the widest range of prescribing indicators and using a long-time frame of 28 years. In addition, our list of potential indicators was not restricted to practice in a specific country. A number of limitations were identified for this study. Despite efforts to enhance the comprehensiveness of the review by using a rigorous and thorough search strategy, it cannot be confirmed that the review located all relevant studies. The screening process was conducted by one author, which can increase the likelihood of discarding relevant articles [140]. No formal quality assessment too has been used to assess the quality of each included study. Not all of the identified MH related indicators were considered to have high clinical importance and may be likely to cause significant risk of harm, and they therefore might not be appropriate to assess the safety of prescribing. Accordingly, we attempted to select indicators, based on the clinical experience of the research team, which could be used to assess the safety of prescribing. Our selection process resulted in 245 indicators that were considered potentially appropriate to assess the safety of prescribing and drug monitoring. However, it is important to recognise inherent limitations in our process of PSI selection. Firstly, the selection of PSIs from published studies was carried out by two pharmacists using their clinical experience, knowledge of PSIs and the published literature. Secondly, some indicators that targeted the elderly or a specific medication were modified to cover all ages or a drug class, respectively if another indicator was present describing this association that the team felt was more appropriate, which we carried out based on the same sources of information. Together, these potential limitations in our selection process mean that we cannot therefore exclude the possibility that we may have overlooked or misinterpreted practice in both ours and other countries, and we therefore recommend that PSI suites using the findings of our review are further developed and validated by panels of health care professionals/experts with experience in the intended country of application in future using consensus building approaches.

## Conclusion

This is the first systematic review to identify a list of potential PSIs related to MH disorders and medications that may be used to assess the safety of prescribing. Examination of the included studies and the types of the identified potential PSIs extracted highlights the need for development of a suite of PSIs specific to patients with mental illness, and which covers all known areas of hazardous prescribing and drug monitoring in this patient group. The findings of this review should be used as a foundation for others across the globe to develop, validate and apply their own PSIs for patients with mental illness across different settings to monitor and improve patient care.

## Supporting information

S1 File Search strategy. (PDF)

S2 File List of prescribing indicators related to mental health medications and conditions. (PDF)

S3 File List of potential Prescribing Safety Indicators (PSIs) related to mental health medications and conditions. (PDF)

S4 File PRISMA checklist. (PDF)

## References

[1] Vos T, Barber RM, Bell B, Bertozzi-Villa A, Biryukov S, Bolliger I, et al Global, regional, and national incidence, prevalence, and years lived with disability for 301 acute and chronic diseases and injuries in 188 countries, 1990–2013: a systematic analysis for the Global Burden of Disease Study 2013. The Lancet. 2015;386(9995):743. 10.1016/S0140-6736(15)60692-4 PMC4561509 26063472

[2] Steel Z, Marnane C, Iranpour C, Chey T, Jackson JW, Patel V, et al The global prevalence of common mental disorders: a systematic review and meta-analysis 1980–2013. International journal of epidemiology. 2014;43(2):476–93. 10.1093/ije/dyu038 24648481 PMC3997379

[3] British Medical Association. Breaking down barriers–the challenge of improving mental health outcomes: British Medical Association; 2017 [cited 2018 July 4]. Available from: https://www.bma.org.uk/-/media/files/pdfs/collective%20voice/policy%20research/public%20and%20population%20health/mental%20health/breaking-down-barriers-mental-health-briefing-apr2017.pdf?la=en.

[4] Mitchell AJ, Lord O, Malone D. Differences in the prescribing of medication for physical disorders in individuals with v. without mental illness: meta-analysis. The British Journal of Psychiatry. 2012;201(6):435–43. 10.1192/bjp.bp.111.094532 23209089

[5] McManus S, Bebbington P, Jenkins R, T B. Mental health and wellbeing in England: Adult Psychiatric Morbidity Survey 2014 Leeds: NHS Digital; 2014 [cited 2018 April 3]. Available from: https://www.gov.uk/government/uploads/system/uploads/attachment_data/file/556596/apms-2014-full-rpt.pdf

[6] Mann K, Rothschild JM, Keohane CA, Chu JA, Bates DW. Adverse drug events and medication errors in psychiatry: methodological issues regarding identification and classification. The World Journal of Biological Psychiatry. 2008;9(1):24–33. 10.1080/15622970601178056 17853253

[7] Soerensen AL, Nielsen LP, Poulsen BK, Lisby M, Mainz J. Potentially inappropriate prescriptions in patients admitted to a psychiatric hospital. Nordic Journal of Psychiatry. 2016;70(5):365–73. 10.3109/08039488.2015.1127996 26824679

[8] Alshehri GH, Keers RN, Ashcroft DM. Frequency and nature of medication errors and adverse drug events in mental health hospitals: A systematic review. Drug Safety. 2017;40(10):871–86. 10.1007/s40264-017-0557-7 28776179

[9] Guthrie B, Yu N, Murphy D, Donnan PT, Dreischulte T. Health Services and Delivery Research. Measuring prevalence, reliability and variation in high-risk prescribing in general practice using multilevel modelling of observational data in a population database. Southampton (UK): NIHR Journals Library; 2015. 26539601

[10] Spencer R, Bell B, Avery AJ, Gookey G, Campbell SM, Royal College of General P. Identification of an updated set of prescribing—safety indicators for GPs. Br J Gen Pract. 2014;64(621):e181–90. 10.3399/bjgp14X677806 24686882 PMC3964450

[11] Guthrie B, McCowan C, Davey P, Simpson CR, Dreischulte T, Barnett K. High risk prescribing in primary care patients particularly vulnerable to adverse drug events: Cross sectional population database analysis in Scottish general practice. BMJ: British Medical Journal. 2011;342(7812):1–12. 10.1136/bmj.d3514 21693525

[12] Stocks SJ, Kontopantelis E, Akbarov A, Rodgers S, Avery AJ, Ashcroft DM. Examining variations in prescribing safety in UK general practice: cross sectional study using the Clinical Practice Research Datalink. BMJ. 2015;351. 10.1136/bmj.h5501 PMC4632209 26537416

[13] Avery AJ, Rodgers S, Cantrill JA, Armstrong S, Cresswell K, Eden M, et al A pharmacist-led information technology intervention for medication errors (PINCER): a multicentre, cluster randomised, controlled trial and cost-effectiveness analysis. The Lancet. 2012;379(9823):1310–9. 10.1016/S0140-6736(11)61817-5 PMC3328846 22357106

[14] Pontefract SK, Hodson J, Slee A, Shah S, Girling AJ, Williams R, et al Impact of a commercial order entry system on prescribing errors amenable to computerised decision support in the hospital setting: a prospective pre-post study. BMJ Quality & Safety. 2018;27(9):725–36. 10.1136/bmjqs-2017-007135 29572298 PMC6109251

[15] Thomas SK, McDowell SE, Hodson J, Nwulu U, Howard RL, Avery AJ, et al Developing consensus on hospital prescribing indicators of potential harms amenable to decision support. Br J Clin Pharmacol. 2013;76(5):797–809. 10.1111/bcp.12087 23362926 PMC3853538

[16] Department of Health and Social Care. Medicine safety: indicators for safer prescribing 2018 [cited 2018 July 2]. Available from: https://www.gov.uk/government/publications/medicine-safety-indicators-for-safer-prescribing.

[17] Avery AJ, Rodgers S, Cantrill JA, Armstrong S, Elliott R, Howard R, et al Protocol for the PINCER trial: a cluster randomised trial comparing the effectiveness of a pharmacist-led IT-based intervention with simple feedback in reducing rates of clinically important errors in medicines management in general practices. Trials. 2009;10:28 10.1186/1745-6215-10-28 19409095 PMC2685134

[18] NICE. Medicines optimisation: the safe and effective use of medicines to enable the best possible outcomes: NICE guideline.(NG5): The National Institute for Health and Care Excellence; 2015 [cited 2018 April 4]. Available from: https://www.nice.org.uk/guidance/ng5/resources/medicines-optimisation-the-safe-and-effective-use-of-medicines-to-enable-the-best-possible-outcomes-pdf-51041805253. 26180890

[19] Song J, Zhang L, Li Y, Zeng L, Hu D, Liang Y, et al Indicators for assessing quality of drug use: a systematic literature review. Journal of evidence-based medicine. 2017;10(3):222–32. 10.1111/jebm.12244 28276644

[20] Kaufmann CP, Tremp R, Hersberger KE, Lampert ML. Inappropriate prescribing: a systematic overview of published assessment tools. European journal of clinical pharmacology. 2014;70(1):1–11. 10.1007/s00228-013-1575-8 24019054

[21] Avery AJ, Dex GM, Mulvaney C, Serumaga B, Spencer R, Lester HE, et al Development of prescribing-safety indicators for GPs using the RAND Appropriateness Method. Br J Gen Pract. 2011;61(589):e526–e36. 10.3399/bjgp11X588501 21801572 PMC3145537

[22] Paton C, Lelliott P. The use of prescribing indicators to measure the quality of care in psychiatric inpatients. Quality and Safety in Health Care. 2004;13(1):40–5. 10.1136/qshc.2003.006338 14757798 PMC1758059

[23] Basger BJ, Chen TF, Moles RJ. Inappropriate medication use and prescribing indicators in elderly Australians: Development of a prescribing indicators tool. Drugs & Aging. 2008;25(9):777–93. 10.2165/00002512-200825090-00004. 18729548

[24] Lavan AH, Gallagher PF, O’Mahony D. Methods to reduce prescribing errors in elderly patients with multimorbidity. Clinical interventions in aging. 2016;11:857 10.2147/CIA.S80280 27382268 PMC4922820

[25] O'Mahony D, O'Sullivan D, Byrne S, O'Connor MN, Ryan C, Gallagher P. STOPP/START criteria for potentially inappropriate prescribing in older people: version 2 …screening tool of older people’s prescriptions … screening tool to alert to right treatment. Age & Ageing. 2015;44(2):213–8. ageing/afu145. . Language: English. Entry Date: 20150309. Revision Date: 20160229. Publication Type: Journal Article.25324330

[26] Hanlon JT, Schmader KE, Samsa GP, Weinberger M, Uttech KM, Lewis IK, et al A method for assessing drug therapy appropriateness. Journal of clinical epidemiology. 1992;45(10):1045–51. 1474400 10.1016/0895-4356(92)90144-c

[27] Campbell SM, Cantrill JA, Roberts D. Prescribing indicators for UK general practice: Delphi consultation study. BMJ. 2000;321(7258):425 10.1136/bmj.321.7258.425 10938052 PMC27458

[28] Tully MP, Javed N, Cantrill JA. Development and face validity of explicit indicators of appropriateness of long term prescribing. Pharmacy World and Science. 2005;27(5):407–13. 10.1007/s11096-005-0340-1 16341749

[29] Smits KP, Sidorenkov G, Kleefstra N, Bouma M, Meulepas M, Voorham J, et al Development and validation of prescribing quality indicators for patients with type 2 diabetes. International journal of clinical practice. 2017;71(1). 10.1111/ijcp.12922 27981681

[30] Wenger NS, Shekelle PG. Assessing care of vulnerable elders: ACOVE project overview. Annals of Internal Medicine. 2001;135(8_Part_2):642–6. 11601946 10.7326/0003-4819-135-8_part_2-200110161-00002

[31] Maio V, Canale SD, Abouzaid S. Using explicit criteria to evaluate the quality of prescribing in elderly Italian outpatients: A cohort study. Journal of Clinical Pharmacy and Therapeutics. 2010;35(2):219–29. 10.1111/j.1365-2710.2009.01094.x 20456742

[32] Khodyakov D, Ochoa A, Olivieri-Mui BL, Bouwmeester C, Zarowitz BJ, Patel M, et al Screening Tool of Older Person's Prescriptions/Screening Tools to Alert Doctors to Right Treatment Medication Criteria Modified for US Nursing Home Setting. Journal of the American Geriatrics Society. 2017;65(3):586–91. 10.1111/jgs.14689 WOS:000397794300024. 28008599 PMC5370573

[33] Pazan F, Weiss C, Wehling M. The FORTA (Fit fOR The Aged) List 2015: Update of a Validated Clinical Tool for Improved Pharmacotherapy in the Elderly. Drugs and Aging. 2016;33(6):447–9. 10.1007/s40266-016-0371-4 27166962

[34] By the American Geriatrics Society Beers Criteria Update Expert Panel. American Geriatrics Society 2015 Updated Beers Criteria for Potentially Inappropriate Medication Use in Older Adults. Journal of the American Geriatrics Society. 2015;63(11):2227–46. 10.1111/jgs.13702 26446832

[35] Royal Pharmaceutical Society. MedicinesComplete: Royal Pharmaceutical Society; 2017 [cited 2018 July 31]. Available from: https://www.medicinescomplete.com/.

[36] WHO. ICD -10: The international statistical classification of diseases and relate health problems (Version:2016) 2016 [cited 2018 April 4]. Available from: http://apps.who.int/classifications/icd10/browse/2016/en

[37] American Psychiatric Association. Diagnostic and Statistical Manual of Mental Disorders (DSM-5). 5th ed Arlington, VA: American Psychiatric Association; 2013.

[38] Mackinnon NJ, Hepler CD. Preventable drug-related morbidity in older adults 1. Indicator development (Part 1 of a 2-part series). Journal of Managed Care Pharmacy. 2002;8(5):365–71. 10.18553/jmcp.2002.8.5.365 14613403

[39] Wessell AM, Litvin C, Jenkins RG, Nietert PJ, Nemeth LS, Ornstein SM. Medication prescribing and monitoring errors in primary care: a report from the Practice Partner Research Network. Quality & Safety in Health Care. 2010;19(5). 10.1136/qshc.2009.034678 WOS:000285032700041. 20413615

[40] Fick DM, Cooper JW, Wade WE, Waller JL, Beers MH, et al Updating the beers criteria for potentially inappropriate medication use in older adults—Results of a US consensus panel of experts. Archives of Internal Medicine. 2003;163(22):2716–24. 10.1001/archinte.163.22.2716 14662625

[41] Rancourt C, Moisan J, Baillargeon L, Verreault R, Laurin D, Gregoire JP. Potentially inappropriate prescriptions for older patients in long-term care. BMC geriatrics. 2004;4:9 10.1186/1471-2318-4-9 15488143 PMC529256

[42] Van der Linden L, Decoutere L, Flamaing J, Spriet I, Willems L, Milisen K, et al Development and validation of the RASP list (Rationalization of Home Medication by an Adjusted STOPP list in Older Patients): A novel tool in the management of geriatric polypharmacy. European Geriatric Medicine. 2014;5(3):175–80. 10.1016/j.eurger.2013.12.005 WOS:000338387500007.

[43] Castillo-Paramo A, Pardo-Lopo R, Gomez-Serranillos IR, Verdejo A, Figueiras A, Claveria A. [Assessment of the appropriateness of STOPP/START criteria in primary health care in Spain by the RAND method]. SEMERGEN—Medicina de Familia. 2013;39(8):413–20. Spanish. 10.1016/j.semerg.2013.01.017 24230489

[44] Onder G, Bonassi S, Abbatecola AM, Folino-Gallo P, Lapi F, Marchionni N, et al High Prevalence of Poor Quality Drug Prescribing in Older Individuals: A Nationwide Report From the Italian Medicines Agency (AIFA). Journals of Gerontology Series a-Biological Sciences and Medical Sciences. 2014;69(4):430–7. 10.1093/gerona/glt118 WOS:000333385500007. 23913935

[45] Holt S, Schmiedl S, Thurmann PA. Potentially inappropriate medications in the elderly: The PRISCUS list. Deutsches Arzteblatt International. 2010;107(31–32):543–51. 10.3238/arztebl.2010.0543 20827352 PMC2933536

[46] Renom-Guiteras A, Meyer G, Thürmann P. The EU(7)-PIM list: a list of potentially inappropriate medications for older people consented by experts from seven European countries. European Journal of Clinical Pharmacology. 2015;71(7):861–75. 10.1007/s00228-015-1860-9 . Language: English. Entry Date: 20150624. Revision Date: 20160630. Publication Type: Journal Article. 25967540 PMC4464049

[47] Fialova D, Topinkova E, Ballokova A, Matejovska-Kubesova H. [2012 CZ expert consensus for potentially inappropriate medication use in old age: Appropriate choice of drugs and drug dosing in geriatric patients (Section I.), drug-disease interactions in the old age (Section II.)]. Klinicka Farmakologie a Farmacie. 2013;27(1):18–28. Czech.

[48] Tommelein E, Petrovic M, Somers A, Mehuys E, van der Cammen T, Boussery K. Older patients' prescriptions screening in the community pharmacy: development of the Ghent Older People's Prescriptions community Pharmacy Screening (GheOPA(3)S) tool. Journal of Public Health. 2016;38(2):E158–E70. 10.1093/pubmed/fdv090 WOS:000383511000021. 26175537

[49] Al-Taweel DM, Alsuwaidan M. A medication assessment tool to evaluate prescribers' adherence to evidence-based guidelines in bipolar disorder. International Journal of Clinical Pharmacy. 2017;39(4):897–905. 10.1007/s11096-017-0498-3 28653259

[50] Guerreiro MP, Cantrill JA, Martins AP. Preventable drug-related morbidity: Determining valid indicators for primary care in Portugal. Acta Medica Portuguesa. 2007;20(2):107–30. 17868517

[51] NICE. NICE guidance: National Institute for Health and Care Excellence; [cited 2018 December 3]. Available from: https://www.nice.org.uk.

[52] Taylor DM, Barnes TRE, Young AH. The Maudsley Prescribing Guidelines in Psychiatry. Hoboken, NJ: Wiley Blackwell; 2018.

[53] Bazire S. Psychotropic drug directory 2018: the professionals' pocket handbook and aide memoire: Lloyd-Reinhold; 2018.

[54] Beers MH, Ouslander JG, Rollingher I, Reuben DB, Brooks J, Beck JC. Explicit criteria for determining inappropriate medication use in nursing home residents. Archives of Internal Medicine. 1991;151(9):1825–32. 10.1001/archinte.1991.00400090107019 1888249

[55] Beers MH. Explicit criteria for determining potentially inappropriate medication use by the elderly—An update. Archives of Internal Medicine. 1997;157(14):1531–6. 10.1001/archinte.157.14.1531 WOS:A1997XL72900002. 9236554

[56] American Geriatrics Society Beers Criteria Update Expert Panel. American Geriatrics Society updated Beers Criteria for potentially inappropriate medication use in older adults. Journal of the American Geriatrics Society. 2012;60(4):616–31. 10.1111/j.1532-5415.2012.03923.x 22376048 PMC3571677

[57] Morris CJ, Cantrill JA, Hepler CD, Noyce PR. Preventing drug-related morbidity—determining valid indicators. International Journal for Quality in Health Care. 2002 14(3):183–98. WOS:000176249200007. 10.1093/oxfordjournals.intqhc.a002610 12108529

[58] Kuhn-Thiel AM, Weis C, Wehling M, members Faep. Consensus validation of the FORTA (Fit fOR The Aged) List: a clinical tool for increasing the appropriateness of pharmacotherapy in the elderly. Drugs & aging. 2014;31(2):131–40. 10.1007/s40266-013-0146-0. 24353033 PMC3907693

[59] Gallagher P, Ryan C, Byrne S, Kennedy J, O'Mahony D. STOPP (Screening Tool of Older Person's Prescriptions) and START (Screening Tool to Alert doctors to Right Treatment). Consensus validation. International journal of clinical pharmacology and therapeutics. 2008;46(2):72–83. 18218287 10.5414/cpp46072

[60] Kim DS, Heo SI, Lee SH. Development of a list of potentially inappropriate drugs for the korean elderly using the delphi method. Healthcare informatics research. 2010;16(4):231–52. 10.4258/hir.2010.16.4.231 21818443 PMC3092136

[61] Alldred DP, Standage C, Zermansky AG, Jesson B, Raynor DK, et al Development and validation of criteria to identify medication-monitoring errors in care home residents. International Journal of Pharmacy Practice. 2008;16(5):317–23.

[62] Barnett KN, Bennie M, Treweek S, Robertson C, Petrie DJ, Ritchie LD, et al Effective Feedback to Improve Primary Care Prescribing Safety (EFIPPS) a pragmatic three-arm cluster randomised trial: designing the intervention (ClinicalTrials.gov registration NCT01602705). Implementation science: IS. 2014;9:133 10.1186/s13012-014-0133-9 25304255 PMC4201916

[63] Barry E, O'Brien K, Moriarty F, Cooper J, Redmond P, Hughes CM, et al PIPc study: development of indicators of potentially inappropriate prescribing in children (PIPc) in primary care using a modified Delphi technique. BMJ open. 2016;6(9):e012079 10.1136/bmjopen-2016-012079 27601499 PMC5020844

[64] Basger BJ, Chen TF, Moles RJ. Validation of prescribing appropriateness criteria for older Australians using the RAND/UCLA appropriateness method. BMJ Open. 2012;2(5):e001431 10.1136/bmjopen-2012-001431 22983875 PMC3467596

[65] Caughey GE, Kalisch Ellett LM, Wong TY. Development of evidence-based Australian medication-related indicators of potentially preventable hospitalisations: a modified RAND appropriateness method. BMJ open. 2014;4(4):e004625 10.1136/bmjopen-2013-004625 24776711 PMC4010844

[66] Chang C-B, Yang S-Y, Lai H-Y, Wu R-S, Liu H-C, Hsu H-Y, et al Using published criteria to develop a list of potentially inappropriate medications for elderly patients in Taiwan. Pharmacoepidemiology and drug safety. 2012;21(12):1269–79. 10.1002/pds.3274 22517563

[67] Chen YF, Avery AJ, Neil KE, Johnson C, Stockley IH, et al Incidence and possible causes of prescribing potentially hazardous/contraindicated drug combinations in general practice. Drug Safety. 2005;28(1):67–80. 10.2165/00002018-200528010-00005 15649106

[68] Clyne B, Bradley MC, Hughes CM, Clear D, McDonnell R, Williams D, et al Addressing potentially inappropriate prescribing in older patients: development and pilot study of an intervention in primary care (the OPTI-SCRIPT study). BMC health services research. 2013;13:307 10.1186/1472-6963-13-307 23941110 PMC3751793

[69] Constantine RJ, McPherson MA, Jones ME, Tandon R, Becker ER. Improving psychotherapeutic medication prescribing in Florida: implementation of the Florida Medicaid Drug Therapy Management Program (MDTMP). Community mental health journal. 2013;49(1):33–44. 10.1007/s10597-012-9497-y 22383046

[70] Cooper JA, Ryan C, Smith SM, Wallace E, Bennett K, Cahir C, et al The development of the PROMPT (PRescribing Optimally in Middle-aged People's Treatments) criteria. BMC health services research. 2014;14:484 10.1186/s12913-014-0484-6 25410615 PMC4229620

[71] Desnoyer A, Blanc AL, Pourcher V, Besson M, Fonzo-Christe C, Desmeules J, et al PIM-Check: Development of an international prescription-screening checklist designed by a Delphi method for internal medicine patients. BMJ Open. 2017;7(7):e016070 10.1136/bmjopen-2017-016070 28760793 PMC5642656

[72] Desrochers JF, Lemieux JP, Morin-Bélanger C, Paradis FS, Lord A, Bell R, et al Development and Validation of the PAIR (Pharmacotherapy Assessment in Chronic Renal Disease) Criteria to Assess Medication Safety and Use Issues in Patients With CKD. American Journal of Kidney Diseases. 2011;58(4):527–35. 10.1053/j.ajkd.2011.04.020 . Language: English. Entry Date: 20120323. Revision Date: 20150712. Publication Type: Journal Article. 21778005

[73] Dreischulte T, Grant AM, McCowan C, McAnaw JJ, Guthrie B. Quality and safety of medication use in primary care: consensus validation of a new set of explicit medication assessment criteria and prioritisation of topics for improvement. BMC clinical pharmacology. 2012;12:5 10.1186/1472-6904-12-5 22316181 PMC3296596

[74] Elliott RA, Woodward MC, Oborne CA. Indicators of prescribing quality for elderly hospital inpatients. Australian Journal of Hospital Pharmacy. 2001;31(1):19–25. 10.1046/j.1445-5994.2001.00139.x 11767867

[75] Fernandez Urrusuno R, Montero Balosa MC, Perez Perez P, Pascual de la Pisa B. Compliance with quality prescribing indicators in terms of their relationship to financial incentives. European journal of clinical pharmacology. 2013;69(10):1845–53. 10.1007/s00228-013-1542-4 23743780

[76] Fox A, Pontefract S, Brown D, Portlock J, Coleman J. Developing consensus on hospital prescribing indicators of potential harm for infants and children. British journal of clinical pharmacology. 2016;82(2):451–60. 10.1111/bcp.12954 27038331 PMC4972161

[77] Galán Retamal C, Garrido Fernández R, Fernández Espínola S, Ruiz Serrato A, García Ordóñez MA, Padilla Marín V. [Prevalence of potentially inappropriate medication in hospitalized elderly patients by using explicit criteria]. Farmacia Hospitalaria (1130–6343). 2014;38(4):305–16. Spanish. 10.7399/fh.2014.38.4.1148 25137164

[78] Hanora Lavan A, Gallagher P, Parsons C, O'Mahony D. STOPPFrail (Screening Tool of Older Persons Prescriptions in Frail adults with limited life expectancy): consensus validation. Age & Ageing. 2017;46(4):600–7. 10.1093/ageing/afx005 28119312

[79] Harper MB, Longhurst CA, McGuire TL, Tarrago R, Desai BR, Patterson A, et al Core Drug-Drug Interaction Alerts for Inclusion in Pediatric Electronic Health Records With Computerized Prescriber Order Entry. Journal of Patient Safety. 2014;10(1):59–63. 10.1097/PTS.0000000000000050 WOS:000335830300008. 24522227

[80] Holmes HM, Sachs GA, Shega JW, Hougham GW, Hayley DC, Dale W. Integrating palliative medicine into the care of persons with advanced dementia: Identifying appropriate medication use. Journal of the American Geriatrics Society. 2008;56(7):1306–11. 10.1111/j.1532-5415.2008.01741.x 18482301

[81] Hurley JS, Roberts M, Solberg LI, Gunter MJ, Nelson WW, Young L, et al Laboratory safety monitoring of chronic medications in ambulatory care settings. Journal of general internal medicine. 2005;20(4):331–3. 10.1111/j.1525-1497.2005.40182.x 15857489 PMC1490088

[82] Kim D-S, Je NK, Kim GJ, Kang H, Kim YJ, Lee S. Therapeutic duplicate prescribing in Korean ambulatory care settings using the National Health Insurance claim data. International Journal of Clinical Pharmacy. 2015;37(1):76–85. 10.1007/s11096-014-0042-7 . Language: English. Entry Date: 20150923. Revision Date: 20171105. Publication Type: journal article. 25428447

[83] Kim SO, Jang S, Kim CM, Kim YR, Sohn HS. Consensus Validated List of Potentially Inappropriate Medication for the Elderly and Their Prevalence in South Korea. International Journal of Gerontology. 2015;9(3):136–41. 10.1016/j.ijge.2015.05.013 WOS:000363360600002.

[84] Kim MY, Etherton-Beer C, Kim CB, Yoon JL, Ga H, Kim HC, et al Development of a Consensus List of Potentially Inappropriate Medications for Korean Older Adults. Annals of Geriatric Medicine and Research. 2018;22(3):121–9. 10.4235/agmr.2018.22.3.121 PMC7387587 32743261

[85] Kojima T, Mizukami K, Tomita N, Arai H, Ohrui T, Eto M, et al Screening Tool for Older Persons' Appropriate Prescriptions for Japanese: Report of the Japan Geriatrics Society Working Group on "Guidelines for medical treatment and its safety in the elderly". Geriatrics & gerontology international. 2016;16(9):983–1001. 10.1111/ggi.12890. 27594406

[86] Kröger E, Wilchesky M, Marcotte M, Voyer P, Morin M, Champoux N, et al Medication Use Among Nursing Home Residents With Severe Dementia: Identifying Categories of Appropriateness and Elements of a Successful Intervention. Journal of the American Medical Directors Association. 2015;16(7):629.e1–.e17. 10.1016/j.jamda.2015.04.002 . Language: English. Entry Date: 20150625. Revision Date: 20150923. Publication Type: Journal Article. 25979776

[87] Laroche M-L, Charmes J-P, Merle L. Potentially inappropriate medications in the elderly: a French consensus panel list. European journal of clinical pharmacology. 2007;63(8):725–31. 10.1007/s00228-007-0324-2 17554532

[88] Lindblad CI, Hanlon JT, Gross CR, Sloane RJ, Pieper CF, Hajjar ER, et al Clinically important drug-disease interactions and their prevalence in older adults. Clinical Therapeutics. 2006;28(8):1133–43. 10.1016/j.clinthera.2006.08.006 WOS:000240521000006. 16982290

[89] Malone DC, Abarca J, Hansten PD, Grizzle AJ, Armstrong EP, Van Bergen RC, et al Identification of serious drug-drug interactions: results of the partnership to prevent drug-drug interactions. Journal of the American Pharmacists Association: JAPhA. 2004;44(2):142–51. 15098848 10.1331/154434504773062591

[90] Mann E, Bohmdorfer B, Fruhwald T, Roller-Wirnsberger RE, Dovjak P, Duckelmann-Hofer C, et al Potentially inappropriate medication in geriatric patients: the Austrian consensus panel list. Wiener Klinische Wochenschrift. 2012;124(5–6):160–9. 10.1007/s00508-011-0061-5 WOS:000302374700005. 22134410

[91] Marzi MM, Pires MS, Quaglia NB. Ingredientes Farmaceuticos Activos Potencialmente Inapropiados en Adultos Mayores: Lista IFAsPIAM: Panel de Consenso Argentino. Value in Health Regional Issues. 2018;17:38–55 Spanish. 10.1016/j.vhri.2017.10.002 29679895

[92] Mast R, Ahmad A, Hoogenboom SC, Cambach W, Elders PJ, Nijpels G, et al Amsterdam tool for clinical medication review: development and testing of a comprehensive tool for pharmacists and general practitioners. BMC research notes. 2015;8:642 10.1186/s13104-015-1566-1 26536861 PMC4632353

[93] McLeod PJ, Huang AR, Tamblyn RM, Gayton DC. Defining inappropriate practices in prescribing for elderly people: A national consensus panel. Canadian Medical Association Journal. 1997;156(3):385–91. WOS:A1997WF68200026. 9033421 PMC1226961

[94] Morris CJ, Cantrill JA. Preventing drug-related morbidity—the development of quality indicators. Journal of Clinical Pharmacy & Therapeutics. 2003;28(4):295–305. . Language: English. Entry Date: 20070101. Revision Date: 20150711. Publication Type: Journal Article. 12911682 10.1046/j.1365-2710.2003.00496.x

[95] Nyborg G, Straand J, Klovning A, Brekke M. The Norwegian General Practice—Nursing Home criteria (NORGEP-NH) for potentially inappropriate medication use: A web-based Delphi study. Scandinavian journal of primary health care. 2015;33(2):134–41. 10.3109/02813432.2015.1041833 26100966 PMC4834501

[96] Rognstad S, Brekke M, Fetveit A, Spigset O, Wyller TB, Straand J. The Norwegian General Practice (NORGEP) criteria for assessing potentially inappropriate prescriptions to elderly patients. A modified Delphi study. Scandinavian Journal of Primary Health Care. 2009;27(3):153–9. 10.1080/02813430902992215 . Language: English. Entry Date: 20091023. Revision Date: 20150711. Publication Type: Journal Article. 19462339 PMC3413187

[97] Oborne CA, Batty GM, Maskrey V, Swift CG, Jackson SHD. Development of prescribing indicators for elderly medical inpatients. British Journal of Clinical Pharmacology. 1997;43(1):91–7. 10.1111/j.1365-2125.1997.tb00038.x 9056058

[98] Oborne CA, Hooper R, Swift CG, Jackson SHD. Explicit, evidence-based criteria to assess the quality of prescribing to elderly nursing home residents. Age and Ageing. 2003;32(1):102–8. WOS:000180741000019. 10.1093/ageing/32.1.102 12540356

[99] Okechukwu I, Bennett K, Feely J. General practitioners' ranking of evidence-based prescribing quality indicators: a comparative study with a prescription database. British Journal of Clinical Pharmacology. 2006;62(2):218–24. 10.1111/j.1365-2125.2006.02621.x WOS:000239002900012. 16842397 PMC1885100

[100] Onder G, Landi F, Fusco D, Corsonello A, Lattanzio F, et al Recommendations to Prescribe in Complex Older Adults: Results of the CRIteria to Assess Appropriate Medication Use Among Elderly Complex Patients (CRIME) Project. Drugs & Aging. 2014;31(1):33–45. 24234805 10.1007/s40266-013-0134-4

[101] Pazan F, Weiss C, Wehling M, Forta. The EURO-FORTA (Fit fOR The Aged) List: International Consensus Validation of a Clinical Tool for Improved Drug Treatment in Older People. Drugs & Aging. 2018;35(1):61–71. 10.1007/s40266-017-0514-2 29335932

[102] Phansalkar S, Desai AA, Bell D, Yoshida E, Doole J, Czochanski M, et al High-priority drug–drug interactions for use in electronic health records. Journal of the American Medical Informatics Association. 2012;19(5):735–43. 10.1136/amiajnl-2011-000612 22539083 PMC3422823

[103] Prot-Labarthe S, Weil T, Angoulvant F, Boulkedid R, Alberti C, Bourdon O. POPI (Pediatrics: Omission of Prescriptions and Inappropriate Prescriptions): Development of a Tool to Identify Inappropriate Prescribing. Plos One. 2014;9(6). 10.1371/journal.pone.0101171 WOS:000338506400091. 24978045 PMC4076280

[104] Quintens C, De Rijdt T, Van Nieuwenhuyse T, Simoens S, Peetermans WE, Van den Bosch B, et al Development and implementation of "Check of Medication Appropriateness" (CMA): advanced pharmacotherapy-related clinical rules to support medication surveillance. Bmc Medical Informatics and Decision Making. 2019;19 10.1186/s12911-019-0748-5 30744674 PMC6371500

[105] Raebel MA, Chester EA, Newsom EE, Lyons EE, Kelleher JA, Long C, et al Randomized trial to improve laboratory safety monitoring of ongoing drug therapy in ambulatory patients. Pharmacotherapy: The Journal of Human Pharmacology and Drug Therapy. 2006;26(5):619–26. 10.1592/phco.26.5.619 16637791

[106] Raebel MA, Charles J, Dugan J, Carroll NM, Korner EJ, Brand DW, et al Randomized trial to improve prescribing safety in ambulatory elderly patients. Journal of the American Geriatrics Society. 2007;55(7):977–85. 10.1111/j.1532-5415.2007.01202.x WOS:000247607100001. 17608868

[107] Robertson HA, MacKinnon NJ. Development of a list of consensus-approved clinical indicators of preventable drug-related morbidity in older adults. Clinical Therapeutics. 2002;24(10):1595–613. 10.1016/s0149-2918(02)80063-7 WOS:000179310300008. 12462289

[108] Ruths S, Straand J, Nygaard HA. Multidisciplinary medication review in nursing home residents: what are the most significant drug-related problems? The Bergen District Nursing Home (BEDNURS) study. Quality & Safety in Health Care. 2003;12(3):176–80. . Language: English. Entry Date: 20040109. Revision Date: 20150711. Publication Type: Journal Article. 12792006 10.1136/qhc.12.3.176 PMC1743717

[109] Saverno KR, Hines LE, Warholak TL, Grizzle AJ, Babits L, Clark C, et al Ability of pharmacy clinical decision-support software to alert users about clinically important drug-drug interactions. Journal of the American Medical Informatics Association: JAMIA. 2011;18(1):32–7. 10.1136/jamia.2010.007609 21131607 PMC3005877

[110] Smits KP, Sidorenkov G, Bilo HJ, Bouma M, van Ittersum FJ, Voorham J, et al Development and initial validation of prescribing quality indicators for patients with chronic kidney disease. Nephrology Dialysis Transplantation. 2016;31(11):1876–86. 10.1093/ndt/gfv420 26743176

[111] Solberg LI, Hurley JS, Roberts MH, Nelson WW, Young LR, et al Measuring patient safety in ambulatory care: Potential for identifying medical group drug-drug interaction rates using claims data. The American Journal of Managed Care. 2004;10(11, Part 1):753–9. 15623265

[112] Tamblyn RM, McLeod PJ, Abrahamowicz M, Monette J, Gayton DC, Berkson L, et al Questionable prescribing for elderly patients in Quebec. CMAJ: Canadian Medical Association journal = journal de l'Association medicale canadienne. 1994;150(11):1801–9. 8199957 PMC1337055

[113] Tjia J, Field TS, Garber LD, Donovan JL, Kanaan AO, Raebel MA, et al Development and Pilot Testing of Guidelines to Monitor High-Risk Medications in the Ambulatory Setting. American Journal of Managed Care. 2010;16(7):489–96. WOS:000280636300001. 20645664

[114] van Dijk KN, Pont LG, de Vries CS, Franken M, Brouwers J, de Jong-van den erg LTW. Prescribing indicators for evaluating drug use in nursing homes. Annals of Pharmacotherapy. 2003;37(7–8):1136–41. 10.1345/aph.1C073 WOS:000184075800033. 12841830

[115] Williams D, Bennett K, Feely J. The application of prescribing indicators to a primary care prescription database in Ireland. European Journal of Clinical Pharmacology. 2005;61(2):127–33. 10.1007/s00228-004-0876-3 WOS:000228859200008. 15711833

[116] Winit-Watjana W, Sakulrat P, Kespichayawattana J. Criteria for high-risk medication use in Thai older patients. Archives of Gerontology and Geriatrics. 2008;47(1):35–51. 10.1016/j.archger.2007.06.006 WOS:000257487200005. 17675177

[117] Yu S, Galanter WL, DiDomenico RJ, Borkowsky S, Schiff GD, Lambert BL. Selection of drug-laboratory result pairs for an inpatient asynchronous alert program: results of a Delphi survey. American journal of health-system pharmacy: AJHP: official journal of the American Society of Health-System Pharmacists. 2011;68(5):407–14. 10.2146/ajhp100215. 21330682

[118] Zhan CL, Sangl J, Bierman AS, Miller MR, Friedman B, Wickizer SW, et al Potentially inappropriate medication use in the community-dwelling elderly—Findings from the 1996 Medical Expenditure Panel Survey. Jama-Journal of the American Medical Association. 2001;286(22):2823–9. WOS:000172655400030. 10.1001/jama.286.22.2823 11735757

[119] Guthrie B, Yu N, Murphy D, Donnan PT, Dreischulte T. Measuring prevalence, reliability and variation in high-risk prescribing in general practice using multilevel modelling of observational data in a population database. Health Services and Delivery Research. 2015 10.3310/hsdr03420 . 26539601

[120] Helmer-Hirschberg O. Analysis of the future: The Delphi method. Santa Monica, CA: RAND Corporation, 1967.

[121] Fitch K, Bernstein SJ, Aguilar MD, Burnand B, LaCalle JR, Lazaro P, et al The Rand/UCLA appropriateness method user's manual. Santa Monica, CA: RAND Corporation; 2001.

[122] Ross M, Wei W, Ohno-Machado L. “Big data” and the electronic health record. Yearbook of medical informatics. 2014;9(1):97. 25123728 10.15265/IY-2014-0003 PMC4287068

[123] Duerden M, Millson D, Avery A, Smart S. The quality of GP prescribing: The King’s Fund 2011 [cited 2018 April 2]. Available from: https://www.kingsfund.org.uk/sites/default/files/field/field_document/quality-gp-prescribing-gp-inquiry-research-paper-mar11.pdf.

[124] Lilford R, Stirling S, Maillard N. Citation classics in patient safety research: an invitation to contribute to an online bibliography. BMJ Quality & Safety. 2006;15(5):311–3. 10.1136/qshc.2005.017178 PMC2565810 17074864

[125] Ivers N, Jamtvedt G, Flottorp S, Young JM, Odgaard-Jensen J, French SD, et al Audit and feedback: effects on professional practice and healthcare outcomes. Cochrane Database of Systematic Reviews. 2012;(6). 10.1002/14651858.CD000259.pub3 CD000259 22696318 PMC11338587

[126] Boulkedid R, Abdoul H, Loustau M, Sibony O, Alberti C. Using and reporting the Delphi method for selecting healthcare quality indicators: a systematic review. PloS one. 2011;6(6):e20476 10.1371/journal.pone.0020476 21694759 PMC3111406

[127] Center for Health Policy/Center for Primary Care and Outcomes Research, Battelle Memorial Institute. Quality Indicator Measure Development, Implementation, Maintenance, and Retirement (Prepared by Battelle, under Contract No. 290-04-0020) Rockville, MD: Agency for Healthcare Research and Quality; 2011 [cited 2018 July 4]. Available from: https://www.qualityindicators.ahrq.gov/Downloads/Resources/Publications/2011/QI_Measure_Development_Implementation_Maintenance_Retirement_Full_5-3-11.pdf

[128] Humphrey-Murto S, Varpio L, Gonsalves C, Wood TJ. Using consensus group methods such as Delphi and Nominal Group in medical education research. Medical Teacher. 2017;39(1):14–9. 10.1080/0142159X.2017.1245856 27841062

[129] Keers RN, Williams SD, Vattakatuchery JJ, Brown P, Miller J, Prescott L, et al Prevalence, nature and predictors of prescribing errors in mental health hospitals: a prospective multicentre study. BMJ Open. 2014;4(9):e006084 10.1136/bmjopen-2014-006084 25273813 PMC4185335

[130] Keers RN, Williams SD, Vattakatuchery JJ, Brown P, Miller J, Prescott L, et al Medication safety at the interface: evaluating risks associated with discharge prescriptions from mental health hospitals. Journal of Clinical Pharmacy and Therapeutics. 2015;40(6):645–54. 10.1111/jcpt.12328 26534824

[131] Joint Formulary Committee. British National Formulary (online) London: BMJ Group and Pharmaceutical Press; 2018 [cited 2018 July 17]. Available from: http://www.medicinescomplete.com.

[132] Kessler RC, Berglund P, Demler O, Jin R, Merikangas KR, Walters EE. Lifetime prevalence and age-of-onset distributions of DSM-IV disorders in the National Comorbidity Survey Replication. Archives of general psychiatry. 2005;62(6):593–602. 10.1001/archpsyc.62.6.593 15939837

[133] Green H, McGinnity Á, Meltzer H, Ford T, Goodman R. Mental health of children and young people in Great Britain, 2004 2005 [cited 2018 September 5]. Available from: https://files.digital.nhs.uk/publicationimport/pub06xxx/pub06116/ment-heal-chil-youn-peop-gb-2004-rep1.pdf.

[134] Mental Health Foundation. Fundamental Facts About Mental Health 2016. London: Mental Health Foundation; 2016.

[135] MHRA. Valproate medicines (Epilim▼, Depakote▼): contraindicated in women and girls of childbearing potential unless conditions of Pregnancy Prevention Programme are met. Drug Safety Update. 2018;11(9):1.

[136] University of Nottingham. PINCER tool [cited 2018 June 25]. Available from: https://www.nottingham.ac.uk/primis/tools-audits/tools-audits/pincer/pincer.aspx.

[137] Thomas S, Westwood D, Goatley H, Fox A, Slee A, Coleman J. The development of an electronic data capture tool for high-risk prescribing errors [cited 2018 June 25]. Available from: http://www.eprescribingtoolkit.com/wp-content/uploads/2013/11/iMPACT_-_Quality_and_Safety_-_ThomasSK-ColemanJJ1.pdf.

[138] Williams R, Keers R, Gude WT, Jeffries M, Davies C, Brown B, et al SMASH! The Salford medication safety dashboard. Journal of Innovation in Health Informatics. 2018;25(3):183–93. 10.14236/jhi.v25i3.1015 30398462

[139] Jeffries M, Keers RN, Phipps DL, Williams R, Brown B, Avery AJ, et al Developing a learning health system: Insights from a qualitative process evaluation of a pharmacist-led electronic audit and feedback intervention to improve medication safety in primary care. PLOS ONE. 2018;13(10):e0205419 10.1371/journal.pone.0205419 30365508 PMC6203246

[140] Phil E, Mike C, Carolyn D, Sarah P, Ian R, Reinhard W. Identification of randomized controlled trials in systematic reviews: accuracy and reliability of screening records. Statistics in Medicine. 2002;21(11):1635–40. 10.1002/sim.1190 12111924

