# Supplementary material for: Identifying potential prescribing safety indicators related to mental health disorders and medications: A systematic review
Source: PLoS One. 2019 May 24;14(5):e0217406. doi: 10.1371/journal.pone.0217406 (PMC6534318; doi:10.1371/journal.pone.0217406)
Supplement: S1 File — (PDF) [file pone.0217406.s001.pdf]

Database(s): **Embase**
Search Strategy:

#	Searches
1	medication safety.mp.
2	medication error*.mp. or exp medication error/
3	prescribing error*.mp.
4	prescription error*.mp.
5	prescribing fault*.mp.
6	monitoring error*.mp.
7	inappropriate prescribing.mp. or exp inappropriate prescribing/
8	inappropriate medication*.mp. or exp potentially inappropriate medication/
9	irrational prescribing.mp.
10	prescribing appropriateness.mp.
11	appropriate prescribing.mp.
12	hazardous prescribing.mp.
13	drug-related morbidity.mp.
14	(prescribing adj3 safety).mp.
15	(prescribing adj3 quality).mp.
16	(inappropriate adj3 prescribing).mp.
17	high risk prescribing.mp.
18	high risk medication*.mp.
19	prescription error*.mp.
20	Medication related problem*.mp.
21	Drug related problem*.mp.
22	Guideline*.mp.
23	quality assurance.mp.
24	tool*.mp.
25	toolkit*.mp.
26	criteri*.mp.
27	instrument*.mp.
28	scale*.mp.
29	screen*.mp.
30	indicator*.mp.
31	measur*.mp.
32	list.mp.

33	outcome assessment*.mp.
34	patient reported outcome*.mp.
46	exp indicator/ or exp outcome assessment/ or exp patient reported outcome/
35	creat*.mp.
36	updat*.mp.
37	develop*.mp.
38	valid*.mp.
39	design*.mp.
40	consensus*.mp.
41	Delphi.mp.
42	rand appropriate*.mp.
43	revis*.mp.
44	Amend*.mp.
45	nominal group technique.mp.
47	22 or 23 or 24 or 25 or 26 or 27 or 28 or 29 or 30 or 31 or 32 or 33 or 34 or 46
48	35 or 36 or 37 or 38 or 39 or 40 or 41 or 42 or 43 or 44 or 45
49	1 or 2 or 3 or 4 or 5 or 6 or 7 or 8 or 9 or 10 or 11 or 12 or 13 or 14 or 15 or 16 or 17 or 18 or 19 or 20 or 21
50	47 and 48 and 49
51	limit 50 to yr="1990 -Current"
