# Supplementary material for: Identifying potential prescribing safety indicators related to mental health disorders and medications: A systematic review
Source: PLoS One. 2019 May 24;14(5):e0217406. doi: 10.1371/journal.pone.0217406 (PMC6534318; doi:10.1371/journal.pone.0217406)
Supplement: S2 File — (PDF) [file pone.0217406.s002.pdf]

# List of prescribing indicators related to mental health medications and conditions

## PIM: Independent of Diagnoses or Conditions

Prescribing [Class/Medication] to a patient aged [Age]

**Table 1: PIM: Independent of Diagnoses or Conditions (Antipsychotics)**

| Class/Medication | Age | References |
|---|---|---|
| **Antipsychotics** | ≥ 65 | [25, 34, 38, 42, 43] |
| | ≥ 70 | [95] |
| | 0–5 | [69] |
| | NH | [92] |
| *Atypical antipsychotics* | ≥ 65 | [31, 32] |
| *Aripiprazole* | ≥ 65 | [46, 91] |
| *Asenapine* | ≥ 65 | [91] |
| *Chlorpromazine* | ≥ 65 | [46, 47, 66, 84, 91, 116] |
| | ≥ 70 | [96] |
| | ≥ 75 | [87] |
| *Chlorprothixen* | ≥ 65 | [46] |
| | ≥ 70 | [96] |
| *Clozapine* | ≥ 65 | [45-47, 66, 77, 84, 90, 91, 116] |
| *Cyamemazine* | ≥ 65 | [46] |
| | ≥ 75 | [87] |
| *Droperidol* | ≥ 65 | [46] |
| *Flupentixol* | ≥ 65 | [46] |
| *Fluphenazine* | ≥ 65 | [45, 46, 90] |
| | ≥ 75 | [87] |
| *Haloperidol* | ≥ 65 | [84, 90, 91, 116] |
| *Levomepromazine* | ≥ 65 | [46, 47, 66, 91] |
| | ≥ 70 | [96] |
| | ≥ 75 | [87] |
| *Loxapine* | ≥ 65 | [46, 66] |
| *Olanzapine* | ≥ 65 | [84, 90, 91, 116] |
| *Paliperidone* | ≥ 65 | [91] |
| *Perphenazine* | ≥ 65 | [45, 46, 90, 91] |
| | ≥ 75 | [87] |
| *Pimozide* | ≥ 65 | [46, 91] |
| *Pipotiazine* | ≥ 65 | [46, 91] |
| | ≥ 75 | [87] |
| *Prochlorperazine* | ≥ 65 | [46] |
| | ≥ 70 | [96] |
| *Propericiazine (periciazine)* | ≥ 65 | [46, 91] |
| | ≥ 75 | [87] |
| *Prothipendyl* | ≥ 65 | [90] |
| *Risperidone* | ≥ 65 | [84, 91, 116] |
| *Reserpine* | ≥ 65 | [41, 46, 66, 118] |
| | ≥ 75 | [87] |
| *Sertindole* | ≥ 65 | [46] |
| *Sulpiride* | ≥ 75 | [85] |
| *Thioridazine* | ≥ 65 | [46, 66, 91, 116] |
| *Trifluoperazine* | ≥ 65 | [46, 91] |
| *Ziprasidone* | ≥ 65 | [46, 91] |
| *Zuclopenthixol* | ≥ 65 | [46, 84] |

**Table 2: PIM: Independent of Diagnoses or Conditions (Antidepressants)**

| Class/Medication | Age | References |
|---|---|---|
| **Antidepressants** | ≥ 65 | [42] |
| | 0–5 | [69] |
| | NH | [92] |
| ***TCA*** | ≥ 65 | [32, 34, 38, 65] |
| | ≥ 70 | [95] |
| | ≥ 75 | [73, 85] |
| | ≤ 18 | [103] |
| *Amitriptyline* | ≥ 65 | [31, 34, 41, 45-47, 66, 77, 84, 90, 91, 93, 106, 108, 116, 118] |
| | ≥ 70 | [96] |
| | ≥ 75 | [87] |

| | | |
|---|---|---|
| *Amoxapine* | ≥ 65 | [34, 46, 84] |
| | ≥ 75 | [87] |
| *Clomipramine* | ≥ 65 | [34, 45-47, 66, 77, 84, 90, 91] |
| | ≥ 70 | [96] |
| | ≥ 75 | [87] |
| *Desipramine* | ≥ 65 | [34, 46] |
| *Dosulepin* | ≥ 65 | [46, 47, 66] |
| | ≥ 75 | [87] |
| *Doxepin* | ≥ 65 | [41, 45, 46, 66, 77, 90, 106, 108, 116, 118] |
| | ≥ 70 | [96] |
| | ≥ 75 | [87] |
| *Imipramine* | ≥ 65 | [34, 45-47, 66, 77, 84, 91, 93, 116] |
| | ≥ 75 | [87] |
| *Maprotiline* | ≥ 65 | [45, 46, 77, 90] |
| | ≥ 75 | [87] |
| *Melitracen* | ≥ 65 | [66] |
| *Nortriptyline* | ≥ 65 | [34, 46, 84, 91] |
| *Protriptyline* | ≥ 65 | [34] |
| *Trimipramine* | ≥ 65 | [34, 45, 46, 77] |
| | ≥ 70 | [96] |
| | ≥ 75 | [87] |
| ***SSRI/SNRI*** | ≥ 65 | [34] |
| *SSRI other than fluoxetine* | ≤ 18 | [73, 103] |
| *Fluvoxamine* | ≥ 65 | [46, 90] |
| *Fluoxetine* | ≥ 65 | [31, 45-47, 77, 91] |
| | ≤ 18 | [73] |
| *Paroxetine* | ≥ 65 | [34, 46] |
| *Venlafaxine* | ≥ 65 | [46] |
| ***NDRI*** *(Bupropion)* | ≥ 65 | [46] |
| ***NRI*** *(Reboxetine)* | ≥ 65 | [46] |
| ***MAOi*** *(Tranylcypromine)* | ≥ 65 | [45, 46, 77, 91] |
| ***MAOi*** *(Bifemelane)* | ≥ 65 | [91] |

MAOi: Monoamine oxidase inhibitor. NDRI: Norepinephrine–dopamine reuptake inhibitor. NRI: Norepinephrine reuptake inhibitor. SNRI: Serotonin and norepinephrine reuptake inhibitors. SSRI: Selective serotonin reuptake inhibitor. TCA: Tricyclic antidepressants.

**Table 3: PIM: Independent of Diagnoses or Conditions (Sedative, hypnotics and anxiolytics)**

| Class/Medication | Age | References |
|---|---|---|
| **Long acting Hypnotics or anxiolytic** | ≥ 65 | [38, 65] |
| **Benzodiazepine** | ≥ 65 | [25, 42, 43, 68] |
| | ≥ 75 | [85] |
| | NH | [92] |
| *long acting benzodiazepine* | ≥ 65 | [41, 48, 73, 74, 107, 108, 112] |
| | ≥ 75 | [87] |
| *short acting benzodiazepine* | ≥ 65 | [48] |
| | ≥ 75 | [73] |
| *Other than Temazepam or Zolpidem.* | ≥ 65 | [92] |
| *Alprazolam* | ≥ 65 | [34, 45, 46, 84, 91, 116] |
| *Brotizolam* | ≥ 65 | [90] |
| *Bromazepam* | ≥ 65 | [45, 46, 84, 90, 91] |
| *Chlordiazepoxide* | ≥ 65 | [34, 39, 45-47, 66, 84, 90, 91, 106, 116, 118] |
| *Clobazam* | ≥ 65 | [45-47, 66, 84, 90, 91] |
| *Clonazepam* | ≥ 65 | [34, 46, 66, 84, 90, 91] |
| *Clorazepate* | ≥ 65 | [34, 45, 46, 66, 90, 91] |
| *Diazepam* | ≥ 65 | [34, 39, 45-47, 66, 84, 90, 91, 106, 116, 118] |
| | ≥ 70 | [95, 96] |
| *Estazolam* | ≥ 65 | [34] |
| *Fludiazepam* | ≥ 65 | [66] |
| *Flunitrazepam* | ≥ 65 | [45, 46, 66, 84, 90, 91] |
| | ≥ 70 | [95, 96] |
| *Flurazepam* | ≥ 65 | [34, 39, 41, 45, 46, 66, 84, 106, 116, 118] |
| *Halazepam* | ≥ 65 | [46] |
| *Loflazepate* | ≥ 65 | [46] |
| *Lorazepam* | ≥ 65 | [34, 84, 90, 91, 116] |
| *Medazepam* | ≥ 65 | [45-47] |
| *Midazolam* | ≥ 65 | [46, 47] |
| *Nitrazepam* | ≥ 65 | [45, 46, 66, 90] |
| | ≥ 70 | [95, 96] |
| *Nordazepam* | ≥ 65 | [46, 66] |
| *Oxazepam* | ≥ 65 | [34, 90, 91] |
| *Oxazolam* | ≥ 65 | [66] |
| *Prazepam* | ≥ 65 | [45, 46, 90] |

| | | |
|---|---|---|
| *Quazepam* | ≥ 65 | [34, 46] |
| *Temazepam* | ≥ 65 | [34, 45, 46, 84] |
| *Triazolam* | ≥ 65 | [34, 45, 46, 84, 90, 116] |
| **Z-drugs** | ≥ 65 | [25, 42] |
| | ≥ 75 | [73, 85] |
| *Eszopiclone* | ≥ 65 | [34, 91] |
| *Zaleplon* | ≥ 65 | [34, 91] |
| *Zolpidem* | ≥ 65 | [34, 84, 91] |
| *Zopiclone* | ≥ 65 | [91] |
| **Barbiturates** | ≥ 65 | [39, 118] |
| *Amobarbital* | ≥ 65 | [66] |
| *Butabarbital* | ≥ 65 | [34] |
| *Butalbital* | ≥ 65 | [34] |
| *Mephobarbital* | ≥ 65 | [34] |
| *Pentobarbital* | ≥ 65 | [34, 45, 66, 91] |
| *Phenobarbital* | ≥ 65 | [34, 41, 46, 48, 90, 91, 116] |
| *Secobarbital* | ≥ 65 | [34, 41, 66] |
| *Thiopental* | ≥ 65 | [91] |
| *Chloral hydrate* | ≥ 65 | [41, 45, 46] |
| *Clomethiazole* | ≥ 65 | [46] |
| | ≥ 70 | [95] |
| *Meprobamate* | ≥ 65 | [34, 39, 41, 46, 66, 118] |
| *First-generation antihistamines* | ≥ 65 | [25, 42, 43] |
| | ≥ 70 | [95] |
| *Sedating antihistamine* | ≥ 65 | [48] |
| *Alimemazine* | ≥ 75 | [87] |
| | ≥ 70 | [96] |
| | ≥ 65 | [46, 66, 108] |
| *Azatadine* | ≥ 65 | [66] |
| *Brompheniramine* | ≥ 65 | [34, 46, 66, 91] |
| | ≥ 75 | [87] |
| *Buclizine* | ≥ 65 | [46, 66] |
| | ≥ 75 | [87] |
| *Carbinoxamine* | ≥ 65 | [34, 46, 66, 91] |
| | ≥ 75 | [87] |
| *Chlorcyclizine* | ≥ 65 | [66] |
| *Chlorpheniramine* | ≥ 65 | [34, 46, 66, 84, 91, 118] |
| | ≥ 75 | [85, 87] |
| *Chlorphenoxamine* | ≥ 65 | [66] |
| *Clemastine* | ≥ 65 | [34, 46, 47, 66] |
| *Cyclizine* | ≥ 65 | [46, 66] |
| *Cyproheptadine* | ≥ 65 | [34, 46, 47, 66, 91, 118] |
| | ≥ 75 | [87] |
| *Dexbrompheniramine* | ≥ 65 | [34] |
| *Dexchlorpheniramine* | ≥ 65 | [34, 46, 66, 91] |
| | ≥ 70 | [96] |
| | ≥ 75 | [87] |
| *Dimenhydrinate* | ≥ 65 | [34, 46, 47, 84] |
| | ≥ 75 | [87] |
| *Dimetindene* | ≥ 65 | [46] |
| *Diphenhydramine* | ≥ 65 | [34, 45, 46, 66, 84, 91, 118] |
| | ≥ 75 | [85, 87] |
| *Diphenylpyraline* | ≥ 65 | [66] |
| *Doxylamine* | ≥ 65 | [34, 45, 46, 66, 91] |
| | ≥ 75 | [87] |
| *Ebastine* | ≥ 65 | [46] |
| *Homochlorcyclizine* | ≥ 65 | [66] |
| *Hydroxyzine* | ≥ 65 | [34, 46, 47, 77, 84, 91, 118] |
| | ≥ 70 | [96] |
| | ≥ 75 | [87] |
| *Ketotifen* | ≥ 65 | [66] |
| *Mebhydrolin* | ≥ 65 | [66] |
| *Meclizine* | ≥ 65 | [34, 46, 66] |
| *Mepyramine* | ≥ 65 | [66] |
| *Mequitazine* | ≥ 65 | [46, 66, 91] |
| | ≥ 75 | [87] |
| *Oxomemazine* | ≥ 65 | [46] |
| *Oxatomide* | ≥ 65 | [66] |
| *Phenindamine* | ≥ 65 | [66] |
| *Pheniramine* | ≥ 65 | [66] |
| | ≥ 75 | [87] |

| | | |
|---|---|---|
| *Pimethixene* | ≥ 65 | [46] |
| *Promethazine* | ≥ 65 | [34, 46, 47, 66, 91, 108, 118] |
| | ≥ 70 | [96] |
| | ≥ 75 | [87] |
| *Propiomazine* | ≥ 65 | [46] |
| *Terfenadine* | ≥ 65 | [46] |
| *Tripelennamine* | ≥ 65 | [46, 66] |
| *Triprolidine* | ≥ 65 | [34, 46, 66, 84] |
| | ≥ 75 | [85] |
| *Aceprometazine* | ≥ 65 | [46] |
| | ≥ 75 | [87] |
| *Phenothiazine* | ≤ 20 | [73] |
| | ≥ 65 | [32] |
| *Propranolol* | ≥ 65 | [41, 46] |

**Table 4: PIM: Independent of Diagnoses or Conditions (Anti-dementia)**

| Class/Medication | Age | References |
|---|---|---|
| *Anticholinesterase inhibitors* | ≥ 70 | [95] |
| *Aniracetam* | ≥ 65 | [91] |
| *Citicoline* | ≥ 65 | [91] |
| *Cyclandelate* | ≥ 65 | [41, 46] |
| *Dihydroergocristine* | ≥ 65 | [46, 47, 77] |
| | ≥ 75 | [87] |
| *Dihydroergocryptine* | ≥ 75 | [87] |
| *Dihydroergotoxine* | ≥ 65 | [34, 41, 46, 47, 116] |
| | ≥ 75 | [87] |
| *Ginkgo biloba* | ≥ 65 | [46-48, 77, 91] |
| | ≥ 75 | [87] |
| *Idebenone* | ≥ 65 | [91] |
| *Isoxsuprine* | ≥ 65 | [34, 41] |
| *Moxisylyte* | ≥ 65 | [46] |
| *Naftidrofuryl* | ≥ 65 | [45-47] |
| | ≥ 75 | [87] |
| *Nicergoline,* | ≥ 65 | [45-47, 77] |
| | ≥ 75 | [87] |
| *Pentoxifylline* | ≥ 65 | [45-47, 77] |
| | ≥ 75 | [87] |
| *Piracetam* | ≥ 65 | [45-47, 77, 91] |
| | ≥ 75 | [87] |
| *Piribedil* | ≥ 75 | [87] |
| *Vinburnine* | ≥ 65 | [46, 77] |
| | ≥ 75 | [87] |
| *Vincamine* | ≥ 65 | [46, 77] |
| | ≥ 75 | [87] |

**Table 5: PIM: Independent of Diagnoses or Conditions (ADHD medications)**

| Class/Medication | Age | References |
|---|---|---|
| *All ADHD Meds* | < 6 | [103] |
| *Atomoxetine* | 0–4 | [69] |
| *Clonidine* | ≥ 65 | [31, 34, 46, 48, 66, 91, 116] |
| | ≥ 75 | [87] |
| *Guanfacine* | ≥ 65 | [34, 46] |
| | ≥ 75 | [87] |
| *Methylphenidate* | ≥ 65 | [46, 91] |
| *Stimulants* | 0–4 | [69] |

**Table 6: PIM: Independent of Diagnoses or Conditions (Mood stabilisers)**

| Class/Medication | Age | References |
|---|---|---|
| *Carbamazepine* | ≥ 65 | [34, 46, 91] |
| *Lithium* | ≥ 65 | [46] |

**Table 7: PIM: Independent of Diagnoses or Conditions (Anticholinergics)**

| Class/Medication | Age | References |
|---|---|---|
| *Anticholinergics* | ≥ 65 | [38] |
| | ≥ 75 | [87] |
| | NH | [92] |
| *High anticholinergic Meds* | ≥ 65 | [64, 107] |
| *Atropine (excludes ophthalmic)* [a] | ≥ 65 | [34] |
| *Belladonna* [a] | ≥ 65 | [34, 41, 46, 66, 91, 116, 118] |
| | ≥ 75 | [87] |
| *Benzatropine* [a] | ≥ 65 | [34, 46, 84] |
| *Biperiden* [a] | ≥ 65 | [46, 90, 91] |

| | | |
|---|---|---|
| | ≥ 75 | [85] |
| *Bornaprine* [a] | ≥ 65 | [84, 90] |
| *Clidinium-chlordiazepoxide* [a] | ≥ 65 | [34, 46] |
| | ≥ 75 | [87] |
| *Dicyclomine (Dicyclomine)* [a] | ≥ 65 | [34, 118] |
| *Dihexyverine* [a] | ≥ 75 | [87] |
| *Diphenoxylate-atropine* [a] | ≥ 75 | [87] |
| *Homatropine methylbromide* [a] | ≥ 65 | [91] |
| *Hyoscine* [a] | ≥ 65 | [34, 41, 46, 84, 91, 116] |
| | ≥ 75 | [87] |
| *Hyoscyamine* [a] | ≥ 65 | [34, 41, 46, 66, 118] |
| *Orphenadrine* [a] | ≥ 65 | [31, 34, 41, 46, 66, 116] |
| *Propantheline* [a] | ≥ 65 | [34, 118] |
| *Tiemonium* [a] | ≥ 75 | [87] |
| *Trihexyphenidyl* [a] | ≥ 65 | [34, 46, 84, 91] |
| | ≥ 75 | [85] |
| *Tropatepine* [a] | ≥ 65 | [46] |

**a.** These medications were included because they can be used to treat some of the side effects caused by mental health medications.

## PIM: considering diagnoses or conditions

Prescribing [Class/Medication] to a patient with [Condition] +/- and aged [Age]

**Table 8: PIM: considering diagnoses or conditions (Antipsychotics)**

| CONDITION | CLASS/MEDICATION | AGE | REFERENCE |
|---|---|---|---|
| **Dementia or Cognitive Impairment** | *Antipsychotics* | ≥ 75 | [62, 85] |
| | *Antipsychotics* | ≥ 65 | [34, 84] |
| | *Perphenazine* | ≥ 65 | [83] |
| | *Clozapine* | ≥ 65 | [83] |
| | *Haloperidol* | ≥ 65 | [83] |
| | *Olanzapine* | ≥ 65 | [83] |
| | *Antipsychotic other than risperidone and olanzapine* | ≥ 75 | [87] |
| **Dementia but Not Psychosis** | *Antipsychotics* | ≥ 65 | [73] |
| | *Risperidone* | ≥ 65 | [11] |
| | *Olanzapine* | ≥ 65 | [11] |
| **Dementia and Psychosis** | *Antipsychotic other than risperidone* | ≥ 65 | [73] |
| **BPSD** | *Antipsychotics* | ≥ 65 | [25, 34] |
| | *Antipsychotics* | ≥ 70 | [95] |
| | *Antipsychotic other than risperidone* | - | [15] |
| **Advanced dementia** | *Antipsychotics* | - | [86] |
| **Advanced dementia (palliative)** | *Antipsychotics* | - | [80] |
| **Seizures or Epilepsy** | *Antipsychotics* | ≥ 65 | [116] |
| | *Chlorpromazine* | ≥ 65 | [34] |
| | *Phenothiazines* | - | [71] |
| | | ≥ 65 | [43, 47] |
| | *Haloperidol* | - | [71] |
| | *Clozapine* | ≥ 65 | [34] |
| | *Thioridazine* | ≥ 65 | [34] |
| | *Thiothixene* | ≥ 65 | [34] |
| | *Olanzapine* | ≥ 65 | [34] |
| **Parkinson's Disease** | *Antipsychotics* | ≥ 65 | [42] |
| | *Antipsychotics other than quetiapine or clozapine* | ≥ 65 | [25, 32, 48, 73, 92] |
| | *Antipsychotics other than aripiprazole, quetiapine, clozapine* | ≥ 65 | [34, 84] |
| | *Prochlorperazine* | - | [15] |
| | | ≥ 65 | [25, 32, 34, 43] |
| | *Haloperidol* | ≥ 65 | [74, 83, 116] |
| | *Droperidol* | ≥ 65 | [74] |
| | *Perphenazine* | ≥ 65 | [83] |
| | *Clozapine* | ≥ 65 | [83] |
| | *Olanzapine* | ≥ 65 | [83] |
| **History of prostatism or previous urinary retention or BPH** | *Antipsychotics* | - | [71] |
| | *Chlorpromazine* | ≥ 65 | [25] |
| | *Clozapine* | ≥ 65 | [25] |
| | *Flupenthixol* | ≥ 65 | [25] |
| | *Fluphenazine* | ≥ 65 | [25] |

| | | | |
|---|---|---|---|
| | *Pipothiazine* | | [25] |
| | *Promazine* | | [25] |
| | *Zuclopenthixol* | | [25] |
| **Glaucoma** | *Fluphenazine* | - | [71] |
| | *Perphenazine* | | [71] |
| | *Trifluoperazine* | | [71] |
| **Syncope** | *Antipsychotics* | | [84] |
| | *Chlorpromazine* | | [34] |
| | *Thioridazine* | | [34] |
| | *Olanzapine* | | [34, 83] |
| | *Haloperidol* | | [83] |
| | *Clozapine* | | [83] |
| | *Perphenazine* | | [83] |
| **Postural Hypotension** | *Antipsychotics* | ≥ 65 | [84] |
| | *Thioridazine* | | [88] |
| | *Chlorpromazine* | | [93] |
| **History of Falls** | *Antipsychotics* | ≥ 65 | [34, 47, 84] |
| | *Conventional antipsychotics* | | [88] |
| | *Perphenazine* | | [83] |
| | *Clozapine* | | [83] |
| | *Haloperidol* | | [83] |
| | *Olanzapine* | | [83] |
| **Delirium** | *Antipsychotics* | ≥ 65 | [34, 84] |
| | *Chlorpromazine* | | [34] |
| | *Perphenazine* | | [83] |
| | *Clozapine* | | [83] |
| | *Haloperidol* | | [83] |
| | *Olanzapine* | | [83] |
| **Depression** | *Quetiapine* | ≥ 65 | [101] |
| | *Olanzapine* | | [101] |
| **ADHD without Hyperactivity** | *Antipsychotics* | Children | [103] |
| **Arrhythmia** | *Antipsychotics* | ≥ 65 | [116] |
| **HTN** | *Clozapine* | - | [71] |
| **Swallowing Problems** | *Antipsychotics* | ≥ 65 | [42] |
| **Lewy Body Disease** | *Antipsychotics other than quetiapine or clozapine* | ≥ 65 | [25, 32] |
| **Insomnia / Sleep Disorders** | *Pipamperone* | ≥ 65 | [101] |
| | *Melperone* | | [101] |
| **DM** | *Antipsychotics* | - | [71] |
| **Frail Adults with Limited Life Expectancy** | *Antipsychotics* | - | [78] |
| **Chronic constipation** | *Perphenazine* | ≥ 65 | [83] |
| | *Clozapine* | | [83] |
| | *Haloperidol* | | [83] |
| | *Olanzapine* | | [83] |
| **QTC > 450/470MS** | *Haloperidol* | ≥ 65 | [104] |
| **Absolute neutrophil count < 1.5*109/L** | *Clozapine* | ≥ 65 | [104] |

ADHD: Attention deficit hyperactivity disorder. BPH: Benign prostatic hyperplasia. BPSD: Behavioural and Psychological Symptoms of Dementia. DM: Diabetes mellitus. HTN: Hypertension.

**Table 9: PIM: Considering Diagnoses or Conditions (Antidepressants)**

| CONDITION | CLASS/MEDICATION | AGE | REFERENCE |
|---|---|---|---|
| **Heart block** | *TCA* | ≥ 65 | [88, 93, 116] |
| | *Amitriptyline at dose >75mg* | - | [10] |
| **Cardiac conduction abnormalities** | *TCA* | ≥ 65 | [25, 43, 68] |
| | *Amitryptyline* | | [83] |
| | *Clomipramine* | | [83] |
| | *Imipramine* | | [83] |
| **Cardiovascular risk factors or CVD** | *TCA* | ≥ 65 | [92] |
| **Heart failure** | *Amitriptyline at dose >75mg* | - | [10] |
| | *TCA* | - | [11, 71, 73] |
| | *TCA* | ≥ 65 | [47, 84] |
| **Arrhythmia** | *TCA* | ≥ 65 | [84] |
| | *Amitriptyline at dose >75mg* | - | [10] |
| **HTN** | *Venlafaxine* | - | [71] |
| | *Duloxetine* | | [71] |
| | *MAOIs* | | [71] |
| **Postural hypotension** | *Amitriptyline at dose >75mg* | - | [10] |
| | *TCA* | ≥ 65 | [88, 92, 93] |
| **Syncope** | *TCA* | ≥ 65 | [47, 116] |

| | | | |
|---|---|---|---|
| | *Tertiary TCAs* | | [34] |
| | *Amitriptyline* | | [83] |
| | *Clomipramine* | | [83] |
| | *Imipramine* | | [83] |
| **History of falls** | *SSRI* | ≥ 65 | [34, 47, 88] |
| | *Amitriptyline* | | [83] |
| | *Clomipramine* | | [83] |
| | *Imipramine* | | [83] |
| **Seizures or epilepsy** | *SSRI* | - | [15, 71] |
| | *TCA* | - | [71] |
| | *Bupropion* | - | [10, 39, 71] |
| | *Bupropion* | ≥ 65 | [34, 88] |
| | *Maprotiline* | ≥ 65 | [34] |
| **Dementia or cognitive impairment** | *Antidepressants* | ≥ 70 | [95] |
| | *TCA* | ≥ 65 | [25, 43, 47, 68, 73, 88, 116] |
| | *TCA* | - | [15] |
| | *Amitriptyline* | ≥ 65 | [83] |
| | *Clomipramine* | ≥ 65 | [83] |
| | *Imipramine* | ≥ 65 | [83] |
| | *Selegiline* | ≥ 65 | [101] |
| **Advanced dementia** | *TCA* | - | [86] |
| | *Antidepressants other than TCA* | | [86] |
| **Advanced dementia (Palliative)** | *TCA* | - | [80] |
| | *Antidepressants other than TCA* | | [80] |
| **BPSD: depression** | *Citalopram* | ≥ 65 | [101] |
| | *Escitalopram* | | [101] |
| | *Sertraline* | | [101] |
| | *Fluoxetine* | | [101] |
| | *Venlafaxine* | | [101] |
| | *Duloxetine* | | [101] |
| **Glaucoma** | *TCA* | ≥ 65 | [25, 43, 47, 68, 92, 93, 116] |
| | *TCA* | - | [71] |
| | *Amitriptyline* | ≥ 65 | [83] |
| | *Clomipramine* | ≥ 65 | [83] |
| | *Imipramine* | ≥ 65 | [83] |
| | *MAOI* | - | [71] |
| | *Citalopram* | - | [71] |
| | *Escitalopram* | - | [71] |
| | *Fluoxetine* | - | [71] |
| | *Fluvoxamine* | - | [71] |
| | *Mianserin* | - | [71] |
| | *Paroxetine* | - | [71] |
| **Depression** | *Nortriptyline* | ≥ 65 | [101] |
| | *Mirtazapine* | | [101] |
| | *Venlafaxine* | | [101] |
| | *Duloxetine* | | [101] |
| | *Moclobemide* | | [101] |
| | *Bupropion* | | [101] |
| | *Vortioxetine* | | [101] |
| | *Agomelatine* | | [101] |
| | *Reboxetine* | | [101] |
| | *Trazodone* | | [101] |
| | *St. John's Wort* | | [101] |
| **Insomnia / sleep disorders** | *Mirtazapine* | ≥ 65 | [101] |
| | *Doxepin* | | [101] |
| | *Opipramol* | | [101] |
| | *Fluoxetine* | | [47] |
| | *MAO* | | [47] |
| **Prostatism or history of urinary retention or BPH** | *TCA* | - | [71] |
| | *TCA* | ≥ 65 | [25, 43, 47, 68, 88, 92, 93] |
| | *Amitriptyline* | | [83] |
| | *Clomipramine* | | [83] |
| | *Imipramine* | | [83] |
| **Urinary incontinence** | *TCA* | ≥ 65 | [47, 116] |
| **Constipation** | *TCA* | ≥ 65 | [43, 47, 68, 88, 116] |
| | *Amitriptyline* | | [83] |
| | *Clomipramine* | | [83] |
| | *Imipramine* | | [83] |
| **Current or recent significant hyponatraemia** | *SSRI* | ≥ 65 | [25, 43, 47, 92] |
| | *SSRI* | - | [15] |
| **Renal failure** | *Paroxetine* | - | [71] |

| | | | |
|---|---|---|---|
| **Hepatic impairment or cirrhosis** | *TCA* | - | [71] |
| **Gastrointestinal haemorrhage** | *Paroxetine* | ≥ 75 | [85] |
| | *Sertraline* | ≥ 75 | [85] |
| | *Fluvoxamine* | ≥ 75 | [85] |
| | *Escitalopram* | ≥ 75 | [85] |
| **Peptic ulcer disease** | *SSRI* | - | [71] |
| **Bladder atony due to diabetes** | *Imipramine* | ≥ 65 | [38] |
| **Anorexia and malnutrition** | *Fluoxetine* | ≥ 65 | [47] |
| **Delirium** | *Amitriptyline* | ≥ 65 | [83] |
| | *Clomipramine* | | [83] |
| | *Imipramine* | | [83] |
| **Acute bipolar depression** | *TCA* | Adults | [49] |
| **Acute management of depressive bipolar disorder** | *paroxetine* | Adults | [49] |

BPH: Benign prostatic hyperplasia. BPSD: Behavioural and Psychological Symptoms of Dementia. CVD: Cardiovascular disease. HTN: Hypertension. MAOi: Monoamine oxidase inhibitor. SSRI: Selective serotonin reuptake inhibitor. TCA: Tricyclic antidepressants.

**Table 10: PIM: Considering Diagnoses or Conditions (Sedative, hypnotics, and anxiolytics)**

| CONDITION | CLASS/MEDICATION | AGE | REFERENCE |
|---|---|---|---|
| **Dementia or cognitive impairment** | *Benzodiazepines* | ≥ 75 | [87] |
| | *Benzodiazepines* | ≥ 65 | [34, 66, 84, 88] |
| | *Alprazolam* | | [83] |
| | *Clorazepam* | | [83] |
| | *Triazolam* | | [83] |
| | *Chlorazepate* | | [83] |
| | *Chlordiazepoxide* | | [83] |
| | *Diazepam* | | [83] |
| | *Flurazepam* | | [83] |
| | *Eszopiclone* | | [34] |
| | *Zolpidem* | | [34, 84] |
| | *Zaleplon* | | [34] |
| | *Barbiturates* | | [88] |
| **History of falls or fractures** | *Sedative-hypnotics* | ≥ 65 | [88] |
| | *Benzodiazepines* | | [34, 47, 84, 88] |
| | *Alprazolam* | | [83] |
| | *Clorazepam* | | [83] |
| | *Triazolam* | | [83] |
| | *Chlorazepate* | | [83] |
| | *Chlordiazepoxide* | | [83] |
| | *Diazepam* | | [83] |
| | *Flurazepam* | | [83] |
| | *Eszopiclone* | | [34] |
| | *Zolpidem* | | [34, 84] |
| | *Zaleplon* | | [34] |
| | *Chlorpheniramine* | | [83] |
| | *Clemastine* | | [83] |
| | *Doxylamine* | | [83] |
| | *Triprolidine* | | [83] |
| **Syncope** | *Benzodiazepines* | ≥ 65 | [84] |
| | *Zolpidem* | | [84] |
| **Postural hypotension** | *Benzodiazepines* | ≥ 65 | [84] |
| | *Zolpidem* | | [84] |
| **Acute or chronic respiratory failure** | *Benzodiazepines* | ≥ 65 | [25] |
| **Asthma** | *Benzodiazepines* | ≥ 65 | [116] |
| | *Propranolol* | | [85] |
| **COPD** | *long-acting benzodiazepine* | ≥ 65 | [47] |
| | *medium to long-acting benzodiazepine* | ≥ 65 | [38] |
| | *Benzodiazepines* | ≥ 65 | [64, 66, 116] |
| | *Benzodiazepines* | - | [15] |
| | *Z-drugs* | - | [15] |
| | *Propranolol* | ≥ 65 | [85] |
| **Sleep apnoea syndrome** | *Benzodiazepines* | ≥ 65 | [66, 116] |
| **Delirium** | *Benzodiazepines* | ≥ 65 | [34, 84] |
| | *Sedative- hypnotics* | | [34] |
| | *Alprazolam* | | [83] |
| | *Clorazepam* | | [83] |
| | *Triazolam* | | [83] |
| | *Chlorazepate* | | [83] |
| | *Chlordiazepoxide* | | [83] |
| | *Diazepam* | | [83] |
| | *Flurazepam* | | [83] |

| | | | |
|---|---|---|---|
| | *Zolpidem* | | [84] |
| **Depression** | *long-acting benzodiazepine* | ≥ 65 | [38, 101] |
| | *Barbiturates* | | [38] |
| | *Benzodiazepines* | | [47, 101] |
| | *Short acting benzodiazepine* | | [101] |
| **Insomnia / sleep disorders** | *Zopiclone* | ≥ 65 | [101] |
| | *Zolpidem* | | [101] |
| | *Zaleplon* | | [101] |
| | *Medium half-life Benzodiazepines* | | [101] |
| | *Very short half-life Benzodiazepines* | | [101] |
| | *Diphenhydramine* | | [101] |
| **Urinary incontinence** | *Benzodiazepines* | ≥ 65 | [66] |
| **Urinary retention** | *Benzodiazepines* | ≥ 65 | [66] |
| **BPH** | *Antihistamine* | ≥ 65 | [116] |
| **Advanced dementia** | *Antihistamine 1st generation* | - | [86] |
| **Parkinson disease** | *Promethazine* | ≥ 65 | [34] |
| **Hepatic impairment or cirrhosis** | *Benzodiazepines* | - | [71] |
| | *Barbiturates* | | [71] |
| **Chronic constipation** | *Chlorpheniramine* | ≥ 65 | [83] |
| | *Clemastine* | | [83] |
| | *Doxylamine* | | [83] |
| | *Triprolidine* | | [83] |
| | *antihistamines* | | [116] |

BPH: Benign prostatic hyperplasia. COPD: Chronic Obstructive Pulmonary Disease

**Table 11: PIM: Considering Diagnoses or Conditions (Mood stabilisers)**

| CONDITION | CLASS/MEDICATION | AGE | REFERENCE |
|---|---|---|---|
| **Heart failure** | *Carbamazepine* | - | [71] |
| **HTN** | *Carbamazepine* | - | [71] |
| **Bipolar disorder** | *Carbamazepine* | ≥ 65 | [101] |
| | *Valproic acid* | | [101] |
| | *Lamotrigine* | | [101] |
| | *Lithium* | | [101] |
| **Renal failure** | *Lithium* | - | [71] |
| **Rheumatoid arthritis** | *Lithium* | - | [71] |
| **Thyroid disorders** | *Lithium* | - | [71] |
| **Epilepsy** | *Lithium* | - | [71] |

HTN: Hypertension.

**Table 12: PIM: Considering Diagnoses or Conditions (Anti-dementia)**

| CONDITION | CLASS/MEDICATION | AGE | REFERENCE |
|---|---|---|---|
| **Persistent bradycardia** | *Acetylcholinesterase inhibitors* | ≥ 65 | [25, 42] |
| **Heart block** | *Acetylcholinesterase inhibitors* | ≥ 65 | [25] |
| **Recurrent unexplained syncope** | *Acetylcholinesterase inhibitors* | ≥ 65 | [34] |
| **Palliative care patients with advanced dementia** | *Acetylcholinesterase inhibitors* | - | [80] |
| | *Memantine* | | [80] |
| **Frail adults with limited life expectancy** | *Memantine* | - | [78] |
| **Dementia** | *Memantine* | ≥ 65 | [101] |
| | *Ginkgo biloba* | | [101] |
| | *Ergoline derivatives* | | [101] |
| | *Piracetam* | | [101] |
| **To treat dementia** | *Nylidrin* | ≥ 65 | [93] |
| | *Niacin* | | [93] |
| | *Pentoxifylline* | | [93] |

**Table 13: PIM: Considering Diagnoses or Conditions (Anticholinergics)**

| CONDITION | CLASS/MEDICATION | AGE | REFERENCE |
|---|---|---|---|
| **Dementia or cognitive impairment** | *Anticholinergics* | - | [39] |
| | *Anticholinergics* | ≥ 65 | [25, 32, 34, 42, 43, 47, 48, 64, 66, 68, 88, 93] |
| | *Anticholinergics* | ≥ 75 | [87] |
| | *Trihexyphenidyl* | ≥ 75 | [87] |
| | *Tropatepine* | ≥ 75 | [87] |
| | *Biperiden* | ≥ 75 | [87] |
| **Delirium** | *Anticholinergics* | ≥ 65 | [25, 32, 34, 42, 84] |
| **Chronic constipation** | *Anticholinergics* | ≥ 65 | [25, 42, 48, 66, 84, 88] |
| | *Anticholinergics* | ≥ 75 | [87] |
| | *Anticholinergics* | - | [71] |
| **Glaucoma** | *Anticholinergics* | ≥ 75 | [87] |
| | *Anticholinergics* | ≥ 65 | [25, 42, 47, 66, 84, 88] |
| | *Medication with high anticholinergic activity* | | [107] |

| | | | |
|---|---|---|---|
| | *2 or more agents with low to moderate anticholinergic activity* | | [107] |
| | *Orphenadrine* [a] | | [71] |
| | *Hyoscine* [a] | | [71] |
| **History of urinary retention of BPH** | *Anticholinergics* | ≥ 65 | [25, 38, 42, 47, 48, 66, 84, 88] |
| | *Anticholinergics* | ≥ 75 | [87] |
| | *Anticholinergics* | - | [50] |
| | *Strongly anticholinergic drugs, except antimuscarinics for urinary incontinence* | ≥ 65 | [34] |
| **To treat extra-pyramidal side-effects of neuroleptic medications** | *Anticholinergics* | ≥ 65 | [25, 43, 47, 93] |
| **History of falls or fractures** | *Anticholinergics* | ≥ 65 | [84] |
| **Syncope** | *Anticholinergics* | ≥ 65 | [84] |
| **Postural hypotension** | *Anticholinergics* | ≥ 65 | [84] |

[a]. These medications were included because they can be used to treat some of the side effects caused by mental health medications.
BPH: Benign prostatic hyperplasia

**Table 14: PIM: Considering Diagnoses or Conditions (ADHD medications)**

| CONDITION | CLASS/MEDICATION | AGE | REFERENCE |
|---|---|---|---|
| **Anorexia** | *Cyproheptadine* | Children | [103] |
| | *Clonidine* | | [103] |
| **HTN** | *Clonidine* | - | [71] |
| | *Atomoxetine* | | [71] |
| **Palliative care patients with advanced dementia** | *Clonidine* | - | [80] |
| **Advanced dementia** | *Clonidine* | - | [86] |
| **Anorexia and malnutrition** | *Methylphenidate* | ≥ 65 | [47] |
| **to treat depression** | *Methylphenidate* | ≥ 65 | [47, 93] |
| **Epilepsy** | *Methylphenidate* | - | [71] |
| **Chronic constipation** | *Clonidine* | ≥ 75 | [87] |
| | *Guanfacine* | | [87] |
| **Insomnia** | *Amphetamine* | ≥ 65 | [34] |
| | *Methylphenidate* | | [34, 47, 84] |

HTN: Hypertension.

**Table 15: PIM: Considering Diagnoses or Conditions (non-mental health medications with mental health conditions)**

| CONDITION | CLASS/MEDICATION | AGE | REFERENCE |
|---|---|---|---|
| **Delirium** | *Corticosteroids* | ≥ 65 | [34] |
| | *Cimetidine* | | [34] |
| | *H2-receptor antagonists* | | [84] |
| | *Famotidine* | | [34] |
| | *Nizatidine* | | [34] |
| | *Ranitidine* | | [34] |
| | *Meperidine (Pethidine)* | | [34, 84] |
| **Insomnia** | *Pseudoephedrine* | ≥ 65 | [34, 47, 84] |
| | *Phenylephrine* | | [34, 47, 84] |
| | *Armodafinil* | | [34] |
| | *Modafinil* | | [34] |
| | *Theophylline* | | [34, 84] |
| | *Caffeine* | | [34, 84] |
| | *Phenylpropanolamine* | | [47] |
| | *PPI* | | [47] |
| **Depression** | *Methyldopa* | ≥ 65 | [116] |
| | *Sympatholytic antihypertensive* | | [38] |
| | *Moderate to high lipophilic beta-adrenergic blocking agent (e.g., propranolol, pindolol)* | | [38] |
| **Dementia** | *Statins* | ≥ 65 | [101] |
| | *Nimodipine* | | [101] |
| | *Pyritinol* | | [101] |
| | *Antioxidants: vitamin e* | | [101] |
| | *Antioxidants: vitamin c* | | [101] |
| | *Antioxidants: selenium* | | [101] |
| | *Phytotherapeutic agents, e.g. Ginseng* | | [101] |
| | *Hormone preparations, e.g. DHEA (Dehydroepiandrosterone), testosterone* | | [101] |
| | *Antiphlogistics, e.g. Indomethacin* | | [101] |
| | *Desferrioxamine* | | [101] |
| | *H2-receptor antagonists* | | [34, 84, 116] |
| | *Antispasmodic* | | [47, 93, 116] |

| | | | |
|---|---|---|---|
| | *Meperidine* | | [84] |
| **Advanced dementia** | *Colchicine* | - | [86] |
| | *Digoxin* | | [86] |
| | *Antiarrhythmics class I and III* | | [86] |
| | *Hydralazine* | | [86] |
| | *Bisphosphonates* | | [86] |
| | *Antiplatelets excluding aspirin* | | [86] |
| | *VKA* | | [86] |
| | *Anticoagulants excluding VKA* | | [86] |
| | *Appetite stimulants* | | [86] |
| | *Bladder relaxants* | | [86] |
| | *Antispasmodics* | | [86] |
| | *Lipid-lowering medications* | | [86] |
| | *Leukotriene receptor antagonists* | | [86] |
| | *Antioestrogens* | | [86] |
| | *Sex hormones* | | [86] |
| | *Cytotoxic chemotherapy* | | [86] |
| | *Hormone antagonists* | | [86] |
| | *Immunomodulators* | | [86] |
| | *NSAIDs* | | [86] |
| | *Antidiarrheals* | | [86] |
| | *Laxatives* | | [86] |
| | *Antiemetics* | | [86] |
| | *Proton pump inhibitors* | | [86] |
| | *Beta-blockers* | | [86] |
| | *Calcium channel blockers* | | [86] |
| | *Diuretics* | | [86] |
| | *Angiotensin-converting enzyme inhibitors and angiotensin receptor blockers* | | [86] |
| | *Nitrates/nitroglycerin* | | [86] |
| | *Antibacterials* | | [86] |
| | *Antivirals* | | [86] |
| | *Antiparasitic agents* | | [86] |
| | *Oral hypoglycaemics* | | [86] |
| | *Thyroid hormones* | | [86] |
| | *Antithyroid medications* | | [86] |
| | *Corticosteroids* | | [86] |
| | *Insulin* | | [86] |
| | *Antihistamine second generation* | | [86] |
| | *Electrolytes* | | [86] |
| | *Antiglaucoma drops* | | [86] |
| | *Anti-inflammatory eye drops* | | [86] |
| | *Allopurinol* | | [86] |
| | *Uroselective alpha blockers* | | [86] |
| | *Aspirin* | | [86] |
| **Advanced dementia (palliative)** | *Bisphosphonates* | - | [80] |
| | *Hydralazine* | | [80] |
| | *Antiarrhythmics* | | [80] |
| | *Heparin and LMWH* | | [80] |
| | *Antispasmodics* | | [80] |
| | *Warfarin* | | [80] |
| | *Hormone antagonists* | | [80] |
| | *Immunomodulators* | | [80] |
| | *Sex hormones* | | [80] |
| | *Antioestrogens* | | [80] |
| | *Lipid-lowering medications* | | [80] |
| | *Antiplatelets excluding aspirin* | | [80] |
| | *Leukotriene receptor antagonists* | | [80] |
| | *Cytotoxic chemotherapy* | | [80] |
| | *Mineralocorticoids* | | [80] |
| | *Tamsulosin* | | [80] |
| | *Digoxin* | | [80] |
| | *Bladder relaxants* | | [80] |
| | *Alpha blockers* | | [80] |
| | *Antiandrogens* | | [80] |
| | *Appetite stimulants* | | [80] |
| | *Proton pump inhibitors* | | [80] |
| | *Histamine-2 receptor blockers* | | [80] |
| | *Beta-blockers* | | [80] |
| | *Calcium channel blockers* | | [80] |
| | *Diuretics* | | [80] |

| | | | |
|---|---|---|---|
| | *ACEi and ARB* | | [80] |
| | *Nitroglycerin* | | [80] |
| | *Mucolytics* | | [80] |
| | *Inhaled corticosteroids* | | [80] |
| | *Antibacterials* | | [80] |
| | *Antivirals* | | [80] |
| | *Antiparasitic agents* | | [80] |
| | *Antifungal creams* | | [80] |
| | *Oral hypoglycaemics* | | [80] |
| | *Thyroid hormones* | | [80] |
| | *Antithyroid medications* | | [80] |
| | *Corticosteroids* | | [80] |
| | *Insulin* | | [80] |
| | *Antihistamines* | | [80] |
| | *Decongestants* | | [80] |
| | *Electrolytes* | | [80] |
| | *Nutritional supplements* | | [80] |
| | *Antiglaucoma drops* | | [80] |
| | *Anti-inflammatory eye drops* | | [80] |
| | *Capsaicin* | | [80] |
| | *Allopurinol* | | [80] |
| | *Colchicine* | | [80] |
| **Dementia (non-palliative)** | *Fentanyl* | ≥ 65 | [47] |
| | *Morphine* | | [47] |

ACEi: Angiotensin-converting-enzyme inhibitor. ARB: Angiotensin II receptor blockers. LMWH: Low-molecular-weight heparin. NSAID: Nonsteroidal anti-inflammatory drugs. PPI: Proton pump inhibitors. VKA: Vitamin K antagonists.

**Table 16: PIM: Considering Diagnoses or Conditions (Non-specific psychotropics)**

| CONDITION | CLASS/MEDICATION | AGE | REFERENCE |
|---|---|---|---|
| **History of falls** | *psychotropics* | ≥ 65 | [64] |

## Drug-Drug Interactions

Prescribing [Class/Medication] with [Class/Medication] +/- to a patient aged [Age]

**Table 17: Drug-Drug Interactions (Non-specific psychotropics)**

| Medication/Class | Medication/Class | Age | References |
|---|---|---|---|
| *More than one psychotropic drug from the same class* | | ≥ 75 | [87] |
| *3 or more psychotropics* | | ≥ 70 | [95, 96] |
| *Multiple psychotropics* | | ≥ 65 | [107, 108] |
| *≥2 CNS-active drugs* | *Opioid receptor agonist* | ≥ 65 | [34] |
| *Tranquilizer* | *Tranquilizer* | ≥ 65 | [112] |

CNS: Central nervous system

**Table 18: Drug-Drug Interactions (Antipsychotics)**

| Medication/Class | Medication/Class | Age | References |
|---|---|---|---|
| *3 or more antipsychotics* | | adults | [69] |
| *Antipsychotics* | *≥2 CNS-active drugs* | ≥ 65 | [34] |
| | *Antipsychotic* | ≥ 65 | [32, 41] |
| | | - | [22, 71] |
| | *Antiparkinsonian agents* | ≥ 65 | [44] |
| *Atypical antipsychotic* | *Atypical antipsychotic* | - | [22] |
| *Pimozide* | *Macrolides antibiotics* | - | [89] |
| | *Azole antifungal* | | [89] |
| *Phenothiazine antipsychotics* | *Antiparkinsonian agents* | ≥ 65 | [108] |
| *Aripiprazole* | *Anti-HCV antivirals* | - | [71] |
| *Quetiapine* | | | [71] |
| *Iloperidone* | | | [71] |
| *Quetiapine* | *CYP inhibitor* | - | [104] |

CNS: Central nervous system. HCV: Hepatitis C virus

**Table 19: Drug-Drug Interactions (Antidepressants)**

| Medication/Class | Medication/Class | Age | References |
|---|---|---|---|
| *Antidepressants* | *Antidepressants* | ≥ 65 | [32] |
| | *≥2 other CNS-active drugs* | | [34] |
| *Any serotonergic drug (SSRI, SNRI, MAO-I, TCA, triptans)* | *Any serotonergic drug (SSRI, SNRI, MAO-I, TCA, triptans)* | - | [104] |

| | | | |
|---|---|---|---|
| *TCA* | *MAO* | - | [15] |
| | *Opiate* | ≥ 65 | [43, 47, 68] |
| | *Calcium channel blocker* | ≥ 65 | [43, 47, 68] |
| | *TCA* | ≥ 65 | [41, 112] |
| | *Clonidine* | ≥ 65 | [41] |
| | | - | [111] |
| | *Cimetidine* | ≥ 65 | [116] |
| | | - | [111] |
| | *Fluoxetine* | - | [111] |
| | | ≥ 70 | [96] |
| | *Fluvoxamine* | ≥ 70 | [96] |
| | *Paroxetine* | - | [111] |
| | *Selegiline* | - | [102] |
| *SSRI* | *Tramadol* | ≥ 70 | [95] |
| | | - | [15] |
| | *Aspirin (no protection)* | ≥ 65 | [92] |
| | | - | [15] |
| | *medications that may contribute to serotonin toxicity* | ≥ 65 | [64] |
| | *MAOI* | ≥ 65 | [93] |
| | | - | [89] |
| | *SSRI's* | ≥ 65 | [25, 43, 68] |
| | *NSAID* | ≥ 65 | [48, 68] |
| | *NSAID (no protection)* | ≥ 45 | [70] |
| | *Venlafaxine.* | ≥ 45 | [70] |
| | *Vitamin K antagonists* | - | [71] |
| | *Selegiline* | - | [111] |
| *SSRIs/SNRIs* | *NSAIDs* | ≥ 70 | [95, 96] |
| | *Warfarin* | | [95, 96] |
| *MAOI* | *Tramadol* | - | [15] |
| | *Dextromethorphan* | - | [89] |
| | *Anorexiants* | - | [89] |
| | *Amphetamine and derivatives* | - | [102] |
| | *Fluoxetine* | - | [102] |
| | *Narcotic analgesics* | - | [102] |
| | *Triptans* | - | [102] |
| | *Sympathomimetics* | - | [89] |
| | *Meperidine* | - | [89] |
| | *Bupropion* | - | [104] |
| | *Levodopa* | - | [104] |
| | *Levodopa* | ≥ 65 | [41, 112] |
| | *Meperidine* | ≥ 65 | [41, 112] |
| | *Antidepressants* | ≥ 65 | [41, 112] |
| | *MAOI* | ≥ 65 | [41, 112] |
| *Amitriptyline* | *Sertraline* | children | [79] |
| | *Trazodone* | | [79] |
| | *Psycholeptic* | ≥ 65 | [41] |
| | *opiate* | ≥ 65 | [83] |
| | *Calcium channel blocker* | ≥ 65 | [83] |
| *Citalopram* | *QT-prolonging drugs* | - | [15] |
| *Citalopram* | *Linezolid* | children | [79] |
| *Sertraline* | | | [79] |
| *Fluoxetine* | *Alprazolam* | - | [111] |
| *Fluvoxamine* | *Theophylines* | - | [89] |
| | *Ramelteon* | - | [102] |
| *Paroxetine* | *Metoprolol* | ≥ 70 | [95] |
| *Fluoxetine* | | | [95] |
| *Bupropion* | | | [95] |
| *Trazodone* | *anti-HCV antivirals* | - | [71] |
| *Escitalopram* | | | [71] |
| *Tranylcypromine* | *Procarbazine* | - | [102] |
| *Clomipramine* | *Opiate* | ≥ 65 | [83] |
| | *Calcium channel blocker* | | [83] |
| *Imipramine* | *Opiate* | ≥ 65 | [83] |
| | *Calcium channel blocker* | | [83] |

CNS: Central nervous system. MAOi: Monoamine oxidase inhibitor. NSAID: Nonsteroidal anti-inflammatory drugs. SNRI: Serotonin and norepinephrine reuptake inhibitors. SSRI: Selective serotonin reuptake inhibitor. TCA: Tricyclic antidepressants.

**Table 20: Drug-Drug Interactions (Sedative, hypnotics and anxiolytics)**

| Medication/Class | Medication/Class | Age | References |
|---|---|---|---|
| *Hypnotic or sedative* | *Hypnotic or sedative* | ≥ 65 | [32, 41, 112] |
| *Benzodiazepine* | *Hypnotic or sedative* | ≥ 65 | [41, 112] |
| *Benzodiazepine* | *≥2 CNS-active drugs* | ≥ 65 | [34] |
| *Benzodiazepine* | *Azole antifungal agents* | - | [89] |
| *Benzodiazepine* | *Cimetidine* | ≥ 65 | [116] |
| *Benzodiazepine* | *Benzodiazepines* | ≥ 45 | [70] |
| *Benzodiazepine* | *Benzodiazepines* | ≥ 65 | [41, 74, 108, 112, 114] |
| *Benzodiazepine* | *Benzodiazepines* | - | [71] |
| *Alprazolam* | *Strong CYP3A4 inhibitor* | ≥ 65 | [48] |
| *Midazolam* | *Strong CYP3A4 inhibitor* | ≥ 65 | [48] |
| *Triazolam* | *Strong CYP3A4 inhibitor* | ≥ 65 | [48] |
| *Midazolam* | *Protease inhibitors* | - | [104] |
| *Triazolam* | *Protease inhibitors* | - | [104] |
| *Clonazepam* | *Clonazepam* | ≥ 65 | [112] |
| *Clonazepam* | *Benzodiazepines* | ≥ 65 | [41, 112] |
| *Clorazepate* | *Acepromazine* | ≥ 65 | [46] |
| *Flurazepam* | *Anti-HCV antivirals* | - | [71] |
| *Guazepam* | *Anti-HCV antivirals* | - | [71] |
| *Triazolam* | *Anti-HCV antivirals* | - | [71] |
| *Alprazolam* | *Anti-HCV antivirals* | - | [71] |
| *Z-drugs* | *≥2 CNS-active drugs* | ≥ 65 | [34] |
| *Zolpidem* | *Strong CYP3A4 inhibitor* | ≥ 65 | [48] |
| *Zopiclone* | *Strong CYP3A4 inhibitor* | ≥ 65 | [48] |
| *Zolpidem* | *Anti-HCV antivirals* | - | [71] |
| *Barbiturates* | *Hypnotic or sedative* | ≥ 65 | [41, 112] |
| *Barbiturates* | *Hormonal contraceptive or combination pills* | - | [111] |
| *Barbiturates* | *Steroids* | - | [111] |
| *Barbiturates* | *Barbiturates* | ≥ 65 | [41, 112] |
| *Barbiturates* | *Warfarin* | - | [89, 111] |
| *Barbiturates* | *Opioids* | ≥ 65 | [41, 112] |
| *Barbiturates* | *Antidepressants* | ≥ 65 | [41, 112] |
| *Phenobarbital* | *Rivaroxaban* | - | [71] |
| *Phenobarbital* | *Voriconazole* | children | [79] |
| *Propranolol* | *Insulin* | - | [111] |
| *Propranolol* | *Rifampin* | - | [111] |
| *Propranolol* | *Verapamil* | - | [111] |

CNS: Central nervous system. CYP: Cytochrome P450. HCV: Hepatitis C virus.

**Table 21: Drug-Drug Interactions (mood stabilisers)**

| Medication/Class | Medication/Class | Age | References |
|---|---|---|---|
| *Valproic acid* | *Lamotrigine* | Children | [79] |
| *Valproic acid* | *Meropenem* | Children | [79] |
| *Valproic acid* | *Barbiturates* | - | [111] |
| *Valproic acid* | *Carbapenem* | - | [104] |
| *Carbamazepine* | *Clarithromycin* | - | [109] |
| *Carbamazepine* | *Clarithromycin* | ≥ 45 | [70] |
| *Carbamazepine* | *Clarithromycin* | ≥ 70 | [96] |
| *Carbamazepine* | *Erythromycin* | ≥ 45 | [70] |
| *Carbamazepine* | *Erythromycin* | ≥ 70 | [96] |
| *Carbamazepine* | *Cimetidine* | ≥ 65 | [116] |
| *Carbamazepine* | *oral or intravaginal contraceptives, patches or pure progestogen pills* | - | [71] |
| *Carbamazepine* | *Warfarin* | - | [111] |
| *Carbamazepine* | *Propoxyphene* | - | [89, 111] |
| *Carbamazepine* | *Rivaroxaban* | - | [71] |
| *Lithium* | *ACEi* | ≥ 65 | [34] |
| *Lithium* | *Loop diuretics* | ≥ 65 | [34] |
| *Lithium* | *thiazide diuretic* | - | [10, 39, 41, 111] |
| *Lithium* | *RAAS inhibitors* | ≥ 65 | [48] |
| *Lithium* | *NSAID* | ≥ 65 | [48] |
| *Lithium* | *Diuretics* | ≥ 65 | [48] |
| *Lithium* | *Diuretics* | - | [49] |
| *Lamotrigine* | *Hormonal contraceptive or combination pills* | - | [71] |

ACEi: Angiotensin-converting-enzyme inhibitor. NSAID: Nonsteroidal anti-inflammatory drugs. RAAS: Renin–angiotensin system.

**Table 22: Drug-Drug Interactions (Anti-dementia)**

| Medication/Class | Medication/Class | Age | References |
|---|---|---|---|

| | | | |
|---|---|---|---|
| *Anticholinesterase drugs* | *Anticholinergic* | ≥ 75 | [87] |
| | *Anticholinergic* | ≥ 65 | [77] |
| | *Anticholinesterase drugs* | - | [71] |
| | *Beta-blockers* | ≥ 65 | [25] |
| | *Digoxin* | ≥ 65 | [25] |
| | *Diltiazem* | ≥ 65 | [25] |
| | *Verapamil* | ≥ 65 | [25] |

**Table 23: Drug-Drug Interactions (Anticholinergics)**

| Medication/Class | Medication/Class | Age | References |
|---|---|---|---|
| *Two or more agents with low to moderate anticholinergic activity* | | ≥ 65 | [107] |
| *Anticholinergic* | *Anticholinergic* | ≥ 65 | [25, 32, 34, 48] |

**Table 24: Drug-Drug Interactions (ADHD medications)**

| Medication/Class | Medication/Class | Age | References |
|---|---|---|---|
| *Clonidine* | *Propranolol* | - | [111] |

## Inappropriate Duration

Prescribing [Class/Medication] for [Duration] +/- to a patient with [Condition] +/- and aged [Age]

**Table 25: Inappropriate Duration (Antipsychotics)**

| Class/Medication | Condition | Duration | Age | References |
|---|---|---|---|---|
| *Antipsychotics* | Dementia but not psychosis | >6 weeks | ≥ 65 | [10] |
| | Parkinsonism | >1 month | - | [15] |
| | | | ≥ 65 | [43, 47] |
| | as long-term hypnotics | >1 month | ≥ 65 | [43, 47, 77] |
| | non-psychotic indications | long term | ≥ 65 | [108] |
| | - | >1 month | ≥ 65 | [48] |
| | - | long term | ≥ 65 | [42] |
| *More than one Antipsychotics* | - | >2 month | Adults | [69] |
| | | 45 days | 6–17 | [69] |
| *Risperidone* | - | >6 weeks | ≥ 65 | [46] |
| | dementia and psychosis | ≥ 12 weeks | ≥ 65 | [73] |
| *Perphenazine* | Parkinsonism | >1 month | ≥ 65 | [83] |
| | as long-term hypnotics | >1 month | | [83] |
| *Clozapine* | Parkinsonism | >1 month | ≥ 65 | [83] |
| | as long-term hypnotics | >1 month | | [83] |
| *Haloperidol* | Parkinsonism | >1 month | ≥ 65 | [83] |
| | as long-term hypnotics | >1 month | | [83] |
| *Olanzapine* | Parkinsonism | >1 month | ≥ 65 | [83] |
| | as long-term hypnotics | >1 month | | [83] |

**Table 26: Inappropriate Duration (Antidepressants)**

| Class/Medication | Condition | Duration | Age | References |
|---|---|---|---|---|
| *Antidepressants* | - | long term | ≥ 65 | [42] |
| | | ≥ 1 year | ≥ 65 | [48] |
| *Three or more Antidepressants* | - | >3 month | adults | [69] |
| *TCA* | - | >1 month | ≥ 65 | [73] |
| *More than one TCA* | - | >1 month | adults | [69] |
| *SSRI* | - | < 4 weeks (too short) | ≥ 65 | [92] |
| | single episode of depression | > 6 months | ≥ 65 | [92] |
| *More than one SSRI* | - | >2 month | adults | [69] |
| *SSRI and SNRI combination* | - | >2 month | adults | [69] |

SNRI: Serotonin and norepinephrine reuptake inhibitors. SSRI: Selective serotonin reuptake inhibitor. TCA: Tricyclic antidepressants.

**Table 27: Inappropriate Duration (Sedative, hypnotics and anxiolytics)**

| Class/Medication | Condition | Duration | Age | References |
|---|---|---|---|---|
| *Hypnotics* | - | long term | - | [71] |
| | | | ≥ 65 | [92] |
| | | | ≥ 70 | [95] |
| | | >1 month | - | [115] |
| *Benzodiazepine* | not receiving on a long-term basis | ≥21 days | ≥ 65 | [10] |
| | Depression | ≥21 days | ≥ 65 | [10] |
| | | >1 month | - | [15] |
| | - | >1 month | ≥ 65 | [25, 32, 64, 112] |
| | | | - | [15, 99] |
| | | | ≥ 45 | [70] |

| | | | | |
|---|---|---|---|---|
| | | long term | ≥ 65 | [42, 92] |
| | | >6 month | ≥ 65 | [75] |
| *Long-acting Benzodiazepine* | - | >1 month | ≥ 65 | [43, 68, 77] |
| *Long-acting Benzodiazepine* | Agitation in dementia | long term | ≥ 65 | [93] |
| *Long-acting Benzodiazepine* | Anxiety | long term | ≥ 65 | [93] |
| *Long-acting Benzodiazepine* | Insomnia | long term | ≥ 65 | [93] |
| *Intermediate acting benzodiazepine* | - | >1 month | ≥ 65 | [48] |
| *Short acting benzodiazepine* | - | >1 month | ≥ 65 | [41, 73] |
| *Alprazolam* | - | >1 month | ≥ 65 | [41] |
| *Oxazepam* | - | >1 month | ≥ 65 | [41, 47] |
| *Triazolam* | - | >1 month | ≥ 65 | [41] |
| *Triazolam* | to treat insomnia | long term | ≥ 65 | [93] |
| *Chlorazepate* | - | >1 month | ≥ 65 | [83] |
| *Chlordiazepoxide* | - | >1 month | ≥ 65 | [83] |
| *Diazepam* | - | >1 month | ≥ 65 | [83] |
| *Flurazepam* | - | >1 month | ≥ 65 | [83] |
| *Z-drugs* | not receiving on a long-term basis | ≥21 days | ≥ 65 | [10] |
| *Z-drugs* | Depression | ≥21 days | ≥ 65 | [10] |
| *Z-drugs* | Depression | >1 month | - | [15] |
| *Z-drugs* | - | >1 month | - | [15] |
| *Z-drugs* | - | >1 month | ≥ 45 | [70] |
| *Z-drugs* | - | >1 month | ≥ 65 | [48, 73] |
| *Z-drugs* | - | long term | ≥ 65 | [42] |
| *Barbiturates* | to treat insomnia | long term | ≥ 65 | [93] |
| *Phenobarbital* | - | long term | ≥ 65 | [47] |
| *First-generation antihistamine* | - | > 1 week | ≥ 65 | [43, 47] |
| *Chlorpheniramine* | - | > 1 week | ≥ 65 | [83] |
| *Clemastine* | - | > 1 week | ≥ 65 | [83] |
| *Doxylamine* | - | > 1 week | ≥ 65 | [83] |
| *Triprolidine* | - | > 1 week | ≥ 65 | [83] |

**Table 28: Inappropriate Duration (Non-specific psychotropics)**

| Class/Medication | Condition | Duration | Age | References |
|---|---|---|---|---|
| *Four or more Psychotropics* | - | >3 months | 6–17 | [69] |

**Table 29: Inappropriate Duration (non-mental health medication with mental health condition)**

| Class/Medication | Condition | Duration | Age | References |
|---|---|---|---|---|
| *Opioids* | Dementia (non-palliative) | long term | ≥ 65 | [43] |

**Table 30: Inappropriate Duration (Anticholinergics)**

| Class/Medication | Condition | Duration | Age | References |
|---|---|---|---|---|
| *Belladonna alkaloids* [a] | - | >3 months | ≥ 65 | [41] |
| *Clidinium-chlordiazepoxide* [a] | - | >3 months | ≥ 65 | [41] |
| *Dicyclomine* [a] | - | >3 months | ≥ 65 | [41] |
| *Propantheline* [a] | - | >3 months | ≥ 65 | [41] |

[a]. These medications were included because they can be used to treat some of the side effects caused by mental health medications.

## Inappropriate dose

Prescribing [Medication] at a dose [Dose] +/- to a patient with [Condition] +/- and aged [Age]

**Table 31: Inappropriate dose (Antipsychotics)**

| Medication (dose) | Condition | Age | References |
|---|---|---|---|
| *Aripiprazole (2-15 mg/day)* | BPSD: paranoia, hallucination | ≥ 65 | [101] |
| *Clozapine (10-50 mg/day)* | BPSD: paranoia, hallucination | ≥ 65 | [101] |
| *Haloperidol (>2 mg)* | | ≥ 65 | [45-47] |
| *Haloperidol (>3 mg/day)* | | ≥ 65 | [41] |
| *Haloperidol (>5 mg/day)* | | ≥ 65 | [46] |
| *Haloperidol (initially 0.5 mg/day, max. 3 mg/day)* | BPSD: paranoia, hallucination | ≥ 65 | [101] |
| *Melperone (25-150 mg/day)* | BPSD: paranoia, hallucination | ≥ 65 | [101] |
| *Melperone (25-150 mg/day)* | BPSD: restlessness, agitation | ≥ 65 | [101] |
| *Olanzapine (>10 mg)* | | ≥ 65 | [45, 46, 77] |
| *Pipamperone (20-120 mg/day)* | BPSD: restlessness, agitation | ≥ 65 | [101] |
| *Quetiapine (25-200 mg/day)* | BPSD: paranoia, hallucination | ≥ 65 | [101] |
| *Quetiapine (25-200 mg/day)* | BPSD: restlessness, agitation | ≥ 65 | [101] |
| *Reserpine (>0.1 mg/day)* | | ≥ 65 | [34] |

| | | | |
|---|---|---|---|
| *Risperidone (initially 0,5-1 mg/day)* | BPSD: paranoia, hallucination | ≥ 65 | [101] |
| *Risperidone (initially 0,5-1 mg/day, Maximum 3 mg/day)* | BPSD: restlessness, agitation | ≥ 65 | [101] |
| *Risperidone (1 mg BID)* | Dementia and agitation | ≥ 75 | [107] |
| *Thioridazine (>30mg/day)* | | ≥ 65 | [41] |
| ***High dose antipsychotics*** ***total daily dose is above the maximum recommended by the British National Formulary (BNF).*** | | - | [22] |

BID: twice a day. BPSD: Behavioural and Psychological Symptoms of Dementia

**Table 32: Inappropriate dose (Antidepressants)**

| **Medication** (dose) | **Condition** | **Age** | **References** |
|---|---|---|---|
| *Citalopram (10-30mg)* | BPSD: restlessness, agitation | ≥ 65 | [101] |
| *Doxepin (>6 mg/day)* | | ≥ 65 | [34, 84] |
| *Doxepin (25-50 mg)* | BPSD: sleep disorders | ≥ 65 | [101] |
| *Fluoxetine (>40 mg/day)* | | ≥ 65 | [41] |
| *Imipramine (>100 mg/day)* | | ≥ 65 | [41] |
| *Trazodone (50-200 mg/day)* | BPSD: restlessness, agitation | ≥ 65 | [101] |
| *Trimipramine (>100 mg/day)* | | ≥ 65 | [41] |
| *Mirtazapine (15-45mg/day)* | BPSD: depression | ≥ 65 | [101] |
| *Mirtazapine (15-30mg/day)* | BPSD: sleep disorders | ≥ 65 | [101] |

**Table 33: Inappropriate dose (Mood stabilisers)**

| **Medication** (dose) | **Age** | **References** |
|---|---|---|
| *Valproate (<1 g/day)* | - | [22] |
| *Carbamazepine (< 600 mg/day)* | - | [22] |

**Table 34: Inappropriate dose (ADHD medications)**

| **Medication** (dose) | **Age** | **References** |
|---|---|---|
| *SR Methylphenidate two doses per day, rather than one dose* | Children | [103] |

SR: sustained release

**Table 35: Inappropriate dose (Sedatives, hypnotics and anxiolytics)**

| **Medication** (dose) | **Condition** | **Age** | **References** |
|---|---|---|---|
| *Alprazolam (>2 mg/day)* | | ≥ 65 | [39, 47, 68] |
| *Alprazolam (>2 mg/day)* | | ≥ 75 | [87] |
| *Alprazolam (>0.75 mg/day)* | | ≥ 65 | [41] |
| *Bromazepam (> 1,5 mg)* | | ≥ 65 | [47] |
| *Brotizolam (>0.125 mg/day)* | | ≥ 65 | [45, 46] |
| *Clomethiazole (5-15 mg/day)* | BPSD: restlessness, agitation | ≥ 65 | [101] |
| *Clotiazepam (>5 mg/day)* | | ≥ 75 | [87] |
| *Clotiazepam (>5 mg/day)* | | ≥ 65 | [46] |
| *Gabapentin (>1400mg/day)* | CrCl 30-59 mL/min | - | [72] |
| *Gabapentin (>700mg/day)* | CrCl 15-29 mL/min | - | [72] |
| *Gabapentin (>300mg/day)* | CrCl 10-14 mL/min | - | [72] |
| *Gabapentin (>150mg/day)* | CrCl < 10 mL/min | - | [72] |
| *Loprazolam (>0,5 mg/day)* | | ≥ 75 | [87] |
| *Loprazolam (>0.5 mg/day)* | | ≥ 65 | [46] |
| *Lorazepam (>1 mg/day)* | | ≥ 65 | [46] |
| *Lorazepam (> 2 mg/day)* | | ≥ 65 | [45] |
| *Lorazepam (>3 mg/day)* | | ≥ 75 | [87] |
| *Lorazepam (>3 mg/day)* | | ≥ 65 | [39, 41, 68] |
| *Lormetazepam (>0.5 mg/day)* | | ≥ 75 | [87] |
| *Lormetazepam (>0.5 mg/day)* | | ≥ 65 | [45, 46] |
| *Melatonin SR (2-4 mg)* | BPSD: sleep disorders | ≥ 65 | [101] |
| *Oxazepam (>30 mg/day)* | | ≥ 70 | [95, 96] |
| *Oxazepam (>30 mg/day)* | | ≥ 65 | [47] |
| *Oxazepam (>60 mg/day)* | | ≥ 65 | [39, 45, 46, 68] |
| *Oxazepam (>60 mg/day)* | | ≥ 75 | [87] |
| *Oxazepam unit dose >30 mg* | | ≥ 65 | [41] |
| *Pregabalin (>300mg/day)* | CrCl 30-59 mL/min | - | [72] |
| *Pregabalin (>150mg/day)* | CrCl 15-29 mL/min | - | [72] |
| *Pregabalin (>75mg/day)* | CrCl < 15 mL/min | - | [72] |
| *Temazepam (>15 mg/day)* | | ≥ 65 | [39, 68] |
| *Temazepam (>15 mg/day)* | | ≥ 75 | [87] |
| *Temazepam (>30 mg/day)* | | ≥ 65 | [41] |
| *Triazolam (>0.25 mg/day)* | | ≥ 65 | [39, 66, 68] |
| *Triazolam (>0.25 mg unit dose)* | | ≥ 65 | [41] |
| *Triazolam (>0.25 mg/day)* | | ≥ 75 | [87] |
| *Triazolam (>0.125 mg/day)* | | ≥ 65 | [41] |
| *Zaleplon (>5 mg/day)* | | ≥ 65 | [45, 46] |

| | | | |
|---|---|---|---|
| *Zolpidem (>5 mg/day)* | | ≥ 65 | [45-47] |
| *Zolpidem (>5 mg/day)* | | ≥ 75 | [87] |
| *Zopiclone (>3.75 mg/day)* | | ≥ 75 | [87] |
| *Zopiclone (>3.75 mg/day)* | | ≥ 65 | [45-47] |
| *Zopiclone (>5 mg/day)* | | ≥ 70 | [95] |
| *Zopiclone (>7.5mg/day)* | | ≥ 70 | [96] |
| *Zopiclone (3,75-7,5 mg)* | BPSD: sleep disorders | ≥ 65 | [101] |

BPSD: Behavioural and Psychological Symptoms of Dementia. CrCl: Creatinine Clearance

## Monitoring

Prescribing [Class/Medication] with monitoring [Test] [Frequency]

**Table 36: Monitoring (Antipsychotics)**

| Medication/Class | Test | Age | Frequency | References |
|---|---|---|---|---|
| *Antipsychotics* | Glucose | - | Annually | [39] |
| | | | 3-4 months after starting therapy | [49] |
| | Weight | - | Annual | [39] |
| | | | 3-4 months after starting therapy | [49] |
| | Lipid profile | - | 3 months after starting therapy | [49] |
| *Clozapine* | WBC | ≥ 65 | NR | [92] |
| | | - | NR | [117] |

**Table 37: Monitoring (mood stabilisers)**

| Medication/Class | Test | Age | Frequency | References |
|---|---|---|---|---|
| *Carbamazepine* | AST, ALT | - | Baseline and annually | [113] |
| | | | Annually | [105] |
| | LFT | - | Annually | [81] |
| | FBC | - | Baseline, monthly for 3 months, and annually | [113] |
| | | | Annual | [81, 105] |
| | | | Baseline and periodically | [50] |
| | | ≥ 65 | Weekly during the first month of therapy, at least monthly during the next 5 months of therapy, and at least every 6 months thereafter | [38] |
| | Carbamazepine level | - | Annually | [81, 105] |
| | | ≥ 65 | Every 6 months | [38] |
| | | - | 2-4 weeks after initiation, with changing clinical status, and annually | [113] |
| | | - | Every 6 months | [50] |
| *Valproate* | LFT | - | Every 3 months | [61] |
| | | | Annually | [81] |
| | | | First 6 months of therapy | [49] |
| | AST of ALT | - | Baseline, every 2 months for 6 months, and annually | [113] |
| | FBC | - | Annually | [81, 113] |
| | | | First 6 months of therapy | [49] |
| | Valproate level | - | Annually | [81] |
| | | - | At 2-4 weeks After initiation, with changing clinical status, and annually | [113] |
| | | ≥ 65 | Every 6 months | [38] |
| | | - | Every 6 months | [49, 50] |
| *Lithium* | lithium level | - | Annually | [81, 105] |
| | | - | Every 3 months | [17, 61] |
| | | - | Every 6 months | [10, 49, 50] |
| | | ≥ 65 | Every 3 months | [38] |
| | | ≥ 65 | Every month | [38] |
| | | ≥ 65 | NR | [92] |
| | | - | 2-4 weeks after initiation, with changing clinical status, and annually | [113] |
| | TFT | - | Annually | [61, 81] |
| | | ≥ 65 | NR | [92] |
| | | - | Every 6 months | [49] |
| | TSH | - | Baseline, 3 and 6 month and annually | [113] |
| | | ≥ 65 | Every 6 months | [38] |
| | Ca and Mg | ≥ 65 | NR | [92] |
| | Na and $K^+$ | - | Annually | [81] |
| | FBC | - | Baseline, 1 month after stabilized, and annually | [113] |
| | | | Annually | [105] |
| | Creatinine | - | Baseline, 1 month after stabilized, and annually | [113] |
| | | - | Annual | [81, 105] |

| | | | | |
|---|---|---|---|---|
| | | ≥ 65 | Every 3 months | [38] |
| | Renal function | ≥ 65 | NR | [92] |
| | Urinalysis | - | Annually | [81] |

ALT: Alanine transaminase. AST: Aspartate transaminase. Ca: Calcium. FBC: Full blood count. $K^+$: Potassium. Na: Sodium. LFT: Liver function test. NR: Not reported. TFT: Thyroid function test. TSH: Thyroid stimulating hormone.

**Table 38: Monitoring (ADHD medications)**

| Medication/Class | Test | Age | Frequency | References |
|---|---|---|---|---|
| *Methylphenidate* | Growth chart (height and weight) | Children | NR | [103] |

NR: not reported

**Table 39: Monitoring (Sedative, hypnotics and anxiolytics)**

| Medication/Class | Test | Age | Frequency | References |
|---|---|---|---|---|
| *Phenobarbital* | AST of ALT | - | at Baseline and every 6 months | [113] |
| | CBC | - | at Baseline and every 6 months | [113] |
| | Phenobarbital Level | - | at 2-4 week After initiation, with changing clinical status, and yearly | [113] |

## Omission

Patient with [Condition +/- Age] NOT prescribed [Class/Medication]

**Table 40: Omission**

| Medication/Class | Condition | Age | References |
|---|---|---|---|
| *Acetylcholinesterase inhibitor* | Mild- moderate Alzheimer's dementia | ≥ 65 | [25] |
| | Lewy Body dementia | ≥ 65 | [25] |
| *Antidepressants* | Moderate/severe depressive symptoms lasting at least three months | ≥ 65 | [43] |
| *Non-TCA Antidepressants* | Major depressive symptoms. | ≥ 65 | [25] |
| *SSRI* | Persistent severe anxiety that interferes with independent functioning. | ≥ 65 | [25] |
| *SSRI first line* | Depression | - | [71] |
| *Mood stabilisers* | on antidepressants for acute bipolar depression | Adult | [49] |
| *Lithium OR Valproate OR Carbamazepine* | on lamotrigine and SSRI in bipolar disorder | Adult | [49] |

SSRI: Selective serotonin reuptake inhibitor. TCA: Tricyclic antidepressants.

## Other inappropriate prescribing indicators

**Table 41: Other inappropriate prescribing indicators**

| Class | Indicator | Age | References |
|---|---|---|---|
| ***Antidepressants*** | TCA prescribed as first-line treatment of depression. | ≥ 65 | [25] |
| | TCA prescribed as first-line treatment of depression. | ≥ 45 | [70] |
| | Continued treatment for depression in spite of lacking indication. | ≥ 65 | [92] |
| | Discontinuation of antidepressant, which leads to withdrawal symptoms | ≥ 65 | [92] |
| | TCA in combination with anticholinergic agents in patient with nocturnal enuresis | children | [103] |
| | TCA prescribed as a first-line treatment with nocturnal enuresis | children | [103] |
| | TCA except in case of severe depression or in low dose for neuropathic pain | ≥ 65 | [42] |
| | Patient diagnosed with acute bipolar depression is prescribed antidepressant monotherapy | Adult | [49] |
| ***Antipsychotics*** | Risperidone continued following discharge without follow-up to a patient with dementia | ≥ 75 | [107] |
| | Phenothiazines as first-line treatment | ≥ 65 | [25] |
| | Neuroleptics as hypnotics, unless sleep disorder is due to psychosis or dementia | ≥ 65 | [25] |
| | Prescribing older antipsychotic to a patient with Parkinsonian and mild cognitive impairment and mild to moderate agitation in the evening | ≥ 75 | [107] |
| ***Mood stabilisers*** | Lithium dose not adjusted or omitted in a patient with a lithium concentration above the therapeutic range (>1. - mmol l-1) | - | [15] |
| | Lithium prescribed in conjunction with newly prescribed nonsteroidal anti-inflammatory drugs without dose adjustment or increased monitoring | - | [15] |
| | Lithium therapy prescribed in conjunction with newly prescribed loop or thiazide diuretics without dose adjustment or increased monitoring | - | [15] |
| | Patient treated with ECT in bipolar disorder and with lithium dose NOT stopped or reduced | Adult | [49] |
| | Patient treated with lithium in bipolar disorder does NOT have a serum level 0.8–1.1 mmol/L | Adult | [49] |
| | Patient on lithium in bipolar disorder and with lithium serum level [1.5 mmol/L) has lithium not discontinued | Adult | [49] |

| | | | |
|---|---|---|---|
| | In bipolar disorder, Patient who has discontinued lithium, does NOT have a recorded gradual reduction of lithium dose over at least 4 weeks | Adult | [49] |
| | Patient treated with divalproex in bipolar disorder does NOT have a serum level of 400–700 mmol/L | Adult | [49] |
| ***Non-Specific Psychotropics*** | Patient on a monotherapy regimen for the acute management of depressive bipolar disorder NOT taking Lithium OR Lamotrigine OR Quitiepine OR Divalproex OR Lurasidone OR Carbamazepine OR Olanzapine OR ECT | Adult | [49] |
| | Patient on a monotherapy regimen for the acute management of depressive bipolar disorder taking Gabapentin OR Aripiprazole OR Ziprasidone | Adult | [49] |
| | Patient on combination therapy for the Acute management of depressive bipolar disorder taking adjunctive Ziprasidone OR Levetiracetam | Adult | [49] |
| | Three or more psychotropic drugs on an as required (PRN) basis. | - | [22] |
| | Patient treated with lamotrigine and a second agent in bipolar disorder Is NOT prescribed Lithium OR Quetiapine OR Divalproex | Adult | [49] |
| | Patient treated with lithium and a second agent in bipolar disorder Is NOT prescribed | Adult | [49] |
| | Lamotrigine OR Quetiapine OR SSRI OR Bupropion OR Divalproex OR Olanzapine OR Risperidone OR MAOI OR Aripiprazole OR Ziprasidone OR Lurasidone OR Pramipexole OR Venlafaxine OR TCA" | Adult | [49] |
| | Patient treated with quetiapine and treated with a second agent in bipolar disorder Is NOT prescribed Lamtorigine OR SSRI OR Lithium OR Divalproex | Adult | [49] |

ECT: Electroconvulsive therapy. SSRI: Selective serotonin reuptake inhibitor. TCA: Tricyclic antidepressants.
