# Supplementary material for: Identifying potential prescribing safety indicators related to mental health disorders and medications: A systematic review
Source: PLoS One. 2019 May 24;14(5):e0217406. doi: 10.1371/journal.pone.0217406 (PMC6534318; doi:10.1371/journal.pone.0217406)
Supplement: S3 File — (PDF) [file pone.0217406.s003.pdf]

List of potential Prescribing Safety Indicators (PSIs) related to mental health medications and conditions

Table 1:PIM: Independent of Diagnoses or Conditions (Prescribing [Class/Medication] to a patient aged [age])

PIM: Independent of Diagnoses or Conditions			
**Therapeutic Category**	**Medication/Class**	**Age**	**References**
**Antipsychotics**	*Antipsychotics*	≥ 65	[25, 34, 38, 42, 43]
		0–5	[69]
**Antidepressants**	*Antidepressants*	0–5	[69]
	TCA	≥ 65	[32, 34, 38, 65]
		≤ 18	[103]
	SSRI other than fluoxetine	≤ 18	[73, 103]
	NDRI (Bupropion)	≥ 65	[46]
	NRI (Reboxetine)	≥ 65	[46]
	MAOi (Tranylcypromine)	≥ 65	[45, 46, 77]
**Sedative, hypnotics and anxiolytics**	*Benzodiazepine*	≥ 65	[25, 42, 43, 68]
	Z-drugs	≥ 65	[25, 42]
	Barbiturates	≥ 65	[39, 118]
	Meprobamate	≥ 65	[34, 39, 41, 46, 66, 118]
	Sedating antihistamine	≥ 65	[48]
	Promethazine	≥ 65	[34, 46, 47, 66, 91, 108, 118]
**ADHD medications**	*All ADHD Medications*	< 6	[103]
	Clonidine	≥ 65	[31, 34, 46, 48, 66, 91, 116]
	Guanfacine	≥ 65	[34, 46]
	Methylphenidate	≥ 65	[46, 91]
**Anticholinergics**	*Anticholinergics*	≥ 65	[38]

ADHD: Attention deficit hyperactivity disorder. MAOi: Monoamine oxidase inhibitor. NDRI: Norepinephrine–dopamine reuptake inhibitor. NRI: Norepinephrine reuptake inhibitor. SSRI: Selective serotonin reuptake inhibitor. TCA: Tricyclic antidepressants

Table 2: PIM: considering diagnoses or conditions (Prescribing [Class/Medication] to a patient with [Condition] +/- and aged [age])

PIM: considering diagnoses or conditions				
**Therapeutic Category**	**Condition**	**Medication/Class**	**Age**	**References**
**Antipsychotics**	Dementia but Not Psychosis	*Antipsychotics*	≥ 65	[73]
	BPSD	*Antipsychotics*	≥ 65	[25, 34]
	Seizures or Epilepsy	*Antipsychotics*	≥ 65	[116]
	Parkinson's Disease	*Antipsychotics other than quetiapine or clozapine*	≥ 65	[25, 32, 48, 73, 92]
	History of prostatism or previous urinary retention of BPH	*Antipsychotics*	-	[71]
	Glaucoma	*Fluphenazine*	-	[71]
		Perphenazine		[71]
		Trifluoperazine		[71]
	Syncope	*Antipsychotics*	≥ 65	[84]
	Postural Hypotension	*Antipsychotics*	≥ 65	[84]
	History of Falls	*Antipsychotics*	≥ 65	[34, 47, 84]
	Delirium	*Antipsychotics*	≥ 65	[34, 84]
	ADHD without Hyperactivity	*Antipsychotics*	Children	[103]
	Arrhythmia	*Antipsychotics*	≥ 65	[116]
	Lewy Body Disease	*Antipsychotics other than quetiapine or clozapine*	≥ 65	[25, 32]
	Chronic constipation	*Perphenazine*	≥ 65	[83]
		Clozapine		[83]
		Haloperidol		[83]
		Olanzapine		[83]
**Antidepressants**	Heart block	*TCA*	≥ 65	[88, 93, 116]
	Cardiac conduction abnormalities	*TCA*	≥ 65	[25, 43, 68]
	Cardiovascular risk factors or CVD	*TCA*	≥ 65	[92]

	Heart failure	*TCA*	-	[11, 71, 73]
	Arrhythmia	*Amitriptyline at dose >75mg*	-	[10]
	HTN	*Venlafaxine*	-	[71]
		Duloxetine		[71]
		MAOIs		[71]
	Postural hypotension	*TCA*	≥ 65	[88, 92, 93]
	Syncope	*TCA*	≥ 65	[47, 116]
	History of falls	*Amitriptyline*	≥ 65	[83]
		Clomipramine		[83]
		Imipramine		[83]
	Seizures or epilepsy	*SSRI*	-	[15, 71]
		TCA	-	[71]
	Dementia or cognitive impairment	*TCA*	-	[15]
	Glaucoma	*TCA*	-	[71]
		Mianserin	-	[71]
		MAOI	-	[71]
		Citalopram	-	[71]
		Escitalopram	-	[71]
		Fluoxetine	-	[71]
		Fluvoxamine	-	[71]
		Paroxetine	-	[71]
	Prostatism or history of urinary retention or BPH	*TCA*	-	[71]
	Constipation	*TCA*	≥ 65	[43, 47, 68, 88, 116]
	Current or recent significant hyponatraemia	*SSRI*	-	[15]
	Hepatic impairment or cirrhosis	*TCA*	-	[71]
	Gastrointestinal haemorrhage	*Paroxetine*	≥ 75	[85]
		Sertraline		[85]
		Fluvoxamine		[85]
		Escitalopram		[85]
	Peptic ulcer disease	*SSRI*	-	[71]
	Delirium	*Amitriptyline*	≥ 65	[83]
		Clomipramine		[83]
		Imipramine		[83]
	Acute bipolar depression	*TCA*	Adults	[49]
**Sedative, hypnotics and anxiolytics**	Dementia or cognitive impairment	*Benzodiazepines*	≥ 65	[34, 66, 84, 88]
	History of falls or fractures	*Sedative-hypnotics*	≥ 65	[88]
	Acute or chronic respiratory failure	*Benzodiazepines*	≥ 65	[25]
	Sleep apnoea syndrome	*Benzodiazepines*	≥ 65	[66, 116]
	Delirium	*Benzodiazepines*	≥ 65	[34, 84]
	BPH	*Antihistamine*	≥ 65	[116]
	Advanced dementia	*Antihistamine 1st generation*	-	[86]
	Hepatic impairment or cirrhosis	*Benzodiazepines*	-	[71]
	Chronic constipation	*antihistamines*	≥ 65	[116]
**Mood stabilisers**	Renal failure	*Lithium*	-	[71]
	Thyroid disorders	*Lithium*	-	[71]
	Epilepsy	*Lithium*	-	[71]
**Anti-dementia**	Persistent bradycardia	*Acetylcholinesterase inhibitors*	≥ 65	[25, 42]
	Heart block	*Acetylcholinesterase inhibitors*	≥ 65	[25]
	Recurrent unexplained syncope	*Acetylcholinesterase inhibitors*	≥ 65	[34]
**Anticholinergics**	Dementia or cognitive impairment	*Anticholinergics*	-	[39]
	Delirium	*Anticholinergics*	≥ 65	[25, 32, 34, 42]
	Chronic constipation	*Anticholinergics*	-	[71]
	Glaucoma	*Anticholinergics*	≥ 65	[25, 42, 47, 66, 88]

	History of urinary retention of BPH	*Anticholinergics*	-	[50]
**ADHD medications**	HTN	*Atomoxetine*	-	[71]
	Anorexia and malnutrition	*Methylphenidate*	≥ 65	[47]
	Epilepsy	*Methylphenidate*	-	[71]
	Insomnia	*Amphetamine*	≥ 65	[34]
		Methylphenidate		[34, 47, 84]
**Non-MH medication with MH condition**	Delirium	*H2-receptor antagonists*	≥ 65	[84]
		Corticosteroids		[34]
		Meperidine (Pethidine)		[34, 84]
	Insomnia	*Pseudoephedrine*	≥ 65	[34, 47, 84]
		Phenylephrine		[34, 47, 84]
		Armodafinil		[34]
		Modafinil		[34]
		Theophylline		[34, 84]
	Depression	*Methyldopa*	≥ 65	[116]
	Dementia or cognitive impairment	*Selegiline*	≥ 65	[101]

ADHD: Attention deficit hyperactivity disorder. BPH: Benign prostatic hyperplasia. BPSD: Behavioural and Psychological Symptoms of Dementia. CVD: Cardiovascular disease. HTN: Hypertension. MAOi: Monoamine oxidase inhibitor. MH: Mental health. SSRI: Selective serotonin reuptake inhibitor. TCA: Tricyclic antidepressants.

Table 3: Drug-drug interactions (Prescribing [Class/Medication] with [Class/Medication] +/- to a patient aged [age])

**Therapeutic Category**	**Drug-drug interactions**		**Age**	**References**
	**Medication/Class**	**Medication/Class**		
**Antipsychotics**	*Antipsychotics*	*Antipsychotic*	-	[22, 71]
		Antiparkinsonian agents	≥ 65	[44]
	Pimozide	*Macrolides antibiotics*	-	[89]
		Azole antifungal		[89]
**Antidepressants**	*TCA*	*MAO*	-	[15]
		TCA	≥ 65	[41, 112]
		Cimetidine	-	[111]
		Fluoxetine	-	[111]
		Fluvoxamine	≥ 70	[96]
		Paroxetine	-	[111]
		Selegiline	-	[102]
	SSRI	*Tramadol*	-	[15]
		Aspirin (no protection)	-	[15]
		MAOI	-	[89]
		SSRI's	≥ 65	[25, 43, 68]
		NSAID (no protection)	≥ 45	[70]
		Venlafaxine	≥ 45	[70]
		Vitamin K antagonists	-	[71]
		Selegiline	-	[111]
	MAO	*Tramadol*	-	[15]
		Dextromethorphan	-	[89]
		Amphetamine and derivatives	-	[102]
		Narcotic analgesics	-	[102]
		Triptans	-	[102]
		Levodopa	-	[104]
		MAOI	≥ 65	[41, 112]
	Amitriptyline	*Sertraline*	children	[79]
		Trazodone		[79]
	Citalopram	*QT-prolonging drugs*	-	[15]
	Citalopram	*Linezolid*	children	[79]
	Sertraline			[79]
	Fluvoxamine	*Theophylline*	-	[89]
		Ramelteon		[102]
	Trazodone	*anti-HCV antivirals*	-	[71]
	Escitalopram			[71]
	Hypnotic or sedative	*Hypnotic or sedative*	≥ 65	[32, 41, 112]
	Benzodiazepine	*Azole antifungal agents*	-	[89]

**Sedative, hypnotics and anxiolytics**		*Cimetidine*	≥ 65	[116]
		Benzodiazepines	-	[71]
	Alprazolam	*Strong CYP3A4 inhibitor*	≥ 65	[48]
	Midazolam			[48]
	Triazolam			[48]
	Flurazepam	*Anti-HCV antivirals*	-	[71]
	Guazepam			[71]
	Triazolam			[71]
	Alprazolam			[71]
	Zolpidem	*Strong CYP3A4 inhibitor*	≥ 65	[48]
	Zopiclone			[48]
	Zolpidem	*Anti-HCV antivirals*	-	[71]
**Mood stabilisers**	*Valproic acid*	*Carbapenems*	-	[104]
		Lamotrigine	Children	[79]
	Carbamazepine	*Clarithromycin*	-	[109]
		Erythromycin	≥ 45	[70]
		Cimetidine	≥ 65	[116]
		oral or intravaginal contraceptives, patches or pure progestogen pills	-	[71]
		Warfarin	-	[111]
		Propoxyphene	-	[89, 111]
		Rivaroxaban	-	[71]
	Lithium	*ACEi*	≥ 65	[34]
		NSAID	≥ 65	[48]
		Diuretics	-	[49]
	Lamotrigine	*Hormonal contraceptive or combination pills*	-	[71]
**Anti-dementia**	*Anticholinesterase drugs*	*Anticholinergic*	≥ 65	[77]
		Anticholinesterase drugs	-	[71]
**Anticholinergic**	*Anticholinergic*	*Anticholinergic*	≥ 65	[25, 32, 34, 48]
**ADHD medications**	*Clonidine*	*Propranolol*	-	[111]

ADHD: Attention deficit hyperactivity disorder. ACEi: Angiotensin-converting-enzyme inhibitor. CYP: Cytochrome P450. HCV: Hepatitis C virus. MAOi: Monoamine oxidase inhibitor. NSAID: Nonsteroidal anti-inflammatory drugs. SSRI: Selective serotonin reuptake inhibitor. TCA: Tricyclic antidepressants.

Table 4: Inappropriate Duration (Prescribing [Class/Medication] for [duration] +/- to a patient with [condition] +/- and aged [age])

Therapeutic Category	Class/Medication	Inappropriate Duration<br>Condition	Duration	Age	References
**Antipsychotics**	*Antipsychotics*	Dementia but not psychosis	>6 weeks	≥ 65	[10]
		as long-term hypnotics	>1 month	≥ 65	[43, 47, 77]
	More than one Antipsychotics	-	>2 month	Adults	[69]
**Antidepressants**	*Three or more Antidepressants*	-	>3 month	adults	[69]
	More than one SSRI	-	>2 month	adults	[69]
	SSRI and SNRI combination	-	>2 month	adults	[69]
**Sedative, hypnotics and anxiolytics**	*Hypnotics*	-	>1 month	-	[115]
	Benzodiazepine	-	>1 month	-	[15, 99]
	Z-drugs	-	>1 month	-	[15]
	First-generation antihistamine	-	> 1 week	≥ 65	[43, 47]
**Non-Specific Psychotropics**	*Four or more Psychotropics*	-	>3 months	6–17	[69]
**Non-MH medication with MH condition**	*Opioids*	Dementia (unless palliative)	long term	≥ 65	[43]

MH: Mental health. SNRI: Serotonin and norepinephrine reuptake inhibitors. SSRI: Selective serotonin reuptake inhibitor.

Table 5: Inappropriate dose (Prescribing [Medication] at a dose [Dose] +/- to a patient with [Condition] +/- and aged [Age])

Therapeutic Category	Inappropriate dose				
	**Medication**	**Dose**	**Condition**	**Age**	**References**
**Antipsychotics**	*Haloperidol*	>2 mg/day		≥ 65	[45-47]
	Risperidone	3 mg/day	BPSD: restlessness, agitation	≥ 65	[101]
	Antipsychotics	High dose *Total daily dose is above the maximum recommended by the British National Formulary (BNF)*		-	[22]
**Antidepressants**	*Fluoxetine*	>40 mg/day		≥ 65	[41]
	Imipramine	>100 mg/day		≥ 65	[41]
	Trimipramine	>100 mg/day		≥ 65	[41]
**ADHD medications**	*SR Methylphenidate*	two doses per day, rather than one dose		Children	[103]
**Sedative, hypnotics and anxiolytics**	*Alprazolam*	>2 mg/day		≥ 65	[39, 47, 68]
	Brotizolam	>0.125 mg/day		≥ 65	[45, 46]
	Gabapentin	>1400mg/day	CrCl 30-59 mL/min	-	[72]
	Gabapentin	>700mg/day	CrCl 15-29 mL/min	-	[72]
	Gabapentin	>300mg/day	CrCl 10-14 mL/min	-	[72]
	Gabapentin	>150mg/day	CrCl < 10 mL/min	-	[72]
	Lorazepam	> 2 mg/day		≥ 65	[45]
	Lormetazepam	>0.5 mg/day		≥ 65	[45, 46]
	Oxazepam	>60 mg/day		≥ 65	[39, 45, 46, 68]
	Pregabalin	>300mg/day	CrCl 30-59 mL/min	-	[72]
	Pregabalin	>150mg/day	CrCl 15-29 mL/min	-	[72]
	Pregabalin	>75mg/day	CrCl < 15 mL/min	-	[72]
	Temazepam	>15 mg/day		≥ 65	[39, 68]
	Triazolam	>0.25 mg/day		≥ 65	[39, 66, 68]
	Zaleplon	>5 mg/day		≥ 65	[45, 46]
	Zolpidem	>5 mg/day		≥ 65	[45-47]
	Zopiclone	>7.5mg/day		≥ 70	[96]

ADHD: Attention deficit hyperactivity disorder. BPSD: Behavioural and Psychological Symptoms of Dementia. CrCl: Creatinine clearance.

Table 6: Monitoring (Prescribing [Class/Medication] with monitoring [Test] [Frequency])

Therapeutic Category	Monitoring				
	**Medication/Class**	**Test**	**Age**	**Frequency**	**References**
**Antipsychotics**	*Antipsychotics*	Glucose	-	Annually	[39]
				3-4 months after starting therapy	[49]
		Weight	-	Annually	[39]
				3-4 months after starting therapy	[49]
		Lipid profile	-	3 months after starting therapy	[49]
	Clozapine	WBC	-	NR	[117]
**Mood stabilisers**	*Carbamazepine*	LFT	-	Annually	[81]
		FBC	-	Annually	[81, 105]
		Carbamazepine level	-	Every 6 months	[50]
	Valproate	LFT	-	Annually	[81]
				first 6 months of therapy	[49]
		FBC	-	Annually	[81, 113]
				First 6 months of therapy	[49]
	Lithium	lithium level	-	every 3 months	[17, 61]
		TFT	-	every 6 months	[49]
		Creatinine	-	annually	[81, 105]
**ADHD medications**	*Methylphenidate*	Growth chart (height and weight)	Children	NR	[103]

ADHD: Attention deficit hyperactivity disorder. FBC: Full blood count. LFT: Liver function test. NR: Not reported. TFT: Thyroid function test. WBC: White blood fount

Table 7: Omission (Patient with [Condition +/- Age] NOT prescribed [Class/Medication]

Omission				
**Therapeutic Category**	**Medication/Class**	**Condition**	**Age**	**References**
**Anti-dementia**	*Acetylcholinesterase inhibitor*	Mild- moderate Alzheimer's dementia	≥ 65	[25]
		Lewy Body dementia		[25]
**Antidepressants**	*Antidepressants*	moderate/severe depressive symptoms lasting at least three months	≥ 65	[43]
	SSRI	Persistent severe anxiety that interferes with independent functioning.	≥ 65	[25]
**Mood stabilisers**	*Mood stabilisers*	on antidepressants for acute bipolar depression	Adult	[49]

SSRI: Selective serotonin reuptake inhibitor

Table 8: Other

Therapeutic Category	Indicator	Age	References
**Antidepressants**	TCA except in case of severe depression or in low dose for neuropathic pain	≥ 65	[42]
	Patient diagnosed with acute bipolar depression is prescribed antidepressant monotherapy	Adult	[49]
**Antipsychotics**	Risperidone continued following discharge without follow-up to a patient with dementia	≥ 75	[107]
**Mood stabilisers**	Lithium dose not adjusted or omitted in a patient with a lithium concentration above the therapeutic range (>1. - mmol l-1)	-	[15]
	Lithium prescribed in conjunction with newly prescribed nonsteroidal anti-inflammatory drugs without dose adjustment or increased monitoring	-	[15]
	Lithium therapy prescribed in conjunction with newly prescribed loop or thiazide diuretics without dose adjustment or increased monitoring	-	[15]
	Patient treated with lithium in bipolar disorder does NOT have a serum level 0.8–1.1 mmol/L	Adult	[49]
	Patient on lithium in bipolar disorder and with lithium serum level [1.5 mmol/L) has lithium not discontinued	Adult	[49]
	In bipolar disorder, Patient who has discontinued lithium, does NOT have a recorded gradual reduction of lithium dose over at least 4 weeks	Adult	[49]
**Non-Specific Psychotropics**	Three or more psychotropic drugs on an as required (PRN) basis.	-	[22]

TCA: Tricyclic antidepressants.
